# Ion channel gene expression predicts survival in glioma patients

**DOI:** 10.1038/srep11593

**Published:** 2015-08-03

**Authors:** Rong Wang, Christopher I. Gurguis, Wanjun Gu, Eun A Ko, Inja Lim, Hyoweon Bang, Tong Zhou, Jae-Hong Ko

**Affiliations:** 1 Department of Oncology, The First Affiliated Hospital of Soochow University, Suzhou, Jiangsu 215006, China; 2 Department of Medicine, University of Arizona, Tucson, AZ 85721, USA; 3 Research Center for Learning Sciences, Southeast University, Nanjing, Jiangsu 210096, China; 4 Department of Pharmacology, University of Nevada School of Medicine, Reno, NV 89557, USA; 5 Department of Physiology, College of Medicine, Chung-Ang University, Seoul 156-756, South Korea

## Abstract

Ion channels are important regulators in cell proliferation, migration, and apoptosis. The malfunction and/or aberrant expression of ion channels may disrupt these important biological processes and influence cancer progression. In this study, we investigate the expression pattern of ion channel genes in glioma. We designate 18 ion channel genes that are differentially expressed in high-grade glioma as a prognostic molecular signature. This ion channel gene expression based signature predicts glioma outcome in three independent validation cohorts. Interestingly, 16 of these 18 genes were down-regulated in high-grade glioma. This signature is independent of traditional clinical, molecular, and histological factors. Resampling tests indicate that the prognostic power of the signature outperforms random gene sets selected from human genome in all the validation cohorts. More importantly, this signature performs better than the random gene signatures selected from glioma-associated genes in two out of three validation datasets. This study implicates ion channels in brain cancer, thus expanding on knowledge of their roles in other cancers. Individualized profiling of ion channel gene expression serves as a superior and independent prognostic tool for glioma patients.

Ion channels are membrane proteins that open or close the plasma membrane depending on voltage gradient or binding of ligands. They influence important physiological functions, including hormone secretion, muscle contraction, immune response, regulation of cell volume, cell migration, and cell proliferation^1^. Given their crucial roles in various fundamental biological processes, the aberrant expression of ion channels is linked to many genetic disorders^2^, such as human hyperkalaemic periodic paralysis^2^, paramyotonia congenita^3^, episodic ataxia^4^, familial hemiplegic migraine^5^, and cystic fibrosis^6^. Ion channels have also been implicated in tumor growth, apoptosis, and metastasis in human cancers^7^. For instance, down-regulation of the voltage-gated Ca^2+^ channel subunit encoded by the gene *CACNA2D3* could contribute to the development of metastasis in breast cancer^8^, high expression level of the voltage-gated K^+^ channel Kv11.1 (encoded by gene *KCNH2*) is correlated with invasiveness of colon cancer^9^, and the ligand-gated nicotinic acetylcholine receptor α5-nAchR (encoded by gene *CHRNA5*) is involved in nicotine-induced tumor cell proliferation in non-small cell lung cancer^10^. Recently, several studies have demonstrated the prognostic value of the expression profiling of ion channel genes in outcome prediction in multiple human carcinomas, including breast, colon, and lung cancers^11^,^12^,^13^. More importantly, the predictive power of ion channel genes is independent of standard clinical and pathological prognostic factors in human cancer. We hypothesize that malfunction and/or aberrant expression of ion channels may be a common feature of cancers and examine patterns of ion channel gene expression in glioma.

Glioma, a type of cancer that originates from glia cells, accounts for approximately 80 percent of all malignant brain tumors^14^. Glioma is divided into four grades (I–IV) by the World Health Organization (WHO) according to the relative malignancy. Grades I and II are designated as low-grade glioma, while grades III and IV are designated as high-grade and are usually regarded as malignant. These high-grade gliomas are comprised of glioblastoma, anaplastic astrocytoma, mixed anaplastic oligoastrocytoma (AOA), and anaplastic oligodendroglioma (AOD)^15^. Ion channels have been reported to be widely expressed in glia cells^16^ and play a critical role in malignant glioma^17^,^18^. First, through their effects on the shape and volume of glioma cells, ion channels may influence tumor invasion and migration^17^. For example, reduced membrane expression of a member of the voltage-gated Cl^-^ channels, ClC-3 (encoded by gene *CLCN3*), inhibits migration of glioma cells *in vitro* and *in vivo*^19^. Second, ion channels may affect the proliferation of glioma cell. For example, voltage-dependent large-conductance Ca^2+^ -activated K^+^ channels, often referred to as Big Potassium (BK) channels, were found to play a key role in growth control of human glioblastoma cells^20^. Third, ion channels may regulate the apoptosis of glioma cells. Inhibition of outwardly rectifying K^+^ channels was reported to cause apoptosis in malignant glioma cells^21^.

Despite the advances in therapeutic approaches, patients with malignant glioma have short survival time, especially for glioblastoma with a median survival of approximately twelve months^22^. Therefore, several molecular markers have been proposed to predict survival in glioma, including both microRNA^23^,^24^ and protein-coding gene^25^,^26^ signatures. Our previous work has suggested that ion channel gene expression is a novel genomic biomarker in predicting the outcome of several human carcinomas^11^,^12^,^13^. In this study, we profile expression of ion channel genes to predict glioma outcome. We identify a prognostic molecular signature, which includes expression patterns of 18 ion channel genes. 16 of these 18 ion channel genes (89%) were down-regulated in high grade gliomas. This signature successfully distinguishes glioma patients with high death-risk from the ones with low risk. This signature is also independent of and improves on traditional prognostic factors in glioma. These results highlight the utility of ion channel genes as valuable biomarkers for glioma outcome prediction and for potentially facilitating individualized therapies in this disease.

## Results

### Ion channel gene expression is correlated with WHO grade of glioma

**Figure 1:**
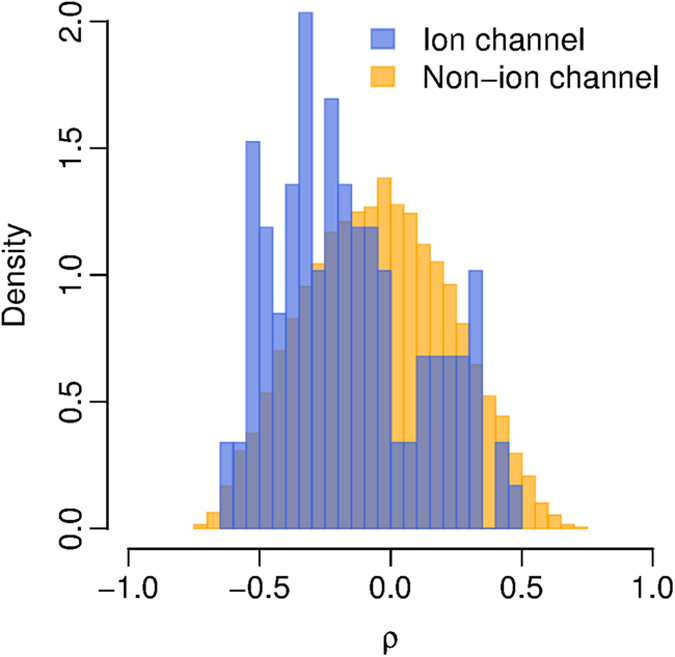
Distribution of the correlation coefficient (*ρ*). Spearman’s rank correlation test was conducted between the gene expression level and WHO glioma grade. The blue histogram is for the ρ of ion channel genes while the yellow histogram is for the ρ of non-ion channel genes.

**Figure 2:**
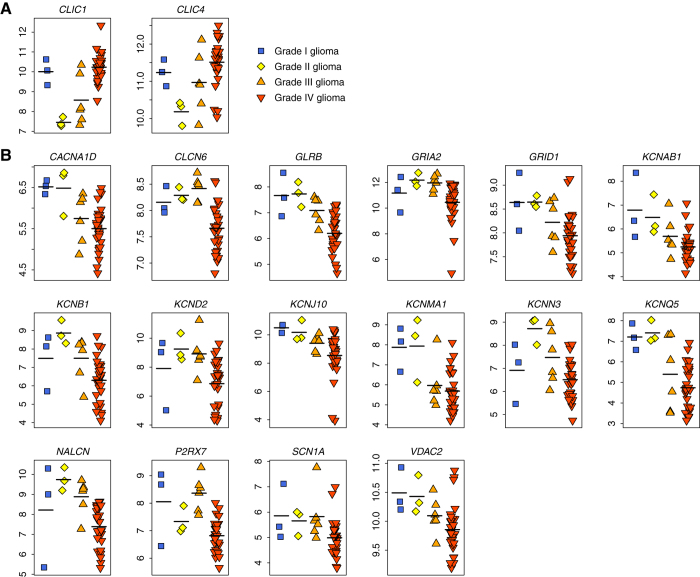
Deregulated ion channel genes in glioma. The gene expression data were from the UHC cohort. (**A**) The two ion channel genes that are up-regulated in high-grade glioma. (**B**) The 16 ion channel genes that are down-regulated in high-grade glioma. For the gene with multiple probe sets, only the probe set with the most significant *P*-value was plotted. The horizontal black line indicates the mean of each category. X-axis: WHO glioma grade; Y-axis: log_2_-transformed expression values.

We categorized the severity of gliomas using the WHO’s grading system. Grade I glioma is the least severe with the best prognosis while grade IV is the most severe carrying the worst prognosis. To identify the genes that are associated with the severity of glioma, we downloaded a high-throughput gene expression dataset from the Gene Expression Omnibus (GEO) database (GEO accession: GSE43289), which was based on the Affymetrix Human Genome U133 Plus 2.0 Array. The patients of this cohort were from the University Hospital of Coimbra (UHC), Portugal^27^. Forty glioma patients with annotated WHO tumor grade were considered in this study, which included 3 grade I, 3 grade II, 6 grade III, and 28 grade IV patients. One possibility to explain the differences in sample sizes is that glioma is asymptomatic until it has progressed to higher grade, as evinced by the fact that grade III and grade IV are the most commonly diagnosed grade of glioma^28^. For the Affymetrix microarray, only the well-annotated probe sets with a “present” call in at least two third of the samples were retained. In total, 18,041 probe sets encoding 10,385 genes were considered in this study, which included 108 probe sets encoding 84 ion channel genes. Spearman’s rank correlation test was used to identify the genes in which the gene expression level was significantly correlated with glioma grade. Figure 1 shows the distribution of the correlation coefficient (*ρ*) for all the genes. The *ρ* of ion channel gene expression profiles is significantly more negative than the non-ion channel gene expression profiles (t-test: *P* = 4.9 × 10^−6^) (Fig. 1). In total, we found that 2,559 probe sets encoding 1,913 genes are differentially expressed with glioma grade (Spearman’s rank correlation test: adjusted *P* < 0.05 after Benjamini & Hochberg correction). Among these probe sets, 842 sets are up-regulated, while 1,717 sets are down-regulated in high-grade glioma (Supplementary Table S1). Among the deregulated probe sets, only two probe sets encoding two ion channel genes, *CLIC1* and *CLIC4* (both encode chloride intracellular channel), are up-regulated in high-grade glioma (Fig. 2A and Supplementary Table S2). In contrast, 22 probe sets encoding 16 ion channel genes, including both voltage-gated ion channels and ligand-gated channels, are down-regulated in high-grade glioma (Fig. 2B and Supplementary Table S2). Among the down-regulated probe sets, there is a significant enrichment of ion channel genes in high-grade glioma (Fisher’s exact test: *P* = 1.5 × 10^−3^).

To validate the above findings, we analyzed an independent gene expression dataset (GEO accession: GSE4290) from the Henry Ford Hospital (HFH)^29^, containing 23 non-neoplastic samples and tumor samples from 45 grade II, 31 grade III, and 77 grade IV glioma patients. The expression pattern of ion channel genes in the HFH cohort mirrored our findings in the UHC cohort: gene expression level and glioma grade were significantly correlated (Spearman’s rank correlation test: adjusted *P* < 0.05 after Benjamini & Hochberg correction) for all the deregulated ion channel genes identified from the UHC cohort (Supplementary Fig. S1 and Table S3).

### Expression of ion channel genes predicts survival in glioma patients

We identified 18 ion channel genes deregulated with glioma. Here, we predicted that the expression of these 18 ion channel genes could be used for prognostic purpose in glioma. We assigned the 18 ion channel genes as an ion Channel based Gene (iCG) signature (Table 1 and Fig. 2). Of these 18-ion channel genes, 16 genes (89%) were down-regulated in high grade gliomas. A weight was assigned to each gene among iCG signature according to the direction of differential expression: 1 for the up-regulated and -1 for the down-regulated genes in high-grade glioma. A risk score was assigned to each patient based on the iCG signature and gene weight (see Methods for details). A more positive risk score was meant to presage a higher death risk.

Next, we tested whether the iCG-based risk score was able to predict survival in glioma. For this purpose, we obtained three independent high-throughput gene expression datasets online: i) a cohort including 95 high-grade glioma patients (GEO accession: GSE43107) from the European Organisation for Research and Treatment of Cancer (EORTC)^30^, ii) a cohort composed of 77 high-grade glioma patients (GEO accession: GSE4271) from the M.D. Anderson Cancer Center (MDACC)^31^, and iii) a cohort consisting of 50 high-grade glioma patients (http://www-genome.wi.mit.edu/cancer/pub/glioma/) from the Massachusetts General Hospital (MGH)^32^. These datasets were chosen based on the large number of samples (sample size ≥ 50) and the availability of clinical outcome data.

**Figure 3:**
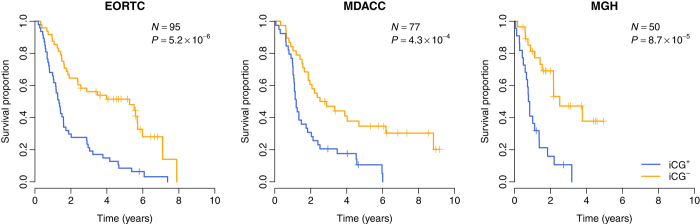
Kaplan-Meier curves for glioma patients in the three validation cohorts. The expression of iCG predicts poor survival in the EORTC, MDACC, and MGH cohorts. The blue curves are for the iCG^+^ patients while yellow curves are for the iCG^-^ patients. *P*-values were calculated by log-rank test.

We defined iCG^+^ patients as those having a risk score larger than zero while the other patients were assigned as iCG^-^. Kaplan-Meier survival curves indicate that there is a significant difference in survival between the iCG^+^ and iCG^-^ glioma patients in all the validation cohorts (log-rank test: *P* = 5.2 × 10^−6^ for the EORTC cohort; *P* = 4.3 × 10^−4^ for the MDACC cohort; and *P* = 8.7 × 10^−5^ for the MGH cohort) (Fig. 3). The association between iCG risk score and survival was also confirmed by univariate Cox proportional hazard regression of survival. The iCG^+^ patients have a 2.84-, 2.51-, and 3.95-fold increased risk of death in the EORTC cohort, the MDACC cohort, and MGH cohort, respectively (Table 2).

We divided iCG into two subsets: iCG-up (the two ion channel genes that are up-regulated in high-grade glioma) and iCG-down (the 16 ion channel genes that are down-regulated in high-grade glioma). We tested the prognostic power of iCG-up and iCG-down separately. Kaplan-Meier survival curves indicate that there is a significant difference in survival between the iCG-up^+^ and iCG-up^-^ glioma patients in the MDACC and MGH cohorts (log-rank test: *P* = 6.4 × 10^−3^ for the MDACC cohort; and *P* = 6.7 × 10^−3^ for the MGH cohort), but not in the EORTC cohort (log-rank test: *P* = 1.3 × 10^−1^) (Supplementary Fig. S2). As for iCG-down, we found that the iCG-down^+^ patients have a significantly increased risk of death in all the validation cohorts (log-rank test: *P* = 1.8 × 10^−5^ for the EORTC cohort; *P* = 8.7 × 10^−4^ for the MDACC cohort; and *P* = 1.7 × 10^−4^ for the MGH cohort) (Supplementary Fig. S3).

### iCG is better than the random gene signatures picked up from human genome

**Figure 4:**
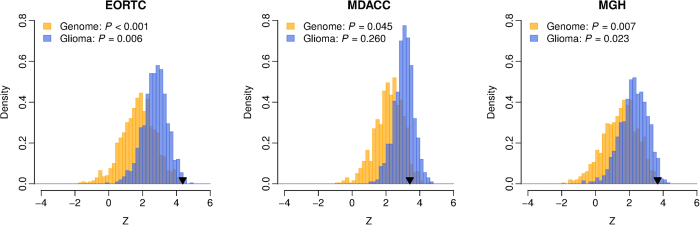
Superior prognostic power of iCG compared with random gene signature. The yellow histogram shows the distributions of the Wald statistic (*Z*) for the 1,000 resampled gene signatures picked up from human genome with identical size as iCG. The blue histogram shows the distributions of the *Z* for the 1,000 resampled gene signatures selected from the glioma-associated genes. The black triangles stand for the *Z* values of iCG. Right-tailed *P*-values of the sampling distribution were calculated.

A computational study by Venet *et al.* demonstrated that, in breast cancer, most published prognostic gene signatures were not significantly better than random gene sets of identical size that were randomly selected from human genome^33^. To address this issue in our study, we conducted a resampling test for the iCG signature. We obtained 1,000 random gene signatures by randomly selecting 18 genes from human genome (the same size as the iCG signature). For each random gene signature, we calculated the risk score for each glioma patient and performed univariate Cox proportional hazard regression of survival to evaluate the association between the random gene signature and glioma clinical outcome. The Wald statistic (*Z*), the ratio of Cox regression coefficient to its standard error was recorded for each random gene signature. This ratio indicates the significance level of the relationship between survival and the risk score. Our alternative hypothesis was that the *Z* of iCG should be more positive than expected by chance if the prognostic power of iCG was significantly better than the random gene signatures. We found that, in all the validation cohorts, we could reject the null hypothesis that the association between iCG and survival is by chance. The *Z* of iCG is significantly larger than that of the random gene signatures (Right-tailed: *P* < 0.001 for for the EORTC cohort; *P* = 0.045 for the MDACC cohort; and *P* = 0.007 for the MGH cohort) (Fig. 4).

### iCG performs better than the random gene signatures picked up from glioma-associated genes

Next, we asked whether the prognostic power of iCG is superior to the other genes that are associated with glioma by conducting a second resampling test. We limited the resampling pool to the genes that were differentially expressed with glioma grade (Supplementary Table S1) and defined these genes as glioma-associated. We then randomly selected 18 genes from the pool of glioma-associated genes and tested the predictive power of this random gene signature. The performance of the random gene signature was quantified by the Wald statistic (*Z*) computed by univariate Cox proportional hazard regression of survival. We found that the prognostic power of iCG is significantly better than that of 1,000 random glioma-associated gene signatures in the EORTC and MGH cohorts (Right-tailed: *P* = 0.006 for for the EORTC cohort; and *P* = 0.023 for the MGH cohort) (Fig. 4), but not in the MDACC cohort (Right-tailed: *P* = 0.260) (Fig. 4).

### iCG is an independent prognostic factor

Using multivariate Cox proportional hazard regression, we tested the performance of iCG in comparison with the other prognostic factors associated with glioma outcome. Due to the limitation of available patient medical data, we were unable to consider the MDACC and MGH cohorts. Only the EORTC cohort was investigated here. The EORTC cohort was the largest dataset in this study, which was mainly composed of AOA and AOD patients. First, we considered clinical factors, including age, gender, type of surgery, and performance status, molecular factors, such as loss of heterozygosity (LOH) on chromosome 1p and 19q, and histological factors (AOA or AOD). Here, type of surgery was categorized into biopsy, partial resection, and total resection and encoded as 1, 2, and 3, respectively. Performance status was based on the Eastern Cooperative Oncology Group standard^30^,^34^. In total, we identified 89 glioma patients without missing data. Multivariate Cox proportional hazards regression of survival indicated that the iCG status is the most significant covariate in relation to the other clinical and pathological factors (Table 3). Second, we added more molecular prognostic factors into the multivariate test, including epidermal growth factor receptor (*EGFR*) amplification, isocitrate dehydrogenase 1 (*IDH1*) mutation, and O-6-methylguanine-DNA methyltransferase (*MGMT*) promoter methylation. Due to missing observations, only 53 patients were included. Multivariate Cox proportional hazards regression of survival demonstrated that the iCG status is still the most significant factor in the new multivariate model (Table 4).

Mutations in *IDH1* are among the key events in the formation of diffuse gliomas and associated with prolonged survival. Here, we also found that the *IDH1* mutation status was one of the significant prognostic covariate in the multivariate model (Table 4). Therefore, we further stratified the patients according to the *IDH1* mutation status and repeated the Cox proportional hazards regression. For patients with and without *IDH1* mutation, the iCG^+^ patients have a 3.91- and 3.32-fold increased risk of death, respectively (Cox proportional hazard regression: *P* = 3.2 × 10^−3^ for patients without mutation; and *P* = 4.9 × 10^−3^ for patients with mutation). Kaplan-Meier survival curves also demonstrated significantly reduced survival for the iCG^+^ patients in each subset grouped by the *IDH1* mutation status (Supplementary Fig. S4).

## Discussion

Because of their highly influential role in central biological processes (e.g. cell signaling, motility, and proliferation), ion channel genes have been implicated in a wide variety of disease processes^2^,^3^,^4^,^5^,^6^. In particular, the role of ion channels in cancer pathology has been heavily documented in breast^11^,^35^, lung^12^,^36^, colon^13^,^37^, and skin^38^,^39^ cancers. In this study, we identified a prognostic gene signature composed of 18 ion channel genes (iCG), which successfully predicted glioma outcome in three independent validation cohorts. We therefore expand knowledge of the link between deregulation of ion channel gene expression and cancer by examining this link within glioma patients and suggest that deregulation of ion channel genes may be a general feature of cancer pathology (see also Lastraioli *et al.*^7^). In sum, our results indicate that i) ion channels play an important role in the pathology of glioma, ii) ion channels generally tend to be down-regulated in high-grade glioma (only 2 of 18 genes were up-regulated here), and iii) iCG is a superior and independent covariate, which adds prognostic value to traditional clinical and pathological factors.

The explicit link between the deregulation of ion channel expression in glioma and prognosis of glioma patients adds to a growing body of evidence that alterations to ion channel gene expression may be a common feature in various cancers^7^. Indeed, *CACNA1D*^40^, the intracellular chloride channel genes^41^, *GRIA2*^42^, the potassium channel genes^43^, *NALCN*^44^, *P2RX7*^45^, *SCN1A*^46^, and *VDAC1*^47^ from iCG are all under investigation in cancer therapies in some capacity. This indicates that at least 15 of the 18 genes in iCG (83%) are already recognized for their potential in cancer treatments. More work is needed to determine whether the other genes in our signature could be exploited for cancer therapy.

We conducted Spearman’s correlation test between gene expression level and WHO glioma grade. We found that the correlation coefficient for the ion channels genes were statistically more negative than that of the other genes. More interestingly, 16 out of 18 (89%) ion channel genes in iCG, including Ca^2+^, K^+^, Na^+^, and Cl^–^ channels, were down-regulated in high-grade glioma. All these results suggest that high-grade glioma expresses fewer ion channels compared with low-grade tumors, which is consistent with the previous findings in voltage-gated Na^+^^48^ and K^+^^49^ channels. Intriguingly, however, this bias toward down-regulation is not reported in other studies linking ion channels to cancer^11^,^12^,^13^. More work is needed to understand what mechanisms produce this pattern.

We also found a contradictory expression pattern for the BK channel. BK channels are essential for the regulation of several key physiological processes, which are especially fundamental to the control of neuronal excitability^50^. BK currents in glioma cells were found to be more sensitive to intracellular Ca^2+^ concentration compared with that in normal glial cells^51^,^52^. BK channels have been found to be up-regulated in biopsies of high-grade glioma^17^. Also, a positive correlation was detected between BK channel expression and malignancy grade of glioma^53^. The BK channel gene, *KCNMA1*, is among the iCG gene list. However, the weight of *KCNMA1* is negative in this study (Table 1), which means the gene expression of *KCNMA1* is negatively correlated with glioma grade in the UHC cohort. To double-check the expression pattern of *KCNMA1*, univariate Cox proportional hazards regression was conducted to estimate the relationship between glioma survival and *KCNMA1* expression level in the three validation cohorts. Interestingly, we observed that the survival time of glioma was inversely and significantly correlated (hazard ratio <1) with *KCNMA1* expression level in all three validation datasets (Table 5), which is consistent with the finding from the UHC cohort. Therefore, the down-regulation of *KCNMA1* in malignant glioma is unlikely by chance. Seeking the reason for the opposite observations for BK channel is beyond the scope of current study. However, our finding suggests that the role of BK channel in glioma is vexed and further extensive investigation is needed.

A published bioinformatical study by Venet *et al.* demonstrated that most published prognostic gene signatures of breast cancer are not more strongly associated with cancer survival than random gene sets^33^. Venet *et al.* compared 47 prognostic breast cancer signatures to the signatures composed of random genes and found that roughly 60% of the published signatures were not significantly better than the randomized gene signatures of identical size^33^. This important finding reminds us that the strength of a putative gene signature to predict survival outcomes must be tested explicitly, since many randomly-generated gene signatures could also predict survival. Using resampling tests, we found that the prognostic power of iCG is better than that of the random gene sets selected from human transcriptome in all the validation cohorts. More importantly, we demonstrate that iCG performs even better than the random gene signatures selected from glioma-associated genes in two out of three validation datasets. Therefore, it is reasonable to conclude that the iCG signature overcomes the problem raised by Venet *et al.*

Finally, our analyses add to the growing body of evidence that cancer is a disease under quantitative genetic and genomic control^54^. Although there are loci of major effect in cancer (e.g. oncogenes and tumor suppressor genes), likely many loci of small effect also contribute to carcinogenesis and metastasis. The contribution of many genes (as well as other, non-genetic mechanisms^55^) to the cancer disease process not only makes development of effective cancer treatments difficult, but also means that researchers should examine cancer as any other complex trait. The fact that cancer is a complex trait also means that there are many potential therapeutic targets than can be exploited in the future. These may enhance both our understanding of cancer as a biological phenomenon as well as provide the means to overcome particularly intractable problems in cancer therapy such as development of chemoresistance.

This study confirms the central role of ion channels in brain cancer despite a clear molecular mechanism. The expression profiling of ion channel genes serves as a significant and independent tool for glioma outcome prediction. When working cooperatively with known clinical, molecular, and histological prognostic factors, the iCG signature will enhance the prediction accuracy for identifying glioma patients at higher risk for death. Our study also suggests that ion channels may serve as potential drug targets in future cancer therapy.

## Methods

### Ion channel genes

The definition of ion channel genes was obtained from Ko *et al.*^11^, and included Ca^2+^-activated Cl^–^ channels, Cl^–^ intracellular channels, voltage-sensitive Cl^–^ channels, mid-1-related Cl^–^ channel, Ca^2+^-activated K^+^ channels, voltage-gated K^+^ channels channels, voltage-gated Ca^2+^ channels, voltage-gated Na^+^ channels, two-pore K^+^ channels, CatSper and two-pore channels, inwardly rectifying K^+^ channels, non-voltage-gated Na^+^ channels, transient receptor potential channels, cyclic nucleotide-regulated channels, GABA_A_ receptors, 5-HT_3_ receptors, glycine receptors, ionotropic glutamate receptors, nicotinic acetylcholine receptors, P2X receptors, voltage-dependent anion channels, voltage-gated proton channel, voltage-independent cation channel, and zinc-activated ligand-gated ion channel^11^.

### High-throughput gene expression data

Five independent glioma datasets, including the UHC (GEO accession: GSE43289)^27^, HFH (GEO accession: GSE4290)^29^, EORTC (GEO accession: GSE43107)^30^, MDACC (GEO accession: GSE4271)^31^, and MGH (http://www-genome.wi.mit.edu/cancer/pub/glioma/)^32^ cohorts, were collected in this study. The UHC and HFH cohorts, which are based on Affymetrix Human Genome U133 Plus 2.0 Array, were used to measure the correlation between gene expression level and glioma grade. The EORTC, MDACC, and MGH cohorts were based on Affymetrix Human Exon 1.0 ST Array, Affymetrix Human Genome U133A/B Array, and Affymetrix Human Genome U95 Version 2 Array, respectively, which were used to validate the prognostic power of iCG.

The robust multi-array average (RMA) function in the “affy” package of Bioconductor^56^ was used to summarize the expression level of each probe set for the microarray data from the UHC, HFH, MDACC, and MGH cohorts. For the UHC dataset, the function “mas5calls” in the “affy” package^57^ was used to compute the present/absent call for each probe set. For the EORTC cohort, the gene expression values were summarized using the Affymetrix Power Tools Version 1.15.0 (http://www.affymetrix.com/). We limited our analysis to the probe sets with unique annotations. Genes on chromosomes X and Y were removed to avoid the potential confounding factors. For the gene with multiple probe sets, we used the geometric mean of expression values of all probe sets that mapped to the gene in the three validation cohorts (EORTC, MDACC, and MGH).

### Risk score

A risk score was calculated for each glioma patient using a linear combination of expression values of genes in the iCG signature^58^,^59^,^60^. The formula is shown below:


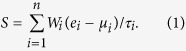


Here, *S* is the risk score of the patient; *n* is the number of genes in the iCG signature; *W*_*i*_ denotes the weight of gene *i* (as shown in Table 1), which indicates the direction of deregulation for gene *i* (1 or -1); *e*_*i*_ denotes the expression level of gene *i*; and *μ*_*i*_ and *τ*_*i*_ are the mean and standard deviation of the gene expression values for gene *i* across all samples, respectively. In each validation cohort, glioma patients were stratified into iCG^+^ and iCG^-^ groups with zero as the cutoff.

## Additional Information

**How to cite this article**: Wang, R. *et al.* Ion channel gene expression predicts survival in glioma patients. *Sci. Rep.* **5**, 11593; doi: 10.1038/srep11593 (2015).

## Supplementary Material

Supplementary Information

## Figures and Tables

**Table 1:** iCG signature.

Gene symbol	Gene description	Weight
*CACNA1D*	calcium channel, voltage-dependent, L type, alpha 1D subunit	−1
*CLCN6*	chloride channel, voltage-sensitive 6	−1
*CLIC1*	chloride intracellular channel 1	1
*CLIC4*	chloride intracellular channel 4	1
*GLRB*	glycine receptor, beta	−1
*GRIA2*	glutamate receptor, ionotropic, AMPA 2	−1
*GRID1*	glutamate receptor, ionotropic, delta 1	−1
*KCNAB1*	potassium voltage-gated channel, shaker-related subfamily, beta member 1	−1
*KCNB1*	potassium voltage-gated channel, Shab-related subfamily, member 1	−1
*KCND2*	potassium voltage-gated channel, Shal-related subfamily, member 2	−1
*KCNJ10*	potassium inwardly-rectifying channel, subfamily J, member 10	−1
*KCNMA1*	potassium large conductance calcium-activated channel, subfamily M, alpha member 1	−1
*KCNN3*	potassium intermediate/small conductance calcium-activated channel, subfamily N, member 3	−1
*KCNQ5*	potassium voltage-gated channel, KQT-like subfamily, member 5	−1
*NALCN*	sodium leak channel, non-selective	−1
*P2RX7*	purinergic receptor P2X, ligand-gated ion channel, 7	−1
*SCN1A*	sodium channel, voltage-gated, type I, alpha subunit	−1
*VDAC2*	voltage-dependent anion channel 2	−1

**Table 2:** Univariate Cox proportional hazards regression of survival by iCG status.

Cohort	*N*	HR	95% CI of HR	*P*-value
EORTC	95	2.84	(1.78, 4.54)	1.2 × 10^−5^
MDACC	77	2.51	(1.48, 4.25)	6.5 × 10^−4^
MGH	50	3.95	(1.90, 8.23)	2.4 × 10^−4^

Note – *N*: patient number; HR: hazard ratio; CI: confidence interval.

**Table 3:** Multivariate Cox proportional hazards regression of survival for the EORTC cohort (89 patients).

Covariate	HR	95% CI of HR	*P*-value
iCG + vs. -	3.26	(1.94, 5.50)	9.0 × 10^−6^
Age (per year)	1.01	(0.98, 1.04)	4.4 × 10^−1^
Gender male vs. female	0.72	(0.43, 1.21)	2.1 × 10^−1^
Type of surgery	0.69	(0.46, 1.03)	7.1 × 10^−2^
Performance status (0, 1, or 2)	1.57	(1.11, 2.22)	1.1 × 10^−2^
1p/19q LOH + vs. -	0.96	(0.46, 1.98)	9.0 × 10^−1^
Histology AOA vs. AOD	1.81	(1.02, 3.22)	4.2 × 10^−2^

Note – HR: hazard ratio; CI: confidence interval.

**Table 4:** Multivariate Cox proportional hazards regression of survival for the EORTC cohort (53 patients)

Covariate	HR	95% CI of HR	*P*-value
iCG + vs. -	3.35	(1.45, 7.70)	4.5 × 10^−3^
Age (per year)	1.04	(0.99, 1.10)	1.0 × 10^−1^
Gender male vs. female	1.07	(0.48, 2.37)	8.7 × 10^−1^
Type of surgery	0.46	(0.26, 0.83)	9.4 × 10^−3^
Performance status (0, 1, or 2)	1.49	(0.93, 2.40)	9.8 × 10^−2^
1p/19q LOH + vs. -	0.48	(0.13, 1.79)	2.8 × 10^−1^
*EGFR* amplification + vs. -	0.94	(0.41, 2.12)	8.8 × 10^−1^
*IDH1* mutation + vs. -	0.41	(0.17, 0.98)	4.6 × 10^−2^
*MGMT* methylation + vs. -	2.65	(0.89, 7.89)	7.9 × 10^−2^
Histology AOA vs. AOD	2.31	(1.05, 5.09)	3.8 × 10^−2^

Note – HR: hazard ratio; CI: confidence interval.

**Table 5:** Univariate Cox proportional hazards regression of survival against *KCNMA1* expression level.

Cohort	*N*	HR	95% CI of HR	*P*-value
EORTC	95	0.64	(0.45, 0.91)	1.3 × 10^−2^
MDACC	77	0.39	(0.20, 0.75)	4.8 × 10^−3^
MGH	50	0.11	(0.02, 0.68)	1.7 × 10^−2^

Note – *N*: patient number; HR: hazard ratio; CI: confidence interval.
