# Supplementary material for: Ion channel gene expression predicts survival in glioma patients
Source: Sci Rep. 2015 Aug 3;5:11593. doi: 10.1038/srep11593 (PMC4522676; doi:10.1038/srep11593)
Supplement: Supplementary Information [file srep11593-s1.pdf]

Supplementary information of "Ion channel gene expression predicts survival in glioma patients"

Rong Wang
Christopher I. Gurguis
Wanjun Gu
Eun A Ko
Inja Lim
Hyoweon Bang
Tong Zhou
Jae-Hong Ko*

March 20, 2015

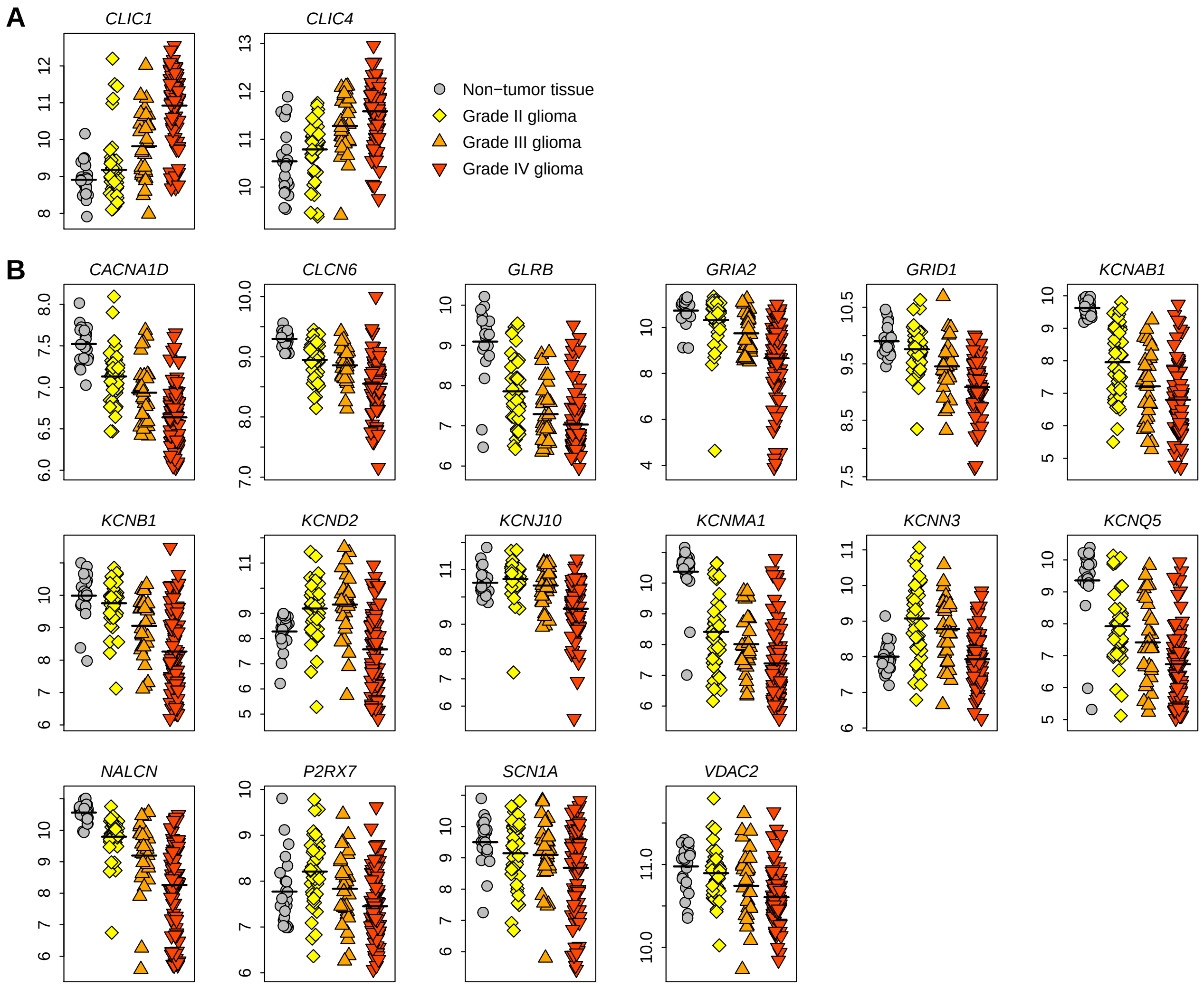

Supplementary Figure S1. Deregulated ion channel genes in glioma (HFH). The gene expression data were from the HFH cohort. (A) The two ion channel genes that are up-regulated in high-grade glioma. (B) The 16 ion channel genes that are down-regulated in high-grade glioma. For the gene with multiple probe sets, only the probe set with the most significant P-value was plotted. The horizontal black line indicates the mean of each category. Y-axis: $\log_2$-transformed expression values.

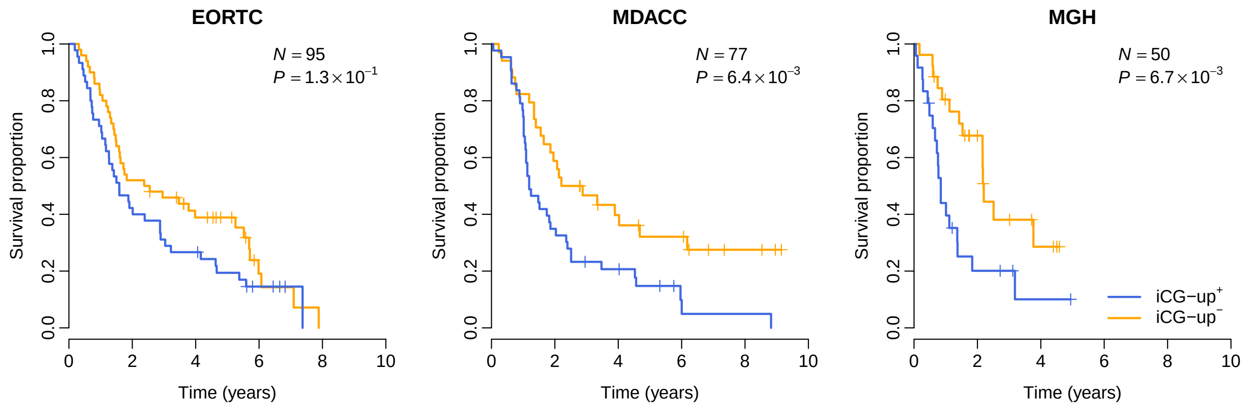

Supplementary Figure S2. Kaplan-Meier curves for glioma patients in the three validation cohorts (iCG-up). The expression of iCG-up predicts poor survival in the MDACC and MGH cohorts, but not in the EORTC cohort. The blue curves are for the iCG-up$^+$ patients while yellow curves are for the iCG-up$^-$ patients. P-values were calculated by log-rank test.

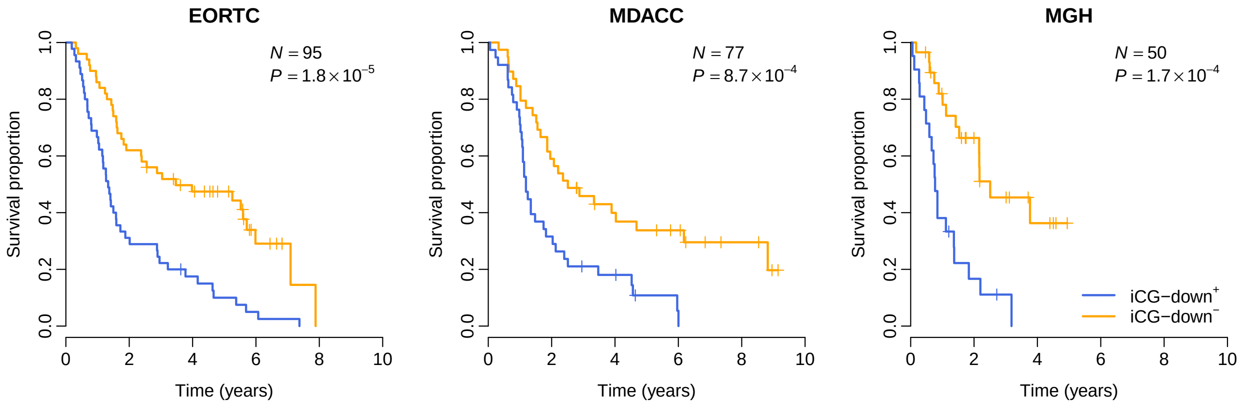

Supplementary Figure S3. Kaplan-Meier curves for glioma patients in the three validation cohorts (iCG-down). The expression of iCG-down predicts poor survival in the EORTC, MDACC, and MGH cohorts. The blue curves are for the iCG-down$^+$ patients while yellow curves are for the iCG-down$^-$ patients. P-values were calculated by log-rank test.

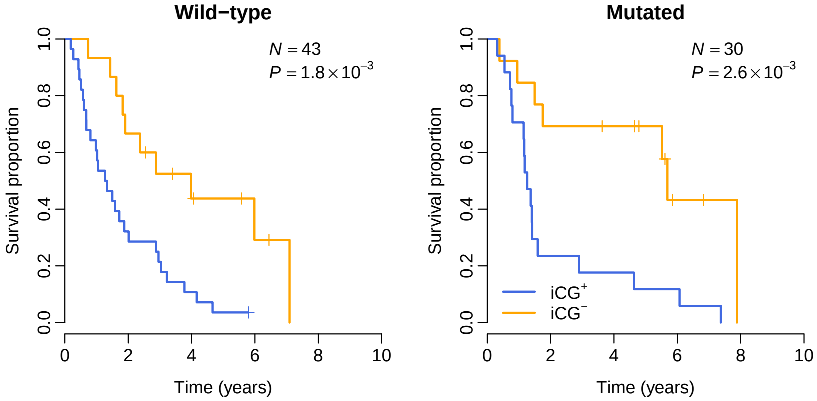

Supplementary Figure S4. Kaplan-Meier curves for glioma patients grouped by *IDH1* mutation status. The left panel is for the patients without *IDH1* mutation while the right panel is for the patients with mutated *IDH1*. The expression of iCG predicts poor survival in both the subgroups from the EORTC cohort. The blue curves are for the iCG$^+$ patients while yellow curves are for the iCG$^-$ patients. P-values were calculated by log-rank test.

Supplementary Table S1. Probe sets/ genes that are differentially expressed with glioma grade

Probeset ID	Gene symbol	Gene description	ρ	Adjusted *P*-value
205579_at	*HRH1*	histamine receptor H1	0.738	3.8E-04
1555575_a_at	*KDELR1*	KDEL (Lys-Asp-Glu-Leu) endoplasmic reticulum protein retention receptor 1	0.724	4.0E-04
227055_at	*METTL7B*	methyltransferase like 7B	0.720	4.3E-04
216331_at	*ITGA7*	integrin, alpha 7	0.714	4.7E-04
218257_s_at	*UGGT1*	UDP-glucose glycoprotein glucosyltransferase 1	0.689	6.3E-04
219118_at	*FKBP11*	FK506 binding protein 11, 19 kDa	0.677	8.0E-04
210427_x_at	*ANXA2*	annexin A2	0.677	8.0E-04
222986_s_at	*SHISA5*	shisa homolog 5 (Xenopus laevis)	0.673	8.7E-04
210058_at	*MAPK13*	mitogen-activated protein kinase 13	0.671	8.7E-04
202734_at	*TRIP10*	thyroid hormone receptor interactor 10	0.667	8.7E-04
37408_at	*MRC2*	mannose receptor, C type 2	0.666	8.8E-04
203570_at	*LOXL1*	lysyl oxidase-like 1	0.666	8.8E-04
214315_x_at	*CALR*	calreticulin	0.665	8.8E-04
1558856_at	*DMRTA2*	DMRT-like family A2	0.655	1.1E-03
201590_x_at	*ANXA2*	annexin A2	0.654	1.1E-03
34221_at	*HMGXB3*	HMG box domain containing 3	0.651	1.2E-03
63009_at	*SHQ1*	SHQ1 homolog (S. cerevisiae)	0.651	1.2E-03
217733_s_at	*TMSB10*	thymosin beta 10	0.648	1.2E-03
213503_x_at	*ANXA2*	annexin A2	0.648	1.2E-03
208782_at	*FSTL1*	follistatin-like 1	0.645	1.3E-03
216442_x_at	*FN1*	fibronectin 1	0.644	1.3E-03
203820_s_at	*IGF2BP3*	insulin-like growth factor 2 mRNA binding protein 3	0.642	1.3E-03
210495_x_at	*FN1*	fibronectin 1	0.640	1.4E-03
212464_s_at	*FN1*	fibronectin 1	0.639	1.4E-03
213693_s_at	*MUC1*	mucin 1, cell surface associated	0.639	1.4E-03
209395_at	*CHI3L1*	chitinase 3-like 1 (cartilage glycoprotein-39)	0.630	1.6E-03
224859_at	*CD276*	CD276 molecule	0.629	1.6E-03
211719_x_at	*FN1*	fibronectin 1	0.628	1.7E-03
208789_at	*PTRF*	polymerase I and transcript release factor	0.627	1.7E-03
200770_s_at	*LAMC1*	laminin, gamma 1 (formerly LAMB2)	0.626	1.7E-03
226060_at	*RFT1*	RFT1 homolog (S. cerevisiae)	0.624	1.8E-03
227628_at	*GPX8*	glutathione peroxidase 8 (putative)	0.623	1.8E-03
218961_s_at	*PNKP*	polynucleotide kinase 3'-phosphatase	0.623	1.8E-03
202403_s_at	*COL1A2*	collagen, type I, alpha 2	0.623	1.8E-03
217778_at	*SLC39A1*	solute carrier family 39 (zinc transporter), member 1	0.622	1.8E-03
204517_at	*PPIC*	peptidylprolyl isomerase C (cyclophilin C)	0.620	1.9E-03
215076_s_at	*COL3A1*	collagen, type III, alpha 1	0.620	1.9E-03
228033_at	*E2F7*	E2F transcription factor 7	0.617	2.0E-03
209626_s_at	*OSBPL3*	oxysterol binding protein-like 3	0.616	2.1E-03
203021_at	*SLPI*	secretory leukocyte peptidase inhibitor	0.615	2.1E-03
218795_at	*ACP6*	acid phosphatase 6, lysophosphatidic	0.615	2.1E-03
200916_at	*TAGLN2*	transgelin 2	0.614	2.1E-03
220750_s_at	*LEPRE1*	leucine proline-enriched proteoglycan (leprecan) 1	0.613	2.2E-03
209663_s_at	*ITGA7*	integrin, alpha 7	0.613	2.2E-03
203411_s_at	*LMNA*	lamin A/C	0.612	2.2E-03
218424_s_at	*STEAP3*	STEAP family member 3, metalloreductase	0.612	2.2E-03
235417_at	*SPOCD1*	SPOC domain containing 1	0.609	2.3E-03

Probeset ID	Gene symbol	Gene description	ρ	Adjusted *P*-value
203764_at	*DLGAP5*	discs, large (Drosophila) homolog-associated protein 5	0.609	2.3E-03
200791_s_at	*IQGAP1*	IQ motif containing GTPase activating protein 1	0.609	2.3E-03
203706_s_at	*FZD7*	frizzled family receptor 7	0.609	2.3E-03
211270_x_at	*PTBP1*	polypyrimidine tract binding protein 1	0.609	2.3E-03
241763_s_at	*FBXO32*	F-box protein 32	0.609	2.3E-03
226722_at	*FAM20C*	family with sequence similarity 20, member C	0.608	2.3E-03
229624_at	*OPA3*	optic atrophy 3 (autosomal recessive, with chorea and spastic paraplegia)	0.608	2.3E-03
211161_s_at	*COL3A1*	collagen, type III, alpha 1	0.608	2.3E-03
224911_s_at	*DCBLD2*	discoidin, CUB and LCCL domain containing 2	0.608	2.3E-03
201852_x_at	*COL3A1*	collagen, type III, alpha 1	0.606	2.4E-03
225032_at	*FNDC3B*	fibronectin type III domain containing 3B	0.606	2.4E-03
202605_at	*GUSB*	glucuronidase, beta	0.606	2.4E-03
202404_s_at	*COL1A2*	collagen, type I, alpha 2	0.606	2.4E-03
243283_at	*CROT*	carnitine O-octanoyltransferase	0.606	2.4E-03
218959_at	*HOXC10*	homeobox C10	0.605	2.5E-03
236249_at	*IKBIP*	IKBKB interacting protein	0.602	2.6E-03
207714_s_at	*SERPINH1*	serpin peptidase inhibitor, clade H (heat shock protein 47), member 1, (collagen binding protein 1)	0.601	2.7E-03
45297_at	*EHD2*	EH-domain containing 2	0.600	2.7E-03
203705_s_at	*FZD7*	frizzled family receptor 7	0.600	2.7E-03
200999_s_at	*CKAP4*	cytoskeleton-associated protein 4	0.598	2.8E-03
209396_s_at	*CHI3L1*	chitinase 3-like 1 (cartilage glycoprotein-39)	0.597	2.8E-03
208658_at	*PDIA4*	protein disulfide isomerase family A, member 4	0.596	2.8E-03
209215_at	*MFSD10*	major facilitator superfamily domain containing 10	0.595	2.9E-03
218115_at	*ASF1B*	ASF1 anti-silencing function 1 homolog B (S. cerevisiae)	0.595	2.9E-03
213811_x_at	*TCF3*	transcription factor 3 (E2A immunoglobulin enhancer binding factors E12/E47)	0.595	2.9E-03
215199_at	*CALD1*	caldesmon 1	0.593	3.0E-03
201438_at	*COL6A3*	collagen, type VI, alpha 3	0.592	3.0E-03
218399_s_at	*CDCA4*	cell division cycle associated 4	0.592	3.0E-03
202238_s_at	*NNMT*	nicotinamide N-methyltransferase	0.591	3.0E-03
214853_s_at	*SHC1*	SHC (Src homology 2 domain containing) transforming protein 1	0.591	3.1E-03
203921_at	*CHST2*	carbohydrate (N-acetylglucosamine-6-O) sulfotransferase 2	0.590	3.1E-03
209156_s_at	*COL6A2*	collagen, type VI, alpha 2	0.590	3.1E-03
202189_x_at	*PTBP1*	polypyrimidine tract binding protein 1	0.590	3.1E-03
222206_s_at	*NCLN*	nicalin	0.587	3.3E-03
236028_at	*IBSP*	integrin-binding sialoprotein	0.587	3.3E-03
202910_s_at	*CD97*	CD97 molecule	0.587	3.3E-03
206157_at	*PTX3*	pentraxin 3, long	0.586	3.3E-03
204549_at	*IKBKE*	inhibitor of kappa light polypeptide gene enhancer in B-cells, kinase epsilon	0.585	3.4E-03
218460_at	*HEATR2*	HEAT repeat containing 2	0.584	3.5E-03
203857_s_at	*PDIA5*	protein disulfide isomerase family A, member 5	0.583	3.6E-03
1557938_s_at	*PTRF*	polymerase I and transcript release factor	0.583	3.6E-03
208637_x_at	*ACTN1*	actinin, alpha 1	0.583	3.6E-03
206201_s_at	*MEOX2*	mesenchyme homeobox 2	0.583	3.6E-03
215867_x_at	*CA12*	carbonic anhydrase XII	0.582	3.7E-03

Probeset ID	Gene symbol	Gene description	ρ	Adjusted *P*-value
204306_s_at	*CD151*	CD151 molecule (Raph blood group)	0.581	3.8E-03
204256_at	*ELOVL6*	ELOVL fatty acid elongase 6	0.581	3.8E-03
201744_s_at	*LUM*	lumican	0.580	3.9E-03
214164_x_at	*CA12*	carbonic anhydrase XII	0.580	3.9E-03
200663_at	*CD63*	CD63 molecule	0.579	3.9E-03
203963_at	*CA12*	carbonic anhydrase XII	0.579	3.9E-03
203819_s_at	*IGF2BP3*	insulin-like growth factor 2 mRNA binding protein 3	0.578	4.0E-03
223274_at	*TCF19*	transcription factor 19	0.578	4.1E-03
228141_at	*GPX8*	glutathione peroxidase 8 (putative)	0.577	4.1E-03
212109_at	*HN1L*	hematological and neurological expressed 1-like	0.577	4.1E-03
202237_at	*NNMT*	nicotinamide N-methyltransferase	0.575	4.3E-03
241762_at	*FBXO32*	F-box protein 32	0.575	4.3E-03
201664_at	*SMC4*	structural maintenance of chromosomes 4	0.574	4.3E-03
201749_at	*ECE1*	endothelin converting enzyme 1	0.574	4.3E-03
219990_at	*E2F8*	E2F transcription factor 8	0.573	4.4E-03
236285_at	*KLHDC7B*	kelch domain containing 7B	0.572	4.5E-03
201012_at	*ANXA1*	annexin A1	0.572	4.5E-03
227870_at	*IGDCC4*	immunoglobulin superfamily, DCC subclass, member 4	0.572	4.5E-03
218473_s_at	*GLT25D1*	glycosyltransferase 25 domain containing 1	0.571	4.5E-03
203975_s_at	*CHAF1A*	chromatin assembly factor 1, subunit A (p150)	0.570	4.6E-03
225415_at	*DTX3L*	deltex 3-like (Drosophila)	0.570	4.6E-03
227314_at	*ITGA2*	integrin, alpha 2 (CD49B, alpha 2 subunit of VLA-2 receptor)	0.569	4.7E-03
215049_x_at	*CD163*	CD163 molecule	0.569	4.8E-03
218542_at	*CEP55*	centrosomal protein 55kDa	0.569	4.8E-03
219386_s_at	*SLAMF8*	SLAM family member 8	0.569	4.8E-03
210735_s_at	*CA12*	carbonic anhydrase XII	0.569	4.8E-03
201324_at	*EMP1*	epithelial membrane protein 1	0.568	4.8E-03
204659_s_at	*GFER*	growth factor, augmenter of liver regeneration	0.567	4.9E-03
227239_at	*FAM126A*	family with sequence similarity 126, member A	0.567	4.9E-03
1552309_a_at	*NEXN*	nexilin (F actin binding protein)	0.566	5.0E-03
208688_x_at	*EIF3B*	eukaryotic translation initiation factor 3, subunit B	0.566	5.0E-03
236278_at	*HIST1H3E*	Histone cluster 1, H3e	0.566	5.0E-03
227877_at	*ANXA2R*	annexin A2 receptor	0.565	5.0E-03
204017_at	*KDELR3*	KDEL (Lys-Asp-Glu-Leu) endoplasmic reticulum protein retention receptor 3	0.565	5.1E-03
212077_at	*CALD1*	caldesmon 1	0.564	5.1E-03
226777_at	*ADAM12*	ADAM metallopeptidase domain 12	0.564	5.1E-03
203645_s_at	*CD163*	CD163 molecule	0.564	5.1E-03
204573_at	*CROT*	carnitine O-octanoyltransferase	0.563	5.2E-03
222693_at	*FNDC3B*	fibronectin type III domain containing 3B	0.563	5.2E-03
204150_at	*STAB1*	stabilin 1	0.563	5.2E-03
217738_at	*NAMPT*	nicotinamide phosphoribosyltransferase	0.563	5.2E-03
244710_at	*LRGUK*	leucine-rich repeats and guanylate kinase domain containing	0.561	5.5E-03
229802_at	*WISP1*	WNT1 inducible signaling pathway protein 1	0.561	5.5E-03
204508_s_at	*CA12*	carbonic anhydrase XII	0.561	5.5E-03
212922_s_at	*SMYD2*	SET and MYND domain containing 2	0.560	5.5E-03
201105_at	*LGALS1*	lectin, galactoside-binding, soluble, 1	0.560	5.5E-03

Probeset ID	Gene symbol	Gene description	ρ	Adjusted *P*-value
218002_s_at	*CXCL14*	chemokine (C-X-C motif) ligand 14	0.559	5.6E-03
217739_s_at	*NAMPT*	nicotinamide phosphoribosyltransferase	0.559	5.6E-03
221729_at	*COL5A2*	collagen, type V, alpha 2	0.557	5.8E-03
201389_at	*ITGA5*	integrin, alpha 5 (fibronectin receptor, alpha polypeptide)	0.556	5.9E-03
204932_at	*TNFRSF11B*	tumor necrosis factor receptor superfamily, member 11b	0.556	6.0E-03
202668_at	*EFNB2*	ephrin-B2	0.555	6.1E-03
213338_at	*TMEM158*	transmembrane protein 158 (gene/pseudogene)	0.554	6.1E-03
217770_at	*PIGT*	phosphatidylinositol glycan anchor biosynthesis, class T	0.554	6.2E-03
223241_at	*SNX8*	sorting nexin 8	0.554	6.2E-03
208074_s_at	*AP2S1*	adaptor-related protein complex 2, sigma 1 subunit	0.553	6.3E-03
218070_s_at	*GMPPA*	GDP-mannose pyrophosphorylase A	0.553	6.3E-03
218662_s_at	*NCAPG*	non-SMC condensin I complex, subunit G	0.552	6.3E-03
212792_at	*DPY19L1*	dpy-19-like 1 (C. elegans)	0.552	6.3E-03
218802_at	*CCDC109B*	coiled-coil domain containing 109B	0.552	6.4E-03
1554600_s_at	*LMNA*	lamin A/C	0.552	6.4E-03
203282_at	*GBE1*	glucan (1,4-alpha-), branching enzyme 1	0.552	6.4E-03
222692_s_at	*FNDC3B*	fibronectin type III domain containing 3B	0.551	6.5E-03
222411_s_at	*SSR3*	signal sequence receptor, gamma (translocon-associated protein gamma)	0.550	6.5E-03
211964_at	*COL4A2*	collagen, type IV, alpha 2	0.550	6.6E-03
204646_at	*DPYD*	dihydropyrimidine dehydrogenase	0.549	6.6E-03
221999_at	*VRK3*	vaccinia related kinase 3	0.549	6.6E-03
218833_at	*ZAK*	sterile alpha motif and leucine zipper containing kinase AZK	0.549	6.7E-03
227998_at	*S100A16*	S100 calcium binding protein A16	0.549	6.7E-03
243750_x_at	*FAM207A*	family with sequence similarity 207, member A	0.549	6.7E-03
225803_at	*FBXO32*	F-box protein 32	0.548	6.7E-03
226032_at	*CASP2*	caspase 2, apoptosis-related cysteine peptidase	0.548	6.7E-03
200839_s_at	*CTSB*	cathepsin B	0.548	6.8E-03
223363_at	*PSMG3*	proteasome (prosome, macropain) assembly chaperone 3	0.548	6.8E-03
229400_at	*HOXD10*	homeobox D10	0.548	6.8E-03
219278_at	*MAP3K6*	mitogen-activated protein kinase kinase kinase 6	0.547	6.9E-03
224715_at	*WDR34*	WD repeat domain 34	0.547	6.9E-03
202718_at	*IGFBP2*	insulin-like growth factor binding protein 2, 36kDa	0.546	7.0E-03
210978_s_at	*TAGLN2*	transgelin 2	0.546	7.0E-03
212082_s_at	*MYL6*	myosin, light chain 6, alkali, smooth muscle and non-muscle	0.546	7.0E-03
208636_at	*ACTN1*	actinin, alpha 1	0.546	7.0E-03
225636_at	*STAT2*	signal transducer and activator of transcription 2, 113kDa	0.545	7.1E-03
210776_x_at	*TCF3*	transcription factor 3 (E2A immunoglobulin enhancer binding factors E12/E47)	0.545	7.1E-03
200757_s_at	*CALU*	calumenin	0.545	7.1E-03
218066_at	*SLC12A7*	solute carrier family 12 (potassium/chloride transporters), member 7	0.545	7.1E-03
211047_x_at	*AP2S1*	adaptor-related protein complex 2, sigma 1 subunit	0.545	7.1E-03
229538_s_at	*IQGAP3*	IQ motif containing GTPase activating protein 3	0.544	7.2E-03
224511_s_at	*TXNDC17*	thioredoxin domain containing 17	0.543	7.3E-03
228069_at	*FAM54A*	family with sequence similarity 54, member A	0.543	7.3E-03
226282_at	*PTPN14*	protein tyrosine phosphatase, non-receptor type 14	0.543	7.4E-03

Probeset ID	Gene symbol	Gene description	ρ	Adjusted *P*-value
209124_at	*MYD88*	myeloid differentiation primary response gene (88)	0.541	7.6E-03
201817_at	*UBE3C*	ubiquitin protein ligase E3C	0.541	7.7E-03
218880_at	*FOSL2*	FOS-like antigen 2	0.540	7.7E-03
215706_x_at	*ZYX*	zyxin	0.540	7.7E-03
227807_at	*PARP9*	poly (ADP-ribose) polymerase family, member 9	0.540	7.7E-03
209263_x_at	*TSPAN4*	tetraspanin 4	0.539	7.8E-03
202952_s_at	*ADAM12*	ADAM metallopeptidase domain 12	0.539	7.8E-03
225321_s_at	*PILRB*	paired immunoglobin-like type 2 receptor beta	0.539	7.8E-03
204083_s_at	*TPM2*	tropomyosin 2 (beta)	0.539	7.8E-03
200629_at	*WARS*	tryptophanyl-tRNA synthetase	0.538	8.0E-03
230022_at	*CLEC18A*	C-type lectin domain family 18, member A	0.538	8.0E-03
204451_at	*FZD1*	frizzled family receptor 1	0.538	8.0E-03
204352_at	*TRAF5*	TNF receptor-associated factor 5	0.538	8.0E-03
212988_x_at	*ACTG1*	actin, gamma 1	0.538	8.0E-03
209773_s_at	*RRM2*	ribonucleotide reductase M2	0.538	8.0E-03
218950_at	*ARAP3*	ArfGAP with RhoGAP domain, ankyrin repeat and PH domain 3	0.538	8.0E-03
202596_at	*ENSA*	endosulfine alpha	0.538	8.0E-03
230036_at	*SAMD9L*	sterile alpha motif domain containing 9-like	0.537	8.0E-03
205909_at	*POLE2*	polymerase (DNA directed), epsilon 2, accessory subunit	0.537	8.0E-03
203186_s_at	*S100A4*	S100 calcium binding protein A4	0.537	8.0E-03
208790_s_at	*PTRF*	polymerase I and transcript release factor	0.537	8.1E-03
222866_s_at	*FLVCR2*	feline leukemia virus subgroup C cellular receptor family, member 2	0.537	8.1E-03
222046_at	*SRRT*	serrate RNA effector molecule homolog (Arabidopsis)	0.536	8.2E-03
205265_s_at	*SPEG*	SPEG complex locus	0.536	8.2E-03
201941_at	*CPD*	carboxypeptidase D	0.536	8.3E-03
221730_at	*COL5A2*	collagen, type V, alpha 2	0.535	8.3E-03
241812_at	*SPATS2L*	spermatogenesis associated, serine-rich 2-like	0.535	8.3E-03
211126_s_at	*CSRP2*	cysteine and glycine-rich protein 2	0.534	8.6E-03
210220_at	*FZD2*	frizzled family receptor 2	0.534	8.6E-03
202381_at	*ADAM9*	ADAM metallopeptidase domain 9	0.534	8.6E-03
211980_at	*COL4A1*	collagen, type IV, alpha 1	0.533	8.6E-03
202095_s_at	*BIRC5*	baculoviral IAP repeat containing 5	0.533	8.6E-03
209651_at	*TGFB1I1*	transforming growth factor beta 1 induced transcript 1	0.533	8.7E-03
226603_at	*SAMD9L*	sterile alpha motif domain containing 9-like	0.533	8.7E-03
38487_at	*STAB1*	stabilin 1	0.533	8.7E-03
203968_s_at	*CDC6*	cell division cycle 6 homolog (S. cerevisiae)	0.533	8.7E-03
203484_at	*SEC61G*	Sec61 gamma subunit	0.532	8.9E-03
210609_s_at	*TP53I3*	tumor protein p53 inducible protein 3	0.531	8.9E-03
217899_at	*TMEM214*	transmembrane protein 214	0.531	8.9E-03
203729_at	*EMP3*	epithelial membrane protein 3	0.531	8.9E-03
215198_s_at	*CALD1*	caldesmon 1	0.531	8.9E-03
206026_s_at	*TNFAIP6*	tumor necrosis factor, alpha-induced protein 6	0.530	9.2E-03
200771_at	*LAMC1*	laminin, gamma 1 (formerly LAMB2)	0.529	9.4E-03
207847_s_at	*MUC1*	mucin 1, cell surface associated	0.528	9.5E-03
218728_s_at	*CNIH4*	cornichon homolog 4 (Drosophila)	0.528	9.5E-03
212115_at	*HN1L*	hematological and neurological expressed 1-like	0.528	9.6E-03
229304_s_at	*MLF1IP*	MLF1 interacting protein	0.526	9.8E-03

Probeset ID	Gene symbol	Gene description	ρ	Adjusted *P*-value
223077_at	*TMOD3*	tropomodulin 3 (ubiquitous)	0.526	9.8E-03
210840_s_at	*IQGAP1*	IQ motif containing GTPase activating protein 1	0.526	9.9E-03
223690_at	*LTBP2*	latent transforming growth factor beta binding protein 2	0.526	9.9E-03
206025_s_at	*TNFAIP6*	tumor necrosis factor, alpha-induced protein 6	0.525	1.0E-02
200808_s_at	*ZYX*	zyxin	0.525	1.0E-02
204908_s_at	*BCL3*	B-cell CLL/lymphoma 3	0.523	1.0E-02
223419_at	*FBXW9*	F-box and WD repeat domain containing 9	0.523	1.0E-02
244427_at	*KIF23*	Kinesin family member 23	0.523	1.0E-02
202389_s_at	*HTT*	huntingtin	0.523	1.1E-02
217867_x_at	*BACE2*	beta-site APP-cleaving enzyme 2	0.522	1.1E-02
1553983_at	*DTYMK*	deoxythymidylate kinase (thymidylate kinase)	0.522	1.1E-02
208161_s_at	*ABCC3*	ATP-binding cassette, sub-family C (CFTR/MRP), member 3	0.522	1.1E-02
202185_at	*PLOD3*	procollagen-lysine, 2-oxoglutarate 5-dioxygenase 3	0.521	1.1E-02
202107_s_at	*MCM2*	minichromosome maintenance complex component 2	0.521	1.1E-02
225328_at	*FBXO32*	F-box protein 32	0.521	1.1E-02
207165_at	*HMMR*	hyaluronan-mediated motility receptor (RHAMM)	0.520	1.1E-02
225285_at	*BCAT1*	branched chain amino-acid transaminase 1, cytosolic	0.520	1.1E-02
209627_s_at	*OSBPL3*	oxysterol binding protein-like 3	0.520	1.1E-02
210001_s_at	*SOCS1*	suppressor of cytokine signaling 1	0.520	1.1E-02
213730_x_at	*TCF3*	transcription factor 3 (E2A immunoglobulin enhancer binding factors E12/E47)	0.520	1.1E-02
221587_s_at	*C19orf24*	chromosome 19 open reading frame 24	0.519	1.1E-02
223361_at	*ABRACL*	ABRA C-terminal like	0.519	1.1E-02
205266_at	*LIF*	leukemia inhibitory factor	0.519	1.1E-02
38158_at	*ESPL1*	extra spindle pole bodies homolog 1 (S. cerevisiae)	0.519	1.1E-02
200755_s_at	*CALU*	calumenin	0.519	1.1E-02
221710_x_at	*FAM176B*	family with sequence similarity 176, member B	0.519	1.1E-02
201505_at	*LAMB1*	laminin, beta 1	0.519	1.1E-02
219923_at	*TRIM45*	tripartite motif containing 45	0.518	1.1E-02
212212_s_at	*INTS1*	integrator complex subunit 1	0.518	1.1E-02
206364_at	*KIF14*	kinesin family member 14	0.518	1.1E-02
212971_at	*CARS*	cysteinyl-tRNA synthetase	0.518	1.1E-02
217967_s_at	*FAM129A*	family with sequence similarity 129, member A	0.518	1.1E-02
41660_at	*CELSR1*	cadherin, EGF LAG seven-pass G-type receptor 1 (flamingo homolog, Drosophila)	0.518	1.1E-02
211271_x_at	*PTBP1*	polypyrimidine tract binding protein 1	0.517	1.2E-02
202503_s_at	*KIAA0101*	KIAA0101	0.517	1.2E-02
226103_at	*NEXN*	nexilin (F actin binding protein)	0.517	1.2E-02
222871_at	*KLHDC8A*	kelch domain containing 8A	0.517	1.2E-02
204682_at	*LTBP2*	latent transforming growth factor beta binding protein 2	0.516	1.2E-02
207030_s_at	*CSRP2*	cysteine and glycine-rich protein 2	0.516	1.2E-02
55583_at	*DOCK6*	dedicator of cytokinesis 6	0.516	1.2E-02
226416_at	*ERI1*	exoribonuclease 1	0.515	1.2E-02
204608_at	*ASL*	argininosuccinate lyase	0.515	1.2E-02
202175_at	*CHPF*	chondroitin polymerizing factor	0.515	1.2E-02
215438_x_at	*GSPT1*	G1 to S phase transition 1	0.515	1.2E-02
201325_s_at	*EMP1*	epithelial membrane protein 1	0.515	1.2E-02
226113_at	*ZNF436*	zinc finger protein 436	0.515	1.2E-02

Probeset ID	Gene symbol	Gene description	ρ	Adjusted *P*-value
222581_at	*XPR1*	xenotropic and polytropic retrovirus receptor 1	0.514	1.2E-02
223376_s_at	*BRI3*	brain protein I3	0.514	1.2E-02
200660_at	*S100A11*	S100 calcium binding protein A11	0.514	1.2E-02
38703_at	*DNPEP*	aspartyl aminopeptidase	0.513	1.2E-02
205339_at	*STIL*	SCL/TAL1 interrupting locus	0.513	1.3E-02
208079_s_at	*AURKA*	aurora kinase A	0.513	1.3E-02
211966_at	*COL4A2*	collagen, type IV, alpha 2	0.512	1.3E-02
229350_x_at	*PARP10*	poly (ADP-ribose) polymerase family, member 10	0.512	1.3E-02
218585_s_at	*DTL*	denticleless E3 ubiquitin protein ligase homolog (Drosophila)	0.512	1.3E-02
222484_s_at	*CXCL14*	chemokine (C-X-C motif) ligand 14	0.512	1.3E-02
206858_s_at	*HOXC6*	homeobox C6	0.512	1.3E-02
225867_at	*VASN*	vasorin	0.511	1.3E-02
225806_at	*AJUBA*	ajuba LIM protein	0.510	1.3E-02
202430_s_at	*PLSCR1*	phospholipid scramblase 1	0.510	1.3E-02
202755_s_at	*GPC1*	glypican 1	0.510	1.3E-02
203201_at	*PMM2*	phosphomannomutase 2	0.510	1.3E-02
204844_at	*ENPEP*	glutamyl aminopeptidase (aminopeptidase A)	0.510	1.3E-02
226661_at	*CDCA2*	cell division cycle associated 2	0.509	1.3E-02
229247_at	*FBLN7*	fibulin 7	0.509	1.3E-02
205539_at	*AVIL*	advillin	0.509	1.3E-02
219493_at	*SHCBP1*	SHC SH2-domain binding protein 1	0.509	1.3E-02
205059_s_at	*IDUA*	iduronidase, alpha-L-	0.509	1.3E-02
206688_s_at	*CPSF4*	cleavage and polyadenylation specific factor 4, 30kDa	0.509	1.3E-02
202276_at	*SHFM1*	split hand/foot malformation (ectrodactyly) type 1	0.509	1.3E-02
213214_x_at	*ACTG1*	actin, gamma 1	0.509	1.3E-02
223993_s_at	*CNIH4*	cornichon homolog 4 (Drosophila)	0.509	1.3E-02
205199_at	*CA9*	carbonic anhydrase IX	0.508	1.3E-02
226552_at	*IER5L*	immediate early response 5-like	0.508	1.4E-02
1568634_a_at	*LRRC66*	leucine rich repeat containing 66	0.508	1.4E-02
200826_at	*SNRPD2*	small nuclear ribonucleoprotein D2 polypeptide 16.5kDa	0.508	1.4E-02
226517_at	*BCAT1*	branched chain amino-acid transaminase 1, cytosolic	0.507	1.4E-02
1558508_a_at	*C1orf53*	chromosome 1 open reading frame 53	0.507	1.4E-02
224374_s_at	*EMILIN2*	elastin microfibril interfacer 2	0.506	1.4E-02
226296_s_at	*MRPS15*	mitochondrial ribosomal protein S15	0.506	1.4E-02
222446_s_at	*BACE2*	beta-site APP-cleaving enzyme 2	0.506	1.4E-02
221708_s_at	*UNC45A*	unc-45 homolog A (C. elegans)	0.506	1.4E-02
204702_s_at	*NFE2L3*	nuclear factor (erythroid-derived 2)-like 3	0.506	1.4E-02
222047_s_at	*SRRT*	serrate RNA effector molecule homolog (Arabidopsis)	0.506	1.4E-02
227325_at	*PRR24*	proline rich 24	0.505	1.4E-02
221024_s_at	*SLC2A10*	solute carrier family 2 (facilitated glucose transporter), member 10	0.505	1.4E-02
218305_at	*IPO4*	importin 4	0.505	1.4E-02
213373_s_at	*CASP8*	caspase 8, apoptosis-related cysteine peptidase	0.505	1.4E-02
213428_s_at	*COL6A1*	collagen, type VI, alpha 1	0.504	1.5E-02
201920_at	*SLC20A1*	solute carrier family 20 (phosphate transporter), member 1	0.504	1.5E-02
207157_s_at	*GNG5*	guanine nucleotide binding protein (G protein), gamma 5	0.504	1.5E-02
213226_at	*CCNA2*	cyclin A2	0.503	1.5E-02

Probeset ID	Gene symbol	Gene description	ρ	Adjusted *P*-value
202733_at	*P4HA2*	prolyl 4-hydroxylase, alpha polypeptide II	0.503	1.5E-02
55093_at	*CHPF2*	chondroitin polymerizing factor 2	0.503	1.5E-02
212235_at	*PLXND1*	plexin D1	0.503	1.5E-02
244533_at	*PTPN14*	protein tyrosine phosphatase, non-receptor type 14	0.503	1.5E-02
235545_at	*DEPDC1*	DEP domain containing 1	0.503	1.5E-02
201943_s_at	*CPD*	carboxypeptidase D	0.503	1.5E-02
219025_at	*CD248*	CD248 molecule, endosialin	0.503	1.5E-02
204483_at	*ENO3*	enolase 3 (beta, muscle)	0.502	1.5E-02
1559946_s_at	*RUVBL2*	RuvB-like 2 (E. coli)	0.502	1.5E-02
209714_s_at	*CDKN3*	cyclin-dependent kinase inhibitor 3	0.502	1.5E-02
223923_at	*C21orf62*	chromosome 21 open reading frame 62	0.502	1.5E-02
211250_s_at	*SH3BP2*	SH3-domain binding protein 2	0.502	1.5E-02
209803_s_at	*PHLDA2*	pleckstrin homology-like domain, family A, member 2	0.502	1.5E-02
224699_s_at	*ESYT2*	extended synaptotagmin-like protein 2	0.502	1.5E-02
228776_at	*GJC1*	gap junction protein, gamma 1, 45kDa	0.502	1.5E-02
230147_at	*F2RL2*	coagulation factor II (thrombin) receptor-like 2	0.502	1.5E-02
231876_at	*TRIM56*	tripartite motif containing 56	0.502	1.5E-02
223995_at	*SLC12A9*	solute carrier family 12 (potassium/chloride transporters), member 9	0.501	1.5E-02
224097_s_at	*F11R*	F11 receptor	0.501	1.5E-02
215760_s_at	*SBNO2*	strawberry notch homolog 2 (Drosophila)	0.501	1.5E-02
44654_at	*G6PC3*	glucose 6 phosphatase, catalytic, 3	0.501	1.5E-02
209454_s_at	*TEAD3*	TEA domain family member 3	0.500	1.6E-02
202627_s_at	*SERPINE1*	serpin peptidase inhibitor, clade E (nexin, plasminogen activator inhibitor type 1), member 1	0.499	1.6E-02
201160_s_at	*CSDA*	cold shock domain protein A	0.499	1.6E-02
211651_s_at	*LAMB1*	laminin, beta 1	0.499	1.6E-02
218982_s_at	*MRPS17*	mitochondrial ribosomal protein S17	0.498	1.6E-02
201680_x_at	*SRRT*	serrate RNA effector molecule homolog (Arabidopsis)	0.498	1.6E-02
203474_at	*IQGAP2*	IQ motif containing GTPase activating protein 2	0.498	1.6E-02
43977_at	*TMEM161A*	transmembrane protein 161A	0.498	1.6E-02
219773_at	*NOX4*	NADPH oxidase 4	0.498	1.6E-02
205812_s_at	*TMED9*	transmembrane emp24 protein transport domain containing 9	0.498	1.6E-02
213523_at	*CCNE1*	cyclin E1	0.498	1.6E-02
217750_s_at	*UBE2Z*	ubiquitin-conjugating enzyme E2Z	0.497	1.6E-02
200838_at	*CTSB*	cathepsin B	0.497	1.6E-02
203560_at	*GGH*	gamma-glutamyl hydrolase (conjugase, folylpolygammaglutamyl hydrolase)	0.497	1.6E-02
203164_at	*SLC33A1*	solute carrier family 33 (acetyl-CoA transporter), member 1	0.497	1.6E-02
223222_at	*SLC25A19*	solute carrier family 25 (mitochondrial thiamine pyrophosphate carrier), member 19	0.497	1.6E-02
210183_x_at	*PNN*	pinin, desmosome associated protein	0.497	1.6E-02
222757_s_at	*ZAK*	sterile alpha motif and leucine zipper containing kinase AZK	0.497	1.6E-02
210095_s_at	*IGFBP3*	insulin-like growth factor binding protein 3	0.496	1.7E-02
211429_s_at	*SERPINA1*	serpin peptidase inhibitor, clade A (alpha-1 antiproteinase, antitrypsin), member 1	0.496	1.7E-02
203576_at	*BCAT2*	branched chain amino-acid transaminase 2, mitochondrial	0.496	1.7E-02

Probeset ID	Gene symbol	Gene description	ρ	Adjusted *P*-value
212086_x_at	*LMNA*	lamin A/C	0.496	1.7E-02
202120_x_at	*AP2S1*	adaptor-related protein complex 2, sigma 1 subunit	0.496	1.7E-02
210150_s_at	*LAMA5*	laminin, alpha 5	0.496	1.7E-02
201110_s_at	*THBS1*	thrombospondin 1	0.496	1.7E-02
244654_at	*MYO1G*	myosin IG	0.495	1.7E-02
1562415_a_at	*SPOCD1*	SPOC domain containing 1	0.495	1.7E-02
213418_at	*HSPA6*	heat shock 70kDa protein 6 (HSP70B')	0.495	1.7E-02
211713_x_at	*KIAA0101*	KIAA0101	0.495	1.7E-02
225605_at	*TP53I13*	tumor protein p53 inducible protein 13	0.494	1.7E-02
202016_at	*MEST*	mesoderm specific transcript homolog (mouse)	0.494	1.7E-02
208815_x_at	*HSPA4*	heat shock 70kDa protein 4	0.493	1.8E-02
201422_at	*IFI30*	interferon, gamma-inducible protein 30	0.493	1.8E-02
217966_s_at	*FAM129A*	family with sequence similarity 129, member A	0.493	1.8E-02
213139_at	*SNAI2*	snail homolog 2 (Drosophila)	0.493	1.8E-02
38671_at	*PLXND1*	plexin D1	0.492	1.8E-02
201069_at	*MMP2*	matrix metallopeptidase 2 (gelatinase A, 72kDa gelatinase, 72kDa type IV collagenase)	0.492	1.8E-02
201890_at	*RRM2*	ribonucleotide reductase M2	0.492	1.8E-02
1558693_s_at	*C1orf85*	chromosome 1 open reading frame 85	0.492	1.8E-02
202870_s_at	*CDC20*	cell division cycle 20 homolog (S. cerevisiae)	0.492	1.8E-02
211668_s_at	*PLAU*	plasminogen activator, urokinase	0.492	1.8E-02
222154_s_at	*SPATS2L*	spermatogenesis associated, serine-rich 2-like	0.492	1.8E-02
216306_x_at	*PTBP1*	polypyrimidine tract binding protein 1	0.491	1.8E-02
203046_s_at	*TIMELESS*	timeless homolog (Drosophila)	0.491	1.8E-02
227013_at	*LATS2*	LATS, large tumor suppressor, homolog 2 (Drosophila)	0.491	1.8E-02
217716_s_at	*SEC61A1*	Sec61 alpha 1 subunit (S. cerevisiae)	0.491	1.8E-02
200654_at	*P4HB*	prolyl 4-hydroxylase, beta polypeptide	0.491	1.8E-02
1558511_s_at	*ESYT2*	extended synaptotagmin-like protein 2	0.491	1.8E-02
201584_s_at	*DDX39A*	DEAD (Asp-Glu-Ala-Asp) box polypeptide 39A	0.491	1.8E-02
200666_s_at	*DNAJB1*	DnaJ (Hsp40) homolog, subfamily B, member 1	0.490	1.8E-02
206540_at	*GLB1L*	galactosidase, beta 1-like	0.490	1.8E-02
209129_at	*TRIP6*	thyroid hormone receptor interactor 6	0.490	1.8E-02
209191_at	*TUBB6*	tubulin, beta 6 class V	0.489	1.9E-02
211343_s_at	*COL13A1*	collagen, type XIII, alpha 1	0.489	1.9E-02
210187_at	*FKBP1A*	FK506 binding protein 1A, 12kDa	0.489	1.9E-02
205381_at	*LRRC17*	leucine rich repeat containing 17	0.489	1.9E-02
239930_at	*GALNT2*	UDP-N-acetyl-alpha-D-galactosamine:polypeptide N-acetylgalactosaminyltransferase 2 (GalNAc-T2)	0.489	1.9E-02
209172_s_at	*CENPF*	centromere protein F, 350/400kDa (mitosin)	0.489	1.9E-02
201615_x_at	*CALD1*	caldesmon 1	0.489	1.9E-02
200903_s_at	*AHCY*	adenosylhomocysteinase	0.488	1.9E-02
219073_s_at	*OSBPL10*	oxysterol binding protein-like 10	0.488	1.9E-02
228559_at	*CENPN*	centromere protein N	0.488	1.9E-02
204998_s_at	*ATF5*	activating transcription factor 5	0.488	1.9E-02
223159_s_at	*NEK6*	NIMA (never in mitosis gene a)-related kinase 6	0.488	1.9E-02
201896_s_at	*PSRC1*	proline/serine-rich coiled-coil 1	0.487	1.9E-02
207828_s_at	*CENPF*	centromere protein F, 350/400kDa (mitosin)	0.487	1.9E-02
117_at	*HSPA6*	heat shock 70kDa protein 6 (HSP70B')	0.486	1.9E-02
204641_at	*NEK2*	NIMA (never in mitosis gene a)-related kinase 2	0.486	1.9E-02

Probeset ID	Gene symbol	Gene description	ρ	Adjusted *P*-value
201841_s_at	*HSPB1*	heat shock 27kDa protein 1	0.486	1.9E-02
212809_at	*NFATC2IP*	nuclear factor of activated T-cells, cytoplasmic, calcineurin-dependent 2 interacting protein	0.486	1.9E-02
222662_at	*PPP1R3B*	protein phosphatase 1, regulatory subunit 3B	0.486	1.9E-02
216253_s_at	*PARVB*	parvin, beta	0.486	2.0E-02
57163_at	*ELOVL1*	ELOVL fatty acid elongase 1	0.486	2.0E-02
216264_s_at	*LAMB2*	laminin, beta 2 (laminin S)	0.485	2.0E-02
200923_at	*LGALS3BP*	lectin, galactoside-binding, soluble, 3 binding protein	0.485	2.0E-02
225345_s_at	*FBXO32*	F-box protein 32	0.485	2.0E-02
224585_x_at	*ACTG1*	actin, gamma 1	0.485	2.0E-02
201550_x_at	*ACTG1*	actin, gamma 1	0.484	2.0E-02
202122_s_at	*PLIN3*	perilipin 3	0.484	2.0E-02
202868_s_at	*POP4*	processing of precursor 4, ribonuclease P/MRP subunit (S. cerevisiae)	0.483	2.0E-02
225898_at	*WDR54*	WD repeat domain 54	0.483	2.0E-02
235508_at	*PML*	promyelocytic leukemia	0.483	2.1E-02
219066_at	*PPCDC*	phosphopantothenoylcysteine decarboxylase	0.482	2.1E-02
217728_at	*S100A6*	S100 calcium binding protein A6	0.482	2.1E-02
225059_at	*AGTRAP*	angiotensin II receptor-associated protein	0.482	2.1E-02
213125_at	*OLFML2B*	olfactomedin-like 2B	0.482	2.1E-02
201555_at	*MCM3*	minichromosome maintenance complex component 3	0.481	2.1E-02
210999_s_at	*GRB10*	growth factor receptor-bound protein 10	0.481	2.1E-02
224864_at	*SRA1*	steroid receptor RNA activator 1	0.481	2.1E-02
1555758_a_at	*CDKN3*	cyclin-dependent kinase inhibitor 3	0.481	2.1E-02
209211_at	*KLF5*	Kruppel-like factor 5 (intestinal)	0.481	2.1E-02
220134_x_at	*FAM176B*	family with sequence similarity 176, member B	0.481	2.1E-02
212015_x_at	*PTBP1*	polypyrimidine tract binding protein 1	0.481	2.1E-02
238532_at	*DPF3*	D4, zinc and double PHD fingers, family 3	0.480	2.1E-02
2028_s_at	*E2F1*	E2F transcription factor 1	0.480	2.1E-02
204298_s_at	*LOX*	lysyl oxidase	0.480	2.1E-02
205479_s_at	*PLAU*	plasminogen activator, urokinase	0.480	2.1E-02
209100_at	*IFRD2*	interferon-related developmental regulator 2	0.480	2.1E-02
227313_at	*CNPY4*	canopy 4 homolog (zebrafish)	0.480	2.1E-02
204342_at	*SLC25A24*	solute carrier family 25 (mitochondrial carrier; phosphate carrier), member 24	0.480	2.1E-02
226217_at	*SLC30A7*	solute carrier family 30 (zinc transporter), member 7	0.480	2.1E-02
210023_s_at	*PCGF1*	polycomb group ring finger 1	0.479	2.2E-02
235834_at	*CALD1*	Caldesmon 1	0.479	2.2E-02
215566_x_at	*LYPLA2*	lysophospholipase II	0.479	2.2E-02
238075_at	*CHEK1*	checkpoint kinase 1	0.479	2.2E-02
237817_at	*SSR3*	signal sequence receptor, gamma (translocon-associated protein gamma)	0.479	2.2E-02
212364_at	*MYO1B*	myosin IB	0.479	2.2E-02
204252_at	*CDK2*	cyclin-dependent kinase 2	0.478	2.2E-02
218543_s_at	*PARP12*	poly (ADP-ribose) polymerase family, member 12	0.478	2.2E-02
225592_at	*NRM*	nurim (nuclear envelope membrane protein)	0.478	2.2E-02
37965_at	*PARVB*	parvin, beta	0.478	2.2E-02
201617_x_at	*CALD1*	caldesmon 1	0.478	2.2E-02
203967_at	*CDC6*	cell division cycle 6 homolog (S. cerevisiae)	0.478	2.2E-02

Probeset ID	Gene symbol	Gene description	ρ	Adjusted *P*-value
41220_at	*SEPT9*	septin 9	0.478	2.2E-02
217937_s_at	*HDAC7*	histone deacetylase 7	0.478	2.2E-02
212472_at	*MICAL2*	microtubule associated monoxygenase, calponin and LIM domain containing 2	0.477	2.2E-02
206662_at	*GLRX*	glutaredoxin (thioltransferase)	0.477	2.2E-02
215380_s_at	*GGCT*	gamma-glutamylcyclotransferase	0.477	2.2E-02
209408_at	*KIF2C*	kinesin family member 2C	0.477	2.2E-02
226294_x_at	*FAM91A1*	family with sequence similarity 91, member A1	0.477	2.2E-02
233333_x_at	*AVIL*	advillin	0.477	2.2E-02
221923_s_at	*NPM1*	nucleophosmin (nucleolar phosphoprotein B23, numatrin)	0.476	2.2E-02
229587_at	*UBA2*	ubiquitin-like modifier activating enzyme 2	0.476	2.2E-02
207011_s_at	*PTK7*	PTK7 protein tyrosine kinase 7	0.476	2.2E-02
203554_x_at	*PTTG1*	pituitary tumor-transforming 1	0.476	2.2E-02
208436_s_at	*IRF7*	interferon regulatory factor 7	0.476	2.2E-02
200827_at	*PLOD1*	procollagen-lysine, 2-oxoglutarate 5-dioxygenase 1	0.476	2.2E-02
211970_x_at	*ACTG1*	actin, gamma 1	0.476	2.2E-02
209709_s_at	*HMMR*	hyaluronan-mediated motility receptor (RHAMM)	0.476	2.3E-02
212312_at	*BCL2L1*	BCL2-like 1	0.475	2.3E-02
204822_at	*TTK*	TTK protein kinase	0.475	2.3E-02
212320_at	*TUBB*	tubulin, beta class I	0.475	2.3E-02
220968_s_at	*TSPAN9*	tetraspanin 9	0.475	2.3E-02
201616_s_at	*CALD1*	caldesmon 1	0.474	2.3E-02
203325_s_at	*COL5A1*	collagen, type V, alpha 1	0.474	2.3E-02
213911_s_at	*H2AFZ*	H2A histone family, member Z	0.474	2.3E-02
214710_s_at	*CCNB1*	cyclin B1	0.474	2.3E-02
202756_s_at	*GPC1*	glypican 1	0.474	2.3E-02
218983_at	*C1RL*	complement component 1, r subcomponent-like	0.474	2.3E-02
208659_at	*CLIC1*	chloride intracellular channel 1	0.473	2.3E-02
207808_s_at	*PROS1*	protein S (alpha)	0.473	2.4E-02
218541_s_at	*C8orf4*	chromosome 8 open reading frame 4	0.473	2.4E-02
203240_at	*FCGBP*	Fc fragment of IgG binding protein	0.473	2.4E-02
205547_s_at	*TAGLN*	transgelin	0.472	2.4E-02
223158_s_at	*NEK6*	NIMA (never in mitosis gene a)-related kinase 6	0.472	2.4E-02
223389_s_at	*ZNF581*	zinc finger protein 581	0.472	2.4E-02
209806_at	*HIST1H2BK*	histone cluster 1, H2bk	0.472	2.4E-02
202709_at	*FMOD*	fibromodulin	0.472	2.4E-02
32811_at	*MYO1C*	myosin IC	0.472	2.4E-02
201508_at	*IGFBP4*	insulin-like growth factor binding protein 4	0.472	2.4E-02
201097_s_at	*ARF4*	ADP-ribosylation factor 4	0.471	2.4E-02
219178_at	*QTRTD1*	queuine tRNA-ribosyltransferase domain containing 1	0.471	2.4E-02
202655_at	*MANF*	mesencephalic astrocyte-derived neurotrophic factor	0.471	2.4E-02
211945_s_at	*ITGB1*	integrin, beta 1 (fibronectin receptor, beta polypeptide, antigen CD29 includes MDF2, MSK12)	0.471	2.4E-02
218840_s_at	*NADSYN1*	NAD synthetase 1	0.471	2.4E-02
160020_at	*MMP14*	matrix metallopeptidase 14 (membrane-inserted)	0.471	2.4E-02
210355_at	*PTHLH*	parathyroid hormone-like hormone	0.471	2.4E-02
216237_s_at	*MCM5*	minichromosome maintenance complex component 5	0.471	2.4E-02
226071_at	*ADAMTSL4*	ADAMTS-like 4	0.471	2.4E-02

Probeset ID	Gene symbol	Gene description	ρ	Adjusted *P*-value
202856_s_at	*SLC16A3*	solute carrier family 16, member 3 (monocarboxylic acid transporter 4)	0.470	2.5E-02
211983_x_at	*ACTG1*	actin, gamma 1	0.469	2.5E-02
230448_at	*SLC38A10*	solute carrier family 38, member 10	0.469	2.5E-02
1553764_a_at	*AJUBA*	ajuba LIM protein	0.469	2.5E-02
212431_at	*HMGXB3*	HMG box domain containing 3	0.469	2.5E-02
225128_at	*KDELC2*	KDEL (Lys-Asp-Glu-Leu) containing 2	0.469	2.5E-02
218443_s_at	*DAZAP1*	DAZ associated protein 1	0.469	2.5E-02
200656_s_at	*P4HB*	prolyl 4-hydroxylase, beta polypeptide	0.469	2.5E-02
222848_at	*CENPK*	centromere protein K	0.469	2.5E-02
230518_at	*MPZL2*	myelin protein zero-like 2	0.468	2.5E-02
221607_x_at	*ACTG1*	actin, gamma 1	0.468	2.5E-02
228280_at	*ZC3HAV1L*	zinc finger CCCH-type, antiviral 1-like	0.468	2.5E-02
227188_at	*FAM176C*	family with sequence similarity 176, member C	0.468	2.6E-02
227295_at	*IKBIP*	IKBKB interacting protein	0.468	2.6E-02
217784_at	*YKT6*	YKT6 v-SNARE homolog (S. cerevisiae)	0.467	2.6E-02
204825_at	*MELK*	maternal embryonic leucine zipper kinase	0.467	2.6E-02
227894_at	*WDR90*	WD repeat domain 90	0.467	2.6E-02
201818_at	*LPCAT1*	lysophosphatidylcholine acyltransferase 1	0.467	2.6E-02
213075_at	*OLFML2A*	olfactomedin-like 2A	0.467	2.6E-02
213923_at	*RAP2B*	RAP2B, member of RAS oncogene family	0.467	2.6E-02
204709_s_at	*KIF23*	kinesin family member 23	0.466	2.6E-02
218248_at	*FAM111A*	family with sequence similarity 111, member A	0.466	2.6E-02
218618_s_at	*FNDC3B*	fibronectin type III domain containing 3B	0.466	2.6E-02
223700_at	*MND1*	meiotic nuclear divisions 1 homolog (S. cerevisiae)	0.466	2.6E-02
227038_at	*SGMS2*	sphingomyelin synthase 2	0.466	2.6E-02
209356_x_at	*EFEMP2*	EGF containing fibulin-like extracellular matrix protein 2	0.465	2.6E-02
219918_s_at	*ASPM*	asp (abnormal spindle) homolog, microcephaly associated (Drosophila)	0.465	2.7E-02
219957_at	*RUFY2*	RUN and FYVE domain containing 2	0.465	2.7E-02
221537_at	*PLXNA1*	plexin A1	0.465	2.7E-02
222654_at	*IMPAD1*	inositol monophosphatase domain containing 1	0.465	2.7E-02
220954_s_at	*PILRB*	paired immunoglobin-like type 2 receptor beta	0.464	2.7E-02
235113_at	*LRR1*	leucine rich repeat protein 1	0.464	2.7E-02
225314_at	*OCIAD2*	OCIA domain containing 2	0.464	2.7E-02
200699_at	*KDELR2*	KDEL (Lys-Asp-Glu-Leu) endoplasmic reticulum protein retention receptor 2	0.463	2.7E-02
202705_at	*CCNB2*	cyclin B2	0.463	2.7E-02
230165_at	*SGOL2*	shugoshin-like 2 (S. pombe)	0.463	2.7E-02
238756_at	*GAS2L3*	growth arrest-specific 2 like 3	0.463	2.7E-02
200844_s_at	*PRDX6*	peroxiredoxin 6	0.463	2.7E-02
1553978_at	*MEF2BNB*	MEF2B neighbor	0.463	2.8E-02
213790_at	*ADAM12*	ADAM metallopeptidase domain 12	0.463	2.8E-02
213446_s_at	*IQGAP1*	IQ motif containing GTPase activating protein 1	0.462	2.8E-02
1555997_s_at	*IGFBP5*	insulin-like growth factor binding protein 5	0.462	2.8E-02
202589_at	*TYMS*	thymidylate synthetase	0.461	2.8E-02
202804_at	*ABCC1*	ATP-binding cassette, sub-family C (CFTR/MRP), member 1	0.461	2.8E-02
224598_at	*MGAT4B*	mannosyl (alpha-1,3-)-glycoprotein beta-1,4-N-	0.461	2.8E-02

Probeset ID	Gene symbol	Gene description	ρ	Adjusted *P*-value
		acetylglucosaminyltransferase, isozyme B		
225269_s_at	*RBMS1*	RNA binding motif, single stranded interacting protein 1	0.461	2.8E-02
222512_at	*NUB1*	negative regulator of ubiquitin-like proteins 1	0.460	2.9E-02
226936_at	*CENPW*	centromere protein W	0.460	2.9E-02
209884_s_at	*SLC4A7*	solute carrier family 4, sodium bicarbonate cotransporter, member 7	0.460	2.9E-02
219890_at	*CLEC5A*	C-type lectin domain family 5, member A	0.460	2.9E-02
226814_at	*ADAMTS9*	ADAM metallopeptidase with thrombospondin type 1 motif, 9	0.460	2.9E-02
203044_at	*CHSY1*	chondroitin sulfate synthase 1	0.460	2.9E-02
34260_at	*TELO2*	TEL2, telomere maintenance 2, homolog (S. cerevisiae)	0.460	2.9E-02
208690_s_at	*PDLIM1*	PDZ and LIM domain 1	0.460	2.9E-02
204078_at	*LEPREL4*	leprecan-like 4	0.460	2.9E-02
223480_s_at	*MRPL47*	mitochondrial ribosomal protein L47	0.459	2.9E-02
213521_at	*PTPN18*	protein tyrosine phosphatase, non-receptor type 18 (brain-derived)	0.459	2.9E-02
1570153_at	*CCDC169*	coiled-coil domain containing 169	0.458	2.9E-02
232099_at	*PCDHB16*	protocadherin beta 16	0.458	2.9E-02
213556_at	*LOC390940*	uncharacterized protein ENSP00000244321	0.458	3.0E-02
236641_at	*KIF14*	kinesin family member 14	0.458	3.0E-02
226912_at	*ZDHHC23*	zinc finger, DHHC-type containing 23	0.457	3.0E-02
204490_s_at	*CD44*	CD44 molecule (Indian blood group)	0.457	3.0E-02
202878_s_at	*CD93*	CD93 molecule	0.457	3.0E-02
209171_at	*ITPA*	inosine triphosphatase (nucleoside triphosphate pyrophosphatase)	0.457	3.0E-02
201578_at	*PODXL*	podocalyxin-like	0.457	3.0E-02
225868_at	*TRIM47*	tripartite motif containing 47	0.457	3.0E-02
202446_s_at	*PLSCR1*	phospholipid scramblase 1	0.457	3.0E-02
202620_s_at	*PLOD2*	procollagen-lysine, 2-oxoglutarate 5-dioxygenase 2	0.456	3.0E-02
204163_at	*EMILIN1*	elastin microfibril interfacer 1	0.456	3.0E-02
202234_s_at	*SLC16A1*	solute carrier family 16, member 1 (monocarboxylic acid transporter 1)	0.456	3.0E-02
205690_s_at	*BUD31*	BUD31 homolog (S. cerevisiae)	0.456	3.0E-02
225263_at	*HS6ST1*	heparan sulfate 6-O-sulfotransferase 1	0.456	3.1E-02
221739_at	*C19orf10*	chromosome 19 open reading frame 10	0.456	3.1E-02
212143_s_at	*IGFBP3*	insulin-like growth factor binding protein 3	0.455	3.1E-02
222606_at	*ZWILCH*	Zwilch, kinetochore associated, homolog (Drosophila)	0.455	3.1E-02
202894_at	*EPHB4*	EPH receptor B4	0.455	3.1E-02
222978_at	*SURF4*	surfeit 4	0.455	3.1E-02
226353_at	*SPPL2A*	signal peptide peptidase like 2A	0.455	3.1E-02
218368_s_at	*TNFRSF12A*	tumor necrosis factor receptor superfamily, member 12A	0.454	3.1E-02
222217_s_at	*SLC27A3*	solute carrier family 27 (fatty acid transporter), member 3	0.454	3.1E-02
219973_at	*ARSJ*	arylsulfatase family, member J	0.454	3.1E-02
1553984_s_at	*DTYMK*	deoxythymidylate kinase (thymidylate kinase)	0.454	3.1E-02
209150_s_at	*TM9SF1*	transmembrane 9 superfamily member 1	0.454	3.1E-02
203625_x_at	*SKP2*	S-phase kinase-associated protein 2, E3 ubiquitin protein ligase	0.454	3.1E-02
212255_s_at	*ATP2C1*	ATPase, Ca++ transporting, type 2C, member 1	0.453	3.2E-02
230258_at	*GLIS3*	GLIS family zinc finger 3	0.453	3.2E-02
223417_at	*RAD18*	RAD18 homolog (S. cerevisiae)	0.453	3.2E-02

Probeset ID	Gene symbol	Gene description	ρ	Adjusted *P*-value
35626_at	*SGSH*	N-sulfoglucosamine sulfohydrolase	0.453	3.2E-02
1560116_a_at	*NEDD1*	neural precursor cell expressed, developmentally down-regulated 1	0.453	3.2E-02
33323_r_at	*SFN*	stratifin	0.453	3.2E-02
201063_at	*RCN1*	reticulocalbin 1, EF-hand calcium binding domain	0.452	3.2E-02
200698_at	*KDELR2*	KDEL (Lys-Asp-Glu-Leu) endoplasmic reticulum protein retention receptor 2	0.452	3.2E-02
226980_at	*DEPDC1B*	DEP domain containing 1B	0.452	3.2E-02
229551_x_at	*ZNF367*	zinc finger protein 367	0.452	3.2E-02
221245_s_at	*FZD5*	frizzled family receptor 5	0.452	3.2E-02
232381_s_at	*DNAH5*	dynein, axonemal, heavy chain 5	0.452	3.2E-02
235158_at	*TMEM209*	transmembrane protein 209	0.452	3.2E-02
213857_s_at	*CD47*	CD47 molecule	0.452	3.2E-02
223495_at	*CCDC8*	coiled-coil domain containing 8	0.452	3.2E-02
212920_at	*REST*	RE1-silencing transcription factor	0.452	3.2E-02
207443_at	*NR2E1*	nuclear receptor subfamily 2, group E, member 1	0.451	3.3E-02
204883_s_at	*HUS1*	HUS1 checkpoint homolog (S. pombe)	0.451	3.3E-02
200700_s_at	*KDELR2*	KDEL (Lys-Asp-Glu-Leu) endoplasmic reticulum protein retention receptor 2	0.450	3.3E-02
218388_at	*PGLS*	6-phosphogluconolactonase	0.450	3.3E-02
213943_at	*TWIST1*	twist homolog 1 (Drosophila)	0.450	3.3E-02
223220_s_at	*PARP9*	poly (ADP-ribose) polymerase family, member 9	0.450	3.3E-02
209835_x_at	*CD44*	CD44 molecule (Indian blood group)	0.450	3.3E-02
225945_at	*ZNF655*	zinc finger protein 655	0.450	3.3E-02
201477_s_at	*RRM1*	ribonucleotide reductase M1	0.450	3.4E-02
204929_s_at	*VAMP5*	vesicle-associated membrane protein 5 (myobrevin)	0.449	3.4E-02
227361_at	*HS3ST3B1*	heparan sulfate (glucosamine) 3-O-sulfotransferase 3B1	0.449	3.4E-02
206102_at	*GINS1*	GINS complex subunit 1 (Psf1 homolog)	0.449	3.4E-02
219539_at	*GEMIN6*	gem (nuclear organelle) associated protein 6	0.449	3.4E-02
209264_s_at	*TSPAN4*	tetraspanin 4	0.449	3.4E-02
209302_at	*POLR2H*	polymerase (RNA) II (DNA directed) polypeptide H	0.449	3.4E-02
211015_s_at	*HSPA4*	heat shock 70kDa protein 4	0.449	3.4E-02
206111_at	*RNASE2*	ribonuclease, RNase A family, 2 (liver, eosinophil-derived neurotoxin)	0.449	3.4E-02
201469_s_at	*SHC1*	SHC (Src homology 2 domain containing) transforming protein 1	0.449	3.4E-02
201655_s_at	*HSPG2*	heparan sulfate proteoglycan 2	0.449	3.4E-02
206855_s_at	*HYAL2*	hyaluronoglucosaminidase 2	0.449	3.4E-02
209410_s_at	*GRB10*	growth factor receptor-bound protein 10	0.449	3.4E-02
225075_at	*PDRG1*	p53 and DNA-damage regulated 1	0.448	3.4E-02
201292_at	*TOP2A*	topoisomerase (DNA) II alpha 170kDa	0.448	3.4E-02
219331_s_at	*KLHDC8A*	kelch domain containing 8A	0.448	3.4E-02
221845_s_at	*CLPB*	ClpB caseinolytic peptidase B homolog (E. coli)	0.448	3.4E-02
229305_at	*MLF1IP*	MLF1 interacting protein	0.448	3.4E-02
212921_at	*SMYD2*	SET and MYND domain containing 2	0.447	3.5E-02
209708_at	*MOXD1*	monooxygenase, DBH-like 1	0.447	3.5E-02
219787_s_at	*ECT2*	epithelial cell transforming sequence 2 oncogene	0.447	3.5E-02
204159_at	*CDKN2C*	cyclin-dependent kinase inhibitor 2C (p18, inhibits CDK4)	0.447	3.5E-02
209267_s_at	*SLC39A8*	solute carrier family 39 (zinc transporter), member 8	0.447	3.5E-02

Probeset ID	Gene symbol	Gene description	ρ	Adjusted *P*-value
201291_s_at	*TOP2A*	topoisomerase (DNA) II alpha 170kDa	0.446	3.5E-02
223835_x_at	*OTP*	orthopedia homeobox	0.446	3.5E-02
219203_at	*EMC9*	ER membrane protein complex subunit 9	0.446	3.5E-02
211926_s_at	*MYH9*	myosin, heavy chain 9, non-muscle	0.446	3.5E-02
200601_at	*ACTN4*	actinin, alpha 4	0.446	3.5E-02
232238_at	*ASPM*	asp (abnormal spindle) homolog, microcephaly associated (Drosophila)	0.446	3.5E-02
201850_at	*CAPG*	capping protein (actin filament), gelsolin-like	0.446	3.5E-02
202766_s_at	*FBN1*	fibrillin 1	0.446	3.5E-02
207455_at	*P2RY1*	purinergic receptor P2Y, G-protein coupled, 1	0.446	3.5E-02
212646_at	*RFTN1*	raftlin, lipid raft linker 1	0.446	3.5E-02
231406_at	*ORAI2*	ORAI calcium release-activated calcium modulator 2	0.445	3.6E-02
203649_s_at	*PLA2G2A*	phospholipase A2, group IIA (platelets, synovial fluid)	0.445	3.6E-02
223172_s_at	*MTFP1*	mitochondrial fission process 1	0.445	3.6E-02
202801_at	*PRKACA*	protein kinase, cAMP-dependent, catalytic, alpha	0.445	3.6E-02
211042_x_at	*MCAM*	melanoma cell adhesion molecule	0.445	3.6E-02
218807_at	*VAV3*	vav 3 guanine nucleotide exchange factor	0.445	3.6E-02
235384_at	*NUDT19*	nudix (nucleoside diphosphate linked moiety X)-type motif 19	0.445	3.6E-02
213616_at	*TPGS2*	tubulin polyglutamylase complex subunit 2	0.444	3.6E-02
203947_at	*CSTF3*	cleavage stimulation factor, 3' pre-RNA, subunit 3, 77kDa	0.444	3.6E-02
204175_at	*ZNF593*	zinc finger protein 593	0.444	3.6E-02
213076_at	*ITPKC*	inositol-trisphosphate 3-kinase C	0.444	3.6E-02
207643_s_at	*TNFRSF1A*	tumor necrosis factor receptor superfamily, member 1A	0.444	3.6E-02
208368_s_at	*BRCA2*	breast cancer 2, early onset	0.444	3.6E-02
223474_at	*IRF2BPL*	interferon regulatory factor 2 binding protein-like	0.444	3.6E-02
209969_s_at	*STAT1*	signal transducer and activator of transcription 1, 91kDa	0.444	3.6E-02
226949_at	*GOLGA3*	golgin A3	0.444	3.6E-02
202805_s_at	*ABCC1*	ATP-binding cassette, sub-family C (CFTR/MRP), member 1	0.443	3.6E-02
234294_x_at	*GATAD2A*	GATA zinc finger domain containing 2A	0.443	3.7E-02
213008_at	*FANCI*	Fanconi anemia, complementation group I	0.443	3.7E-02
218081_at	*C20orf27*	chromosome 20 open reading frame 27	0.443	3.7E-02
204068_at	*STK3*	serine/threonine kinase 3	0.443	3.7E-02
216606_x_at	*LYPLA2*	lysophospholipase II	0.443	3.7E-02
235609_at	*BRIP1*	BRCA1 interacting protein C-terminal helicase 1	0.443	3.7E-02
203234_at	*UPP1*	uridine phosphorylase 1	0.442	3.7E-02
224749_at	*ITFG3*	integrin alpha FG-GAP repeat containing 3	0.442	3.7E-02
234974_at	*GALM*	galactose mutarotase (aldose 1-epimerase)	0.442	3.7E-02
34868_at	*SMG5*	smg-5 homolog, nonsense mediated mRNA decay factor (C. elegans)	0.442	3.7E-02
218039_at	*NUSAP1*	nucleolar and spindle associated protein 1	0.442	3.7E-02
33322_i_at	*SFN*	stratifin	0.442	3.7E-02
223247_at	*MED10*	mediator complex subunit 10	0.442	3.7E-02
202737_s_at	*LSM4*	LSM4 homolog, U6 small nuclear RNA associated (S. cerevisiae)	0.442	3.7E-02
212992_at	*AHNAK2*	AHNAK nucleoprotein 2	0.442	3.7E-02
212363_x_at	*ACTG1*	actin, gamma 1	0.442	3.7E-02
217104_at	*ST20*	suppressor of tumorigenicity 20	0.442	3.7E-02

Probeset ID	Gene symbol	Gene description	ρ	Adjusted *P*-value
218663_at	*NCAPG*	non-SMC condensin I complex, subunit G	0.441	3.8E-02
203425_s_at	*IGFBP5*	insulin-like growth factor binding protein 5	0.441	3.8E-02
204865_at	*CA3*	carbonic anhydrase III, muscle specific	0.441	3.8E-02
206011_at	*CASP1*	caspase 1, apoptosis-related cysteine peptidase	0.441	3.8E-02
204357_s_at	*LIMK1*	LIM domain kinase 1	0.440	3.8E-02
219004_s_at	*MIS18A*	MIS18 kinetochore protein homolog A (S. pombe)	0.440	3.8E-02
229435_at	*GLIS3*	GLIS family zinc finger 3	0.440	3.8E-02
215947_s_at	*FAM136A*	family with sequence similarity 136, member A	0.439	3.9E-02
222231_s_at	*LRRC59*	leucine rich repeat containing 59	0.439	3.9E-02
209561_at	*THBS3*	thrombospondin 3	0.439	3.9E-02
222958_s_at	*DEPDC1*	DEP domain containing 1	0.439	3.9E-02
227792_at	*ITPRIPL2*	inositol 1,4,5-trisphosphate receptor interacting protein-like 2	0.439	3.9E-02
226935_s_at	*CLPTM1L*	CLPTM1-like	0.439	3.9E-02
204471_at	*GAP43*	growth associated protein 43	0.438	3.9E-02
219885_at	*SLFN12*	schlafen family member 12	0.438	3.9E-02
218883_s_at	*MLF1IP*	MLF1 interacting protein	0.438	3.9E-02
206316_s_at	*KNTC1*	kinetochore associated 1	0.438	4.0E-02
202998_s_at	*LOXL2*	lysyl oxidase-like 2	0.438	4.0E-02
223394_at	*SERTAD1*	SERTA domain containing 1	0.438	4.0E-02
215446_s_at	*LOX*	lysyl oxidase	0.438	4.0E-02
200961_at	*SEPHS2*	selenophosphate synthetase 2	0.438	4.0E-02
1555724_s_at	*TAGLN*	transgelin	0.437	4.0E-02
201251_at	*PKM*	pyruvate kinase, muscle	0.437	4.0E-02
203270_at	*DTYMK*	deoxythymidylate kinase (thymidylate kinase)	0.437	4.0E-02
209082_s_at	*COL18A1*	collagen, type XVIII, alpha 1	0.437	4.0E-02
202408_s_at	*PRPF31*	PRP31 pre-mRNA processing factor 31 homolog (S. cerevisiae)	0.437	4.0E-02
208370_s_at	*RCAN1*	regulator of calcineurin 1	0.437	4.0E-02
227480_at	*SUSD2*	sushi domain containing 2	0.437	4.0E-02
218095_s_at	*TMEM165*	transmembrane protein 165	0.437	4.0E-02
212298_at	*NRP1*	neuropilin 1	0.436	4.0E-02
203167_at	*TIMP2*	TIMP metallopeptidase inhibitor 2	0.436	4.0E-02
213861_s_at	*METTL21B*	methyltransferase like 21B	0.436	4.0E-02
218159_at	*DDRGK1*	DDRGK domain containing 1	0.436	4.0E-02
219293_s_at	*OLA1*	Obg-like ATPase 1	0.436	4.0E-02
201663_s_at	*SMC4*	structural maintenance of chromosomes 4	0.435	4.1E-02
209900_s_at	*SLC16A1*	solute carrier family 16, member 1 (monocarboxylic acid transporter 1)	0.435	4.1E-02
217933_s_at	*LAP3*	leucine aminopeptidase 3	0.435	4.1E-02
205211_s_at	*RIN1*	Ras and Rab interactor 1	0.435	4.1E-02
201088_at	*KPNA2*	karyopherin alpha 2 (RAG cohort 1, importin alpha 1)	0.435	4.1E-02
218009_s_at	*PRC1*	protein regulator of cytokinesis 1	0.434	4.1E-02
202951_at	*STK38*	serine/threonine kinase 38	0.434	4.1E-02
205394_at	*CHEK1*	checkpoint kinase 1	0.434	4.1E-02
228188_at	*FOSL2*	FOS-like antigen 2	0.434	4.1E-02
211977_at	*GPR107*	G protein-coupled receptor 107	0.434	4.1E-02
223165_s_at	*IP6K2*	inositol hexakisphosphate kinase 2	0.434	4.1E-02
228281_at	*C11orf82*	chromosome 11 open reading frame 82	0.434	4.1E-02

Probeset ID	Gene symbol	Gene description	ρ	Adjusted *P*-value
231249_at	*SZT2*	seizure threshold 2 homolog (mouse)	0.434	4.2E-02
201798_s_at	*MYOF*	myoferlin	0.434	4.2E-02
36936_at	*TSTA3*	tissue specific transplantation antigen P35B	0.434	4.2E-02
227467_at	*RDH10*	retinol dehydrogenase 10 (all-trans)	0.434	4.2E-02
228729_at	*CCNB1*	cyclin B1	0.434	4.2E-02
202912_at	*ADM*	adrenomedullin	0.433	4.2E-02
227249_at	*NDE1*	NudE nuclear distribution E homolog 1 (A. nidulans)	0.433	4.2E-02
212063_at	*CD44*	CD44 molecule (Indian blood group)	0.433	4.2E-02
217855_x_at	*SDF4*	stromal cell derived factor 4	0.433	4.2E-02
219588_s_at	*NCAPG2*	non-SMC condensin II complex, subunit G2	0.433	4.2E-02
225969_at	*ALKBH6*	alkB, alkylation repair homolog 6 (E. coli)	0.433	4.2E-02
221274_s_at	*LMAN2L*	lectin, mannose-binding 2-like	0.433	4.2E-02
212219_at	*PSME4*	proteasome (prosome, macropain) activator subunit 4	0.433	4.2E-02
212280_x_at	*ATG4B*	autophagy related 4B, cysteine peptidase	0.433	4.2E-02
227352_at	*SWSAP1*	SWIM-type zinc finger 7 associated protein 1	0.432	4.2E-02
200650_s_at	*LDHA*	lactate dehydrogenase A	0.432	4.3E-02
1555167_s_at	*NAMPT*	nicotinamide phosphoribosyltransferase	0.432	4.3E-02
208808_s_at	*HMGB2*	high mobility group box 2	0.432	4.3E-02
227100_at	*B3GALTL*	beta 1,3-galactosyltransferase-like	0.432	4.3E-02
233734_s_at	*OSBPL5*	oxysterol binding protein-like 5	0.432	4.3E-02
212533_at	*WEE1*	WEE1 homolog (S. pombe)	0.431	4.3E-02
216262_s_at	*TGIF2*	TGFB-induced factor homeobox 2	0.431	4.3E-02
228873_at	*COL22A1*	collagen, type XXII, alpha 1	0.431	4.3E-02
221521_s_at	*GINS2*	GINS complex subunit 2 (Psf2 homolog)	0.431	4.3E-02
31837_at	*LMF2*	lipase maturation factor 2	0.431	4.3E-02
201560_at	*CLIC4*	chloride intracellular channel 4	0.431	4.3E-02
221794_at	*DOCK6*	dedicator of cytokinesis 6	0.431	4.3E-02
242041_at	*CSPP1*	centrosome and spindle pole associated protein 1	0.431	4.3E-02
221605_s_at	*PIPOX*	pipecolic acid oxidase	0.431	4.3E-02
222067_x_at	*HIST1H2BD*	histone cluster 1, H2bd	0.431	4.3E-02
203133_at	*SEC61B*	Sec61 beta subunit	0.431	4.3E-02
225019_at	*CAMK2D*	calcium/calmodulin-dependent protein kinase II delta	0.431	4.3E-02
203731_s_at	*ZKSCAN5*	zinc finger with KRAB and SCAN domains 5	0.430	4.3E-02
205918_at	*SLC4A3*	solute carrier family 4, anion exchanger, member 3	0.430	4.4E-02
204779_s_at	*HOXB7*	homeobox B7	0.430	4.4E-02
213379_at	*COQ2*	coenzyme Q2 homolog, prenyltransferase (yeast)	0.430	4.4E-02
201252_at	*PSMC4*	proteasome (prosome, macropain) 26S subunit, ATPase, 4	0.430	4.4E-02
1560916_a_at	*DPY19L1*	dpy-19-like 1 (C. elegans)	0.429	4.4E-02
202270_at	*GBP1*	guanylate binding protein 1, interferon-inducible	0.429	4.4E-02
212014_x_at	*CD44*	CD44 molecule (Indian blood group)	0.429	4.4E-02
202855_s_at	*SLC16A3*	solute carrier family 16, member 3 (monocarboxylic acid transporter 4)	0.429	4.4E-02
209642_at	*BUB1*	budding uninhibited by benzimidazoles 1 homolog (yeast)	0.429	4.4E-02
207467_x_at	*CAST*	calpastatin	0.429	4.4E-02
230836_at	*ST8SIA4*	ST8 alpha-N-acetyl-neuraminide alpha-2,8-sialyltransferase 4	0.429	4.4E-02
203578_s_at	*SLC7A6*	solute carrier family 7 (amino acid transporter light chain,	0.429	4.5E-02

Probeset ID	Gene symbol	Gene description	ρ	Adjusted *P*-value
		y+L system), member 6		
217854_s_at	*POLR2E*	polymerase (RNA) II (DNA directed) polypeptide E, 25kDa	0.429	4.5E-02
201673_s_at	*GYS1*	glycogen synthase 1 (muscle)	0.428	4.5E-02
225801_at	*FBXO32*	F-box protein 32	0.428	4.5E-02
202729_s_at	*LTBP1*	latent transforming growth factor beta binding protein 1	0.428	4.5E-02
243403_x_at	*CPM*	carboxypeptidase M	0.428	4.5E-02
200829_x_at	*ZNF207*	zinc finger protein 207	0.428	4.5E-02
219257_s_at	*SPHK1*	sphingosine kinase 1	0.428	4.5E-02
231823_s_at	*SH3PXD2B*	SH3 and PX domains 2B	0.428	4.5E-02
201726_at	*ELAVL1*	ELAV (embryonic lethal, abnormal vision, Drosophila)-like 1 (Hu antigen R)	0.428	4.5E-02
204162_at	*NDC80*	NDC80 kinetochore complex component homolog (S. cerevisiae)	0.428	4.5E-02
219933_at	*GLRX2*	glutaredoxin 2	0.428	4.5E-02
223553_s_at	*DOK3*	docking protein 3	0.427	4.5E-02
218018_at	*PDXK*	pyridoxal (pyridoxine, vitamin B6) kinase	0.427	4.5E-02
207610_s_at	*EMR2*	egf-like module containing, mucin-like, hormone receptor-like 2	0.427	4.5E-02
211864_s_at	*MYOF*	myoferlin	0.427	4.6E-02
219183_s_at	*CYTH4*	cytohesin 4	0.427	4.6E-02
203022_at	*RNASEH2A*	ribonuclease H2, subunit A	0.426	4.6E-02
223381_at	*NUF2*	NUF2, NDC80 kinetochore complex component, homolog (S. cerevisiae)	0.426	4.6E-02
201645_at	*TNC*	tenascin C	0.426	4.6E-02
214452_at	*BCAT1*	branched chain amino-acid transaminase 1, cytosolic	0.426	4.6E-02
221827_at	*RBCK1*	RanBP-type and C3HC4-type zinc finger containing 1	0.426	4.6E-02
204262_s_at	*PSEN2*	presenilin 2 (Alzheimer disease 4)	0.425	4.7E-02
208091_s_at	*VOPP1*	vesicular, overexpressed in cancer, prosurvival protein 1	0.425	4.7E-02
64440_at	*IL17RC*	interleukin 17 receptor C	0.425	4.7E-02
200611_s_at	*WDR1*	WD repeat domain 1	0.425	4.7E-02
202133_at	*WWTR1*	WW domain containing transcription regulator 1	0.425	4.7E-02
211963_s_at	*ARPC5*	actin related protein 2/3 complex, subunit 5, 16kDa	0.425	4.7E-02
201765_s_at	*HEXA*	hexosaminidase A (alpha polypeptide)	0.425	4.7E-02
208691_at	*TFRC*	transferrin receptor (p90, CD71)	0.425	4.7E-02
200678_x_at	*GRN*	granulin	0.424	4.7E-02
221258_s_at	*KIF18A*	kinesin family member 18A	0.424	4.7E-02
227515_at	*STAMBP*	STAM binding protein	0.424	4.7E-02
201937_s_at	*DNPEP*	aspartyl aminopeptidase	0.424	4.7E-02
226063_at	*VAV2*	vav 2 guanine nucleotide exchange factor	0.424	4.7E-02
212621_at	*TMEM194A*	transmembrane protein 194A	0.423	4.8E-02
214426_x_at	*CHAF1A*	chromatin assembly factor 1, subunit A (p150)	0.423	4.8E-02
243296_at	*NAMPT*	Nicotinamide phosphoribosyltransferase	0.423	4.8E-02
223396_at	*TMEM60*	transmembrane protein 60	0.423	4.8E-02
217751_at	*GSTK1*	glutathione S-transferase kappa 1	0.423	4.8E-02
220189_s_at	*MGAT4B*	mannosyl (alpha-1,3-)-glycoprotein beta-1,4-N-acetylglucosaminyltransferase, isozyme B	0.423	4.8E-02
210423_s_at	*SLC11A1*	solute carrier family 11 (proton-coupled divalent metal ion transporters), member 1	0.423	4.8E-02
221653_x_at	*APOL2*	apolipoprotein L, 2	0.423	4.8E-02

Probeset ID	Gene symbol	Gene description	ρ	Adjusted *P*-value
226385_s_at	*MALSU1*	mitochondrial assembly of ribosomal large subunit 1	0.423	4.8E-02
222684_s_at	*NOL10*	nucleolar protein 10	0.423	4.8E-02
234005_x_at	*STK36*	serine/threonine kinase 36	0.423	4.8E-02
1554314_at	*C6orf141*	chromosome 6 open reading frame 141	0.423	4.8E-02
204402_at	*RHBDD3*	rhomboid domain containing 3	0.423	4.8E-02
200891_s_at	*SSR1*	signal sequence receptor, alpha	0.423	4.8E-02
218131_s_at	*GATAD2A*	GATA zinc finger domain containing 2A	0.422	4.8E-02
222690_s_at	*TMEM39A*	transmembrane protein 39A	0.422	4.8E-02
225614_at	*SAAL1*	serum amyloid A-like 1	0.422	4.8E-02
228597_at	*MIS18A*	MIS18 kinetochore protein homolog A (S. pombe)	0.422	4.8E-02
205298_s_at	*BTN2A2*	butyrophilin, subfamily 2, member A2	0.422	4.8E-02
210052_s_at	*TPX2*	TPX2, microtubule-associated, homolog (Xenopus laevis)	0.422	4.8E-02
225524_at	*ANTXR2*	anthrax toxin receptor 2	0.422	4.9E-02
205542_at	*STEAP1*	six transmembrane epithelial antigen of the prostate 1	0.422	4.9E-02
226452_at	*PDK1*	pyruvate dehydrogenase kinase, isozyme 1	0.421	4.9E-02
200931_s_at	*VCL*	vinculin	0.421	4.9E-02
217788_s_at	*GALNT2*	UDP-N-acetyl-alpha-D-galactosamine:polypeptide N-acetylgalactosaminyltransferase 2 (GalNAc-T2)	0.421	4.9E-02
202628_s_at	*SERPINE1*	serpin peptidase inhibitor, clade E (nexin, plasminogen activator inhibitor type 1), member 1	0.421	4.9E-02
211747_s_at	*LSM5*	LSM5 homolog, U6 small nuclear RNA associated (S. cerevisiae)	0.421	4.9E-02
213491_x_at	*RPN2*	ribophorin II	0.421	4.9E-02
224387_at	*COMMD5*	COMM domain containing 5	0.421	4.9E-02
225643_at	*MAPK1IP1L*	mitogen-activated protein kinase 1 interacting protein 1-like	0.421	4.9E-02
209451_at	*TANK*	TRAF family member-associated NFKB activator	0.420	4.9E-02
213007_at	*FANCI*	Fanconi anemia, complementation group I	0.420	5.0E-02
224428_s_at	*CDCA7*	cell division cycle associated 7	0.420	5.0E-02
201013_s_at	*PAICS*	phosphoribosylaminoimidazole carboxylase, phosphoribosylaminoimidazole succinocarboxamide synthetase	0.420	5.0E-02
242451_x_at	*RPS19*	ribosomal protein S19	0.420	5.0E-02
209035_at	*MDK*	midkine (neurite growth-promoting factor 2)	0.420	5.0E-02
209502_s_at	*BAIAP2*	BAI1-associated protein 2	0.420	5.0E-02
210959_s_at	*SRD5A1*	steroid-5-alpha-reductase, alpha polypeptide 1 (3-oxo-5 alpha-steroid delta 4-dehydrogenase alpha 1)	0.420	5.0E-02
213399_x_at	*RPN2*	ribophorin II	0.420	5.0E-02
211959_at	*IGFBP5*	insulin-like growth factor binding protein 5	0.419	5.0E-02
221732_at	*CANT1*	calcium activated nucleotidase 1	0.419	5.0E-02
202218_s_at	*FADS2*	fatty acid desaturase 2	-0.419	5.0E-02
222157_s_at	*WDR48*	WD repeat domain 48	-0.419	5.0E-02
226674_at	*SHISA4*	shisa homolog 4 (Xenopus laevis)	-0.419	5.0E-02
228333_at	*ZEB2*	zinc finger E-box binding homeobox 2	-0.419	5.0E-02
201813_s_at	*TBC1D5*	TBC1 domain family, member 5	-0.420	5.0E-02
37566_at	*KIAA1045*	KIAA1045	-0.420	5.0E-02
206401_s_at	*MAPT*	microtubule-associated protein tau	-0.420	5.0E-02
212534_at	*ZNF24*	zinc finger protein 24	-0.420	5.0E-02
222391_at	*TMEM30A*	transmembrane protein 30A	-0.420	5.0E-02

Probeset ID	Gene symbol	Gene description	ρ	Adjusted *P*-value
212416_at	*SCAMP1*	secretory carrier membrane protein 1	-0.420	5.0E-02
217722_s_at	*NGRN*	neugrin, neurite outgrowth associated	-0.420	5.0E-02
217970_s_at	*CNOT6*	CCR4-NOT transcription complex, subunit 6	-0.420	5.0E-02
213313_at	*RABGAP1*	RAB GTPase activating protein 1	-0.420	5.0E-02
217830_s_at	*NSFL1C*	NSFL1 (p97) cofactor (p47)	-0.420	5.0E-02
202873_at	*ATP6V1C1*	ATPase, H+ transporting, lysosomal 42kDa, V1 subunit C1	-0.420	5.0E-02
219009_at	*C14orf93*	chromosome 14 open reading frame 93	-0.420	4.9E-02
222586_s_at	*OSBPL11*	oxysterol binding protein-like 11	-0.420	4.9E-02
233070_at	*ZNF197*	zinc finger protein 197	-0.420	4.9E-02
215606_s_at	*ERC1*	ELKS/RAB6-interacting/CAST family member 1	-0.420	4.9E-02
221622_s_at	*TMEM126B*	transmembrane protein 126B	-0.420	4.9E-02
222717_at	*SDPR*	serum deprivation response	-0.420	4.9E-02
235905_at	*ZNF704*	zinc finger protein 704	-0.420	4.9E-02
220755_s_at	*C6orf48*	chromosome 6 open reading frame 48	-0.421	4.9E-02
243904_at	*STXBP5*	syntaxin binding protein 5 (tomosyn)	-0.421	4.9E-02
1556021_at	*GPR180*	G protein-coupled receptor 180	-0.421	4.9E-02
212286_at	*ANKRD12*	ankyrin repeat domain 12	-0.421	4.9E-02
215115_x_at	*NTRK3*	neurotrophic tyrosine kinase, receptor, type 3	-0.421	4.9E-02
235475_at	*SERP1*	stress-associated endoplasmic reticulum protein 1	-0.421	4.9E-02
203081_at	*CTNNBIP1*	catenin, beta interacting protein 1	-0.421	4.9E-02
215307_at	*ZNF529*	zinc finger protein 529	-0.421	4.9E-02
217833_at	*SYNCRIP*	synaptotagmin binding, cytoplasmic RNA interacting protein	-0.421	4.9E-02
223099_s_at	*LONP2*	lon peptidase 2, peroxisomal	-0.421	4.9E-02
1558010_s_at	*SLC1A2*	solute carrier family 1 (glial high affinity glutamate transporter), member 2	-0.421	4.9E-02
238029_s_at	*SLC16A14*	solute carrier family 16, member 14 (monocarboxylic acid transporter 14)	-0.421	4.9E-02
209159_s_at	*NDRG4*	NDRG family member 4	-0.421	4.9E-02
212602_at	*WDFY3*	WD repeat and FYVE domain containing 3	-0.421	4.9E-02
228225_at	*PEX2*	peroxisomal biogenesis factor 2	-0.421	4.9E-02
225163_at	*FRMD4A*	FERM domain containing 4A	-0.421	4.9E-02
202874_s_at	*ATP6V1C1*	ATPase, H+ transporting, lysosomal 42kDa, V1 subunit C1	-0.421	4.9E-02
217100_s_at	*UBXN7*	UBX domain protein 7	-0.421	4.9E-02
218733_at	*MSL2*	male-specific lethal 2 homolog (Drosophila)	-0.421	4.9E-02
235065_at	*FAM59A*	family with sequence similarity 59, member A	-0.421	4.9E-02
207135_at	*HTR2A*	5-hydroxytryptamine (serotonin) receptor 2A, G protein-coupled	-0.421	4.9E-02
243007_at	*TTC5*	tetratricopeptide repeat domain 5	-0.421	4.9E-02
238477_at	*KIF1C*	kinesin family member 1C	-0.422	4.9E-02
223337_at	*CWC27*	CWC27 spliceosome-associated protein homolog (S. cerevisiae)	-0.422	4.8E-02
210251_s_at	*RUFY3*	RUN and FYVE domain containing 3	-0.422	4.8E-02
205103_at	*C1orf61*	chromosome 1 open reading frame 61	-0.422	4.8E-02
242303_at	*NRG2*	neuregulin 2	-0.422	4.8E-02
212820_at	*DMXL2*	Dmx-like 2	-0.422	4.8E-02
225855_at	*EPB41L5*	erythrocyte membrane protein band 4.1 like 5	-0.422	4.8E-02
242006_at	*LCA5*	Leber congenital amaurosis 5	-0.422	4.8E-02

Probeset ID	Gene symbol	Gene description	ρ	Adjusted *P*-value
203485_at	*RTN1*	reticulon 1	-0.423	4.8E-02
207100_s_at	*VAMP1*	vesicle-associated membrane protein 1 (synaptobrevin 1)	-0.423	4.8E-02
210908_s_at	*PFDN5*	prefoldin subunit 5	-0.423	4.8E-02
211819_s_at	*SORBS1*	sorbin and SH3 domain containing 1	-0.423	4.8E-02
221908_at	*RNFT2*	ring finger protein, transmembrane 2	-0.423	4.8E-02
213825_at	*OLIG2*	oligodendrocyte lineage transcription factor 2	-0.423	4.8E-02
225820_at	*PHF17*	PHD finger protein 17	-0.423	4.8E-02
229038_at	*CWF19L1*	CWF19-like 1, cell cycle control (S. pombe)	-0.423	4.8E-02
243417_at	*ZADH2*	zinc binding alcohol dehydrogenase domain containing 2	-0.423	4.8E-02
201646_at	*SCARB2*	scavenger receptor class B, member 2	-0.423	4.8E-02
205903_s_at	*KCNN3*	potassium intermediate/small conductance calcium-activated channel, subfamily N, member 3	-0.423	4.8E-02
221656_s_at	*ARHGEF10L*	Rho guanine nucleotide exchange factor (GEF) 10-like	-0.423	4.8E-02
231650_s_at	*SEZ6L*	Seizure related 6 homolog (mouse)-like	-0.423	4.8E-02
1558009_at	*SLC1A2*	solute carrier family 1 (glial high affinity glutamate transporter), member 2	-0.423	4.8E-02
203537_at	*PRPSAP2*	phosphoribosyl pyrophosphate synthetase-associated protein 2	-0.423	4.8E-02
1558675_s_at	*NEMF*	nuclear export mediator factor	-0.423	4.8E-02
211033_s_at	*PEX7*	peroxisomal biogenesis factor 7	-0.423	4.8E-02
1555154_a_at	*QKI*	QKI, KH domain containing, RNA binding	-0.424	4.8E-02
219732_at	*LPPR1*	lipid phosphate phosphatase-related protein type 1	-0.424	4.8E-02
235348_at	*ABHD13*	abhydrolase domain containing 13	-0.424	4.8E-02
224616_at	*DYNC1LI2*	dynein, cytoplasmic 1, light intermediate chain 2	-0.424	4.8E-02
235021_at	*KIAA2026*	KIAA2026	-0.424	4.8E-02
239442_at	*CEP68*	centrosomal protein 68kDa	-0.424	4.8E-02
218706_s_at	*GRAMD3*	GRAM domain containing 3	-0.424	4.7E-02
228822_s_at	*USP16*	ubiquitin specific peptidase 16	-0.424	4.7E-02
208925_at	*CLDND1*	claudin domain containing 1	-0.424	4.7E-02
214552_s_at	*RABEP1*	rabaptin, RAB GTPase binding effector protein 1	-0.424	4.7E-02
225856_at	*CLOCK*	clock homolog (mouse)	-0.424	4.7E-02
201226_at	*NDUFB8*	NADH dehydrogenase (ubiquinone) 1 beta subcomplex, 8, 19kDa	-0.424	4.7E-02
204832_s_at	*BMPR1A*	bone morphogenetic protein receptor, type IA	-0.424	4.7E-02
227435_at	*KIAA2018*	KIAA2018	-0.424	4.7E-02
227859_at	*DNAJC27*	DnaJ (Hsp40) homolog, subfamily C, member 27	-0.424	4.7E-02
203087_s_at	*KIF2A*	kinesin heavy chain member 2A	-0.424	4.7E-02
209684_at	*RIN2*	Ras and Rab interactor 2	-0.424	4.7E-02
209987_s_at	*ASCL1*	achaete-scute complex homolog 1 (Drosophila)	-0.424	4.7E-02
213848_at	*DUSP7*	dual specificity phosphatase 7	-0.424	4.7E-02
224769_at	*TAOK1*	TAO kinase 1	-0.424	4.7E-02
238441_at	*PRKAA2*	protein kinase, AMP-activated, alpha 2 catalytic subunit	-0.425	4.7E-02
215233_at	*JMJD6*	jumonji domain containing 6	-0.425	4.7E-02
221490_at	*UBAP1*	ubiquitin associated protein 1	-0.425	4.7E-02
212230_at	*PPAP2B*	phosphatidic acid phosphatase type 2B	-0.425	4.7E-02
223446_s_at	*DTNBP1*	dystrobrevin binding protein 1	-0.425	4.7E-02
227808_at	*DNAJC15*	DnaJ (Hsp40) homolog, subfamily C, member 15	-0.425	4.7E-02
208920_at	*SRI*	sorcin	-0.425	4.7E-02
225379_at	*MAPT*	microtubule-associated protein tau	-0.425	4.7E-02

Probeset ID	Gene symbol	Gene description	ρ	Adjusted *P*-value
231103_at	*KCNN3*	potassium intermediate/small conductance calcium-activated channel, subfamily N, member 3	-0.425	4.7E-02
202045_s_at	*ARHGAP35*	Rho GTPase activating protein 35	-0.426	4.6E-02
218946_at	*NFU1*	NFU1 iron-sulfur cluster scaffold homolog (S. cerevisiae)	-0.426	4.6E-02
208870_x_at	*ATP5C1*	ATP synthase, H+ transporting, mitochondrial F1 complex, gamma polypeptide 1	-0.426	4.6E-02
239897_at	*BCLAF1*	BCL2-associated transcription factor 1	-0.426	4.6E-02
206085_s_at	*CTH*	cystathionase (cystathionine gamma-lyase)	-0.426	4.6E-02
226230_at	*SMEK2*	SMEK homolog 2, suppressor of mek1 (Dictyostelium)	-0.426	4.6E-02
203972_s_at	*PEX3*	peroxisomal biogenesis factor 3	-0.426	4.6E-02
212894_at	*SUPV3L1*	suppressor of var1, 3-like 1 (S. cerevisiae)	-0.426	4.6E-02
224931_at	*SLC41A3*	solute carrier family 41, member 3	-0.426	4.6E-02
226277_at	*COL4A3BP*	collagen, type IV, alpha 3 (Goodpasture antigen) binding protein	-0.426	4.6E-02
55692_at	*ELMO2*	engulfment and cell motility 2	-0.426	4.6E-02
201158_at	*NMT1*	N-myristoyltransferase 1	-0.426	4.6E-02
225475_at	*MIER1*	mesoderm induction early response 1 homolog (Xenopus laevis)	-0.426	4.6E-02
210202_s_at	*BIN1*	bridging integrator 1	-0.427	4.6E-02
209610_s_at	*SLC1A4*	solute carrier family 1 (glutamate/neutral amino acid transporter), member 4	-0.427	4.6E-02
210600_s_at	*GRK4*	G protein-coupled receptor kinase 4	-0.427	4.6E-02
223213_s_at	*ZHX1*	zinc fingers and homeoboxes 1	-0.427	4.6E-02
212669_at	*CAMK2G*	calcium/calmodulin-dependent protein kinase II gamma	-0.427	4.6E-02
203298_s_at	*JARID2*	jumonji, AT rich interactive domain 2	-0.427	4.6E-02
210222_s_at	*RTN1*	reticulon 1	-0.427	4.6E-02
242443_at	*EML5*	Echinoderm microtubule associated protein like 5	-0.427	4.6E-02
203377_s_at	*CDC40*	cell division cycle 40 homolog (S. cerevisiae)	-0.427	4.5E-02
228817_at	*ALG9*	asparagine-linked glycosylation 9, alpha-1,2-mannosyltransferase homolog (S. cerevisiae)	-0.427	4.5E-02
1568777_at	*EML5*	echinoderm microtubule associated protein like 5	-0.427	4.5E-02
203266_s_at	*MAP2K4*	mitogen-activated protein kinase kinase 4	-0.427	4.5E-02
241966_at	*MYO5A*	myosin VA (heavy chain 12, myoxin)	-0.427	4.5E-02
223230_at	*PRPF38A*	PRP38 pre-mRNA processing factor 38 (yeast) domain containing A	-0.427	4.5E-02
230183_at	*EXT1*	exostosin 1	-0.427	4.5E-02
207785_s_at	*RBPJ*	recombination signal binding protein for immunoglobulin kappa J region	-0.427	4.5E-02
200694_s_at	*DDX24*	DEAD (Asp-Glu-Ala-Asp) box polypeptide 24	-0.428	4.5E-02
208398_s_at	*TBPL1*	TBP-like 1	-0.428	4.5E-02
209683_at	*FAM49A*	family with sequence similarity 49, member A	-0.428	4.5E-02
212672_at	*ATM*	ataxia telangiectasia mutated	-0.428	4.5E-02
57588_at	*SLC24A3*	solute carrier family 24 (sodium/potassium/calcium exchanger), member 3	-0.428	4.5E-02
209075_s_at	*ISCU*	iron-sulfur cluster scaffold homolog (E. coli)	-0.428	4.5E-02
209724_s_at	*ZFP161*	zinc finger protein 161 homolog (mouse)	-0.428	4.5E-02
225041_at	*MPHOSPH8*	M-phase phosphoprotein 8	-0.428	4.5E-02
233555_s_at	*SULF2*	sulfatase 2	-0.428	4.5E-02
232166_at	*KIAA1377*	KIAA1377	-0.428	4.5E-02
217033_x_at	*NTRK3*	neurotrophic tyrosine kinase, receptor, type 3	-0.429	4.5E-02

Probeset ID	Gene symbol	Gene description	ρ	Adjusted *P*-value
228214_at	*SOX6*	SRY (sex determining region Y)-box 6	-0.429	4.5E-02
208070_s_at	*REV3L*	REV3-like, polymerase (DNA directed), zeta, catalytic subunit	-0.429	4.5E-02
227111_at	*ZBTB34*	zinc finger and BTB domain containing 34	-0.429	4.5E-02
1566257_at	*GPR180*	G protein-coupled receptor 180	-0.429	4.4E-02
220596_at	*GPATCH4*	G patch domain containing 4	-0.429	4.4E-02
201986_at	*MED13*	mediator complex subunit 13	-0.429	4.4E-02
210738_s_at	*SLC4A4*	solute carrier family 4, sodium bicarbonate cotransporter, member 4	-0.429	4.4E-02
222447_at	*METTL9*	methyltransferase like 9	-0.429	4.4E-02
209798_at	*NPAT*	nuclear protein, ataxia-telangiectasia locus	-0.429	4.4E-02
204716_at	*CCDC6*	coiled-coil domain containing 6	-0.429	4.4E-02
236126_at	*ACVR2B*	activin A receptor, type IIB	-0.429	4.4E-02
240236_at	*STXBP5L*	syntaxin binding protein 5-like	-0.429	4.4E-02
221104_s_at	*NIPSNAP3B*	nipsnap homolog 3B (C. elegans)	-0.430	4.4E-02
221311_x_at	*LYRM2*	LYR motif containing 2	-0.430	4.4E-02
224728_at	*ATPAF1*	ATP synthase mitochondrial F1 complex assembly factor 1	-0.430	4.4E-02
227064_at	*ANKRD40*	ankyrin repeat domain 40	-0.430	4.4E-02
204226_at	*STAU2*	staufen, RNA binding protein, homolog 2 (Drosophila)	-0.430	4.4E-02
1552752_a_at	*CADM2*	cell adhesion molecule 2	-0.430	4.4E-02
205050_s_at	*MAPK8IP2*	mitogen-activated protein kinase 8 interacting protein 2	-0.430	4.4E-02
240106_at	*GNPTAB*	N-acetylglucosamine-1-phosphate transferase, alpha and beta subunits	-0.430	4.4E-02
219093_at	*PID1*	phosphotyrosine interaction domain containing 1	-0.430	4.4E-02
204303_s_at	*CTIF*	CBP80/20-dependent translation initiation factor	-0.430	4.3E-02
205846_at	*PTPRB*	protein tyrosine phosphatase, receptor type, B	-0.430	4.3E-02
213993_at	*SPON1*	spondin 1, extracellular matrix protein	-0.431	4.3E-02
204824_at	*ENDOG*	endonuclease G	-0.431	4.3E-02
210053_at	*TAF5*	TAF5 RNA polymerase II, TATA box binding protein (TBP)-associated factor, 100kDa	-0.431	4.3E-02
201490_s_at	*PPIF*	peptidylprolyl isomerase F	-0.431	4.3E-02
235331_x_at	*PCGF5*	polycomb group ring finger 5	-0.431	4.3E-02
217821_s_at	*WBP11*	WW domain binding protein 11	-0.431	4.3E-02
218304_s_at	*OSBPL11*	oxysterol binding protein-like 11	-0.431	4.3E-02
218428_s_at	*REV1*	REV1, polymerase (DNA directed)	-0.431	4.3E-02
212607_at	*AKT3*	v-akt murine thymoma viral oncogene homolog 3 (protein kinase B, gamma)	-0.431	4.3E-02
230869_at	*FAM155A*	family with sequence similarity 155, member A	-0.431	4.3E-02
1558075_at	*LOC399491*	GPS, PLAT and transmembrane domain-containing protein	-0.431	4.3E-02
226095_s_at	*ATXN1L*	ataxin 1-like	-0.431	4.3E-02
242088_at	*KLHL24*	kelch-like 24 (Drosophila)	-0.431	4.3E-02
226918_at	*JPH4*	junctophilin 4	-0.432	4.3E-02
243735_at	*ELP2*	elongation protein 2 homolog (S. cerevisiae)	-0.432	4.3E-02
225698_at	*EPB41L4A-AS1*	EPB41L4A antisense RNA 1 (non-protein coding)	-0.432	4.3E-02
226842_at	*FBXL20*	F-box and leucine-rich repeat protein 20	-0.432	4.3E-02
223227_at	*BBS2*	Bardet-Biedl syndrome 2	-0.432	4.3E-02
228291_s_at	*PLK1S1*	polo-like kinase 1 substrate 1	-0.432	4.3E-02
201214_s_at	*PPP1R7*	protein phosphatase 1, regulatory subunit 7	-0.432	4.3E-02

Probeset ID	Gene symbol	Gene description	ρ	Adjusted *P*-value
209005_at	*FBXL5*	F-box and leucine-rich repeat protein 5	-0.432	4.3E-02
235018_at	*CSRNP3*	cysteine-serine-rich nuclear protein 3	-0.432	4.3E-02
235626_at	*CAMK1D*	calcium/calmodulin-dependent protein kinase ID	-0.432	4.3E-02
209142_s_at	*UBE2G1*	ubiquitin-conjugating enzyme E2G 1	-0.432	4.2E-02
219421_at	*TTC33*	tetratricopeptide repeat domain 33	-0.432	4.2E-02
232382_s_at	*PCMTD1*	protein-L-isoaspartate (D-aspartate) O-methyltransferase domain containing 1	-0.432	4.2E-02
225175_s_at	*SLC44A2*	solute carrier family 44, member 2	-0.432	4.2E-02
226198_at	*TOM1L2*	target of myb1-like 2 (chicken)	-0.432	4.2E-02
216037_x_at	*TCF7L2*	transcription factor 7-like 2 (T-cell specific, HMG-box)	-0.432	4.2E-02
227340_s_at	*RGMB*	RGM domain family, member B	-0.433	4.2E-02
215707_s_at	*PRNP*	prion protein	-0.433	4.2E-02
1554424_at	*FIP1L1*	FIP1 like 1 (S. cerevisiae)	-0.433	4.2E-02
229428_at	*TIMM23*	translocase of inner mitochondrial membrane 23 homolog (yeast)	-0.433	4.2E-02
212835_at	*FAM175B*	family with sequence similarity 175, member B	-0.433	4.2E-02
225603_s_at	*TRIQK*	triple QxxK/R motif containing	-0.434	4.2E-02
1554486_a_at	*GFOD1*	glucose-fructose oxidoreductase domain containing 1	-0.434	4.2E-02
202429_s_at	*PPP3CA*	protein phosphatase 3, catalytic subunit, alpha isozyme	-0.434	4.2E-02
203463_s_at	*EPN2*	epsin 2	-0.434	4.2E-02
214118_x_at	*PCM1*	pericentriolar material 1	-0.434	4.2E-02
230288_at	*FGF14*	fibroblast growth factor 14	-0.434	4.1E-02
213480_at	*VAMP4*	vesicle-associated membrane protein 4	-0.434	4.1E-02
218491_s_at	*THYN1*	thymocyte nuclear protein 1	-0.434	4.1E-02
227657_at	*RNF150*	ring finger protein 150	-0.434	4.1E-02
231959_at	*LIN52*	lin-52 homolog (C. elegans)	-0.434	4.1E-02
218135_at	*ERGIC2*	ERGIC and golgi 2	-0.435	4.1E-02
225678_at	*POLR3H*	polymerase (RNA) III (DNA directed) polypeptide H (22.9kD)	-0.435	4.1E-02
227422_at	*STRN*	striatin, calmodulin binding protein	-0.435	4.1E-02
232244_at	*KIAA1161*	KIAA1161	-0.435	4.1E-02
201992_s_at	*KIF5B*	kinesin family member 5B	-0.435	4.1E-02
231810_at	*BRI3BP*	BRI3 binding protein	-0.435	4.1E-02
231969_at	*STOX2*	storkhead box 2	-0.435	4.1E-02
203635_at	*DSCR3*	Down syndrome critical region gene 3	-0.435	4.1E-02
235918_x_at	*CEP97*	centrosomal protein 97kDa	-0.435	4.1E-02
241372_at	*ZC3H6*	zinc finger CCCH-type containing 6	-0.435	4.1E-02
223595_at	*TMEM133*	transmembrane protein 133	-0.435	4.1E-02
226046_at	*MAPK8*	mitogen-activated protein kinase 8	-0.435	4.1E-02
209558_s_at	*HIP1R*	huntingtin interacting protein 1 related	-0.435	4.1E-02
215201_at	*REPS1*	RALBP1 associated Eps domain containing 1	-0.435	4.1E-02
230078_at	*RAPGEF6*	Rap guanine nucleotide exchange factor (GEF) 6	-0.435	4.1E-02
243507_s_at	*C20orf196*	chromosome 20 open reading frame 196	-0.435	4.1E-02
206992_s_at	*ATP5S*	ATP synthase, H+ transporting, mitochondrial Fo complex, subunit s (factor B)	-0.436	4.1E-02
212771_at	*FAM171A1*	family with sequence similarity 171, member A1	-0.436	4.1E-02
217973_at	*DCXR*	dicarbonyl/L-xylulose reductase	-0.436	4.1E-02
203544_s_at	*STAM*	signal transducing adaptor molecule (SH3 domain and ITAM motif) 1	-0.436	4.1E-02

Probeset ID	Gene symbol	Gene description	ρ	Adjusted *P*-value
205712_at	*PTPRD*	protein tyrosine phosphatase, receptor type, D	-0.436	4.0E-02
223661_at	*NUCKS1*	Nuclear casein kinase and cyclin-dependent kinase substrate 1	-0.436	4.0E-02
224281_s_at	*NGRN*	neugrin, neurite outgrowth associated	-0.436	4.0E-02
200816_s_at	*PAFAH1B1*	platelet-activating factor acetylhydrolase 1b, regulatory subunit 1 (45kDa)	-0.436	4.0E-02
208667_s_at	*ST13*	suppression of tumorigenicity 13 (colon carcinoma) (Hsp70 interacting protein)	-0.436	4.0E-02
227701_at	*C10orf118*	chromosome 10 open reading frame 118	-0.436	4.0E-02
1553227_s_at	*BRWD1*	bromodomain and WD repeat domain containing 1	-0.436	4.0E-02
203075_at	*SMAD2*	SMAD family member 2	-0.436	4.0E-02
228122_at	*CCDC66*	coiled-coil domain containing 66	-0.436	4.0E-02
235672_at	*MAP6*	microtubule-associated protein 6	-0.436	4.0E-02
205022_s_at	*FOXN3*	forkhead box N3	-0.436	4.0E-02
202167_s_at	*MMS19*	MMS19 nucleotide excision repair homolog (S. cerevisiae)	-0.436	4.0E-02
207308_at	*SLCO1A2*	solute carrier organic anion transporter family, member 1A2	-0.436	4.0E-02
212114_at	*ATXN7L3B*	ataxin 7-like 3B	-0.437	4.0E-02
228196_s_at	*LARP4B*	La ribonucleoprotein domain family, member 4B	-0.437	4.0E-02
213805_at	*ABHD5*	abhydrolase domain containing 5	-0.437	4.0E-02
225409_at	*COA5*	cytochrome C oxidase assembly factor 5	-0.437	4.0E-02
201164_s_at	*PUM1*	pumilio homolog 1 (Drosophila)	-0.437	4.0E-02
206179_s_at	*TPPP*	tubulin polymerization promoting protein	-0.437	4.0E-02
214590_s_at	*UBE2D1*	ubiquitin-conjugating enzyme E2D 1	-0.437	4.0E-02
225031_at	*CHD6*	chromodomain helicase DNA binding protein 6	-0.437	4.0E-02
219077_s_at	*WWOX*	WW domain containing oxidoreductase	-0.437	4.0E-02
238585_at	*GTDC1*	glycosyltransferase-like domain containing 1	-0.437	4.0E-02
202986_at	*ARNT2*	aryl-hydrocarbon receptor nuclear translocator 2	-0.437	4.0E-02
223912_s_at	*CLN8*	ceroid-lipofuscinosis, neuronal 8 (epilepsy, progressive with mental retardation)	-0.437	4.0E-02
213106_at	*ATP8A1*	ATPase, aminophospholipid transporter (APLT), class I, type 8A, member 1	-0.437	4.0E-02
229679_at	*C12orf76*	chromosome 12 open reading frame 76	-0.437	4.0E-02
203080_s_at	*BAZ2B*	bromodomain adjacent to zinc finger domain, 2B	-0.437	4.0E-02
209323_at	*PRKRIR*	protein-kinase, interferon-inducible double stranded RNA dependent inhibitor, repressor of (P58 repressor)	-0.437	4.0E-02
242586_at	*FSD1L*	fibronectin type III and SPRY domain containing 1-like	-0.438	4.0E-02
206308_at	*TRDMT1*	tRNA aspartic acid methyltransferase 1	-0.438	4.0E-02
211081_s_at	*MAP4K5*	mitogen-activated protein kinase kinase kinase kinase 5	-0.438	4.0E-02
225018_at	*SPIRE1*	spire homolog 1 (Drosophila)	-0.438	4.0E-02
225192_at	*CACUL1*	CDK2-associated, cullin domain 1	-0.438	4.0E-02
204091_at	*PDE6D*	phosphodiesterase 6D, cGMP-specific, rod, delta	-0.438	4.0E-02
222469_s_at	*TOLLIP*	toll interacting protein	-0.438	4.0E-02
208714_at	*NDUFV1*	NADH dehydrogenase (ubiquinone) flavoprotein 1, 51kDa	-0.438	4.0E-02
39582_at	*CYLD*	cylindromatosis (turban tumor syndrome)	-0.438	4.0E-02
237515_at	*TMEM56*	transmembrane protein 56	-0.438	3.9E-02
210835_s_at	*CTBP2*	C-terminal binding protein 2	-0.438	3.9E-02
230933_at	*DSTN*	Destrin (actin depolymerizing factor)	-0.438	3.9E-02

Probeset ID	Gene symbol	Gene description	ρ	Adjusted *P*-value
204019_s_at	*SH3YL1*	SH3 domain containing, Ysc84-like 1 (S. cerevisiae)	-0.439	3.9E-02
226649_at	*PANK1*	pantothenate kinase 1	-0.439	3.9E-02
209497_s_at	*RBM4B*	RNA binding motif protein 4B	-0.439	3.9E-02
222460_s_at	*FAM192A*	family with sequence similarity 192, member A	-0.439	3.9E-02
232038_at	*C6orf170*	chromosome 6 open reading frame 170	-0.439	3.9E-02
232020_at	*SMURF2*	SMAD specific E3 ubiquitin protein ligase 2	-0.439	3.9E-02
222434_at	*ENAH*	enabled homolog (Drosophila)	-0.439	3.9E-02
203304_at	*BAMBI*	BMP and activin membrane-bound inhibitor homolog (Xenopus laevis)	-0.439	3.9E-02
225045_at	*CCDC88A*	coiled-coil domain containing 88A	-0.439	3.9E-02
227498_at	*SOX6*	SRY (sex determining region Y)-box 6	-0.439	3.9E-02
227608_at	*DAP3*	death associated protein 3	-0.439	3.9E-02
228266_s_at	*HDGFRP3*	hepatoma-derived growth factor, related protein 3	-0.439	3.9E-02
201719_s_at	*EPB41L2*	erythrocyte membrane protein band 4.1-like 2	-0.439	3.9E-02
203494_s_at	*CEP57*	centrosomal protein 57kDa	-0.439	3.9E-02
218841_at	*ASB8*	ankyrin repeat and SOCS box containing 8	-0.439	3.9E-02
219889_at	*FRAT1*	frequently rearranged in advanced T-cell lymphomas	-0.440	3.8E-02
203553_s_at	*MAP4K5*	mitogen-activated protein kinase kinase kinase kinase 5	-0.440	3.8E-02
213199_at	*C2CD3*	C2 calcium-dependent domain containing 3	-0.440	3.8E-02
200832_s_at	*SCD*	stearoyl-CoA desaturase (delta-9-desaturase)	-0.440	3.8E-02
213049_at	*RALGAPA1*	Ral GTPase activating protein, alpha subunit 1 (catalytic)	-0.440	3.8E-02
217952_x_at	*PHF3*	PHD finger protein 3	-0.440	3.8E-02
218111_s_at	*CMAS*	cytidine monophosphate N-acetylneuraminic acid synthetase	-0.440	3.8E-02
220321_s_at	*CCDC121*	coiled-coil domain containing 121	-0.440	3.8E-02
201235_s_at	*BTG2*	BTG family, member 2	-0.440	3.8E-02
204009_s_at	*KRAS*	v-Ki-ras2 Kirsten rat sarcoma viral oncogene homolog	-0.441	3.8E-02
33494_at	*ETFDH*	electron-transferring-flavoprotein dehydrogenase	-0.441	3.8E-02
204434_at	*SPATA2*	spermatogenesis associated 2	-0.441	3.8E-02
223437_at	*PPARA*	peroxisome proliferator-activated receptor alpha	-0.441	3.8E-02
219799_s_at	*DHRS9*	dehydrogenase/reductase (SDR family) member 9	-0.441	3.8E-02
227261_at	*KLF12*	Kruppel-like factor 12	-0.441	3.8E-02
233985_x_at	*PPP1R9A*	protein phosphatase 1, regulatory subunit 9A	-0.441	3.8E-02
230276_at	*FAM49A*	family with sequence similarity 49, member A	-0.441	3.8E-02
1554807_a_at	*SPIRE1*	spire homolog 1 (Drosophila)	-0.441	3.8E-02
226312_at	*RICTOR*	RPTOR independent companion of MTOR, complex 2	-0.441	3.8E-02
218137_s_at	*SMAP1*	small ArfGAP 1	-0.441	3.8E-02
208731_at	*RAB2A*	RAB2A, member RAS oncogene family	-0.441	3.8E-02
208733_at	*RAB2A*	RAB2A, member RAS oncogene family	-0.441	3.8E-02
1554470_s_at	*ZBTB44*	zinc finger and BTB domain containing 44	-0.442	3.7E-02
240859_at	*ZFYVE16*	zinc finger, FYVE domain containing 16	-0.442	3.7E-02
230759_at	*SNX14*	Sorting nexin 14	-0.442	3.7E-02
201348_at	*GPX3*	glutathione peroxidase 3 (plasma)	-0.442	3.7E-02
220277_at	*CXXC4*	CXXC finger protein 4	-0.442	3.7E-02
226484_at	*ZBTB47*	zinc finger and BTB domain containing 47	-0.443	3.7E-02
241701_at	*ARHGAP21*	Rho GTPase activating protein 21	-0.443	3.7E-02
226269_at	*GDAP1*	ganglioside induced differentiation associated protein 1	-0.443	3.7E-02
218386_x_at	*USP16*	ubiquitin specific peptidase 16	-0.443	3.7E-02
206374_at	*DUSP8*	dual specificity phosphatase 8	-0.443	3.6E-02

Probeset ID	Gene symbol	Gene description	ρ	Adjusted *P*-value
212829_at	*PIP4K2A*	phosphatidylinositol-5-phosphate 4-kinase, type II, alpha	-0.443	3.6E-02
210136_at	*MBP*	myelin basic protein	-0.444	3.6E-02
213355_at	*ST3GAL6*	ST3 beta-galactoside alpha-2,3-sialyltransferase 6	-0.444	3.6E-02
217895_at	*PTCD3*	pentatricopeptide repeat domain 3	-0.444	3.6E-02
218087_s_at	*SORBS1*	sorbin and SH3 domain containing 1	-0.444	3.6E-02
219671_at	*HPCAL4*	hippocalcin like 4	-0.444	3.6E-02
241809_at	*FAM212B*	family with sequence similarity 212, member B	-0.444	3.6E-02
203598_s_at	*WBP4*	WW domain binding protein 4 (formin binding protein 21)	-0.444	3.6E-02
219341_at	*CLN8*	ceroid-lipofuscinosis, neuronal 8 (epilepsy, progressive with mental retardation)	-0.444	3.6E-02
221519_at	*FBXW4*	F-box and WD repeat domain containing 4	-0.444	3.6E-02
224782_at	*ZMAT2*	zinc finger, matrin-type 2	-0.444	3.6E-02
227037_at	*PLD6*	phospholipase D family, member 6	-0.444	3.6E-02
231567_s_at	*CCDC62*	coiled-coil domain containing 62	-0.444	3.6E-02
206462_s_at	*NTRK3*	neurotrophic tyrosine kinase, receptor, type 3	-0.444	3.6E-02
202521_at	*CTCF*	CCCTC-binding factor (zinc finger protein)	-0.444	3.6E-02
217936_at	*ARHGAP5*	Rho GTPase activating protein 5	-0.444	3.6E-02
218877_s_at	*TRMT11*	tRNA methyltransferase 11 homolog (S. cerevisiae)	-0.444	3.6E-02
227637_at	*TFCP2*	transcription factor CP2	-0.444	3.6E-02
235694_at	*TCFL5*	transcription factor-like 5 (basic helix-loop-helix)	-0.444	3.6E-02
200597_at	*EIF3A*	eukaryotic translation initiation factor 3, subunit A	-0.444	3.6E-02
218824_at	*PNMAL1*	paraneoplastic Ma antigen family-like 1	-0.445	3.6E-02
209452_s_at	*VTI1B*	vesicle transport through interaction with t-SNAREs homolog 1B (yeast)	-0.445	3.6E-02
213960_at	*NTRK3*	neurotrophic tyrosine kinase, receptor, type 3	-0.445	3.6E-02
1552627_a_at	*ARHGAP5*	Rho GTPase activating protein 5	-0.445	3.6E-02
205730_s_at	*ABLIM3*	actin binding LIM protein family, member 3	-0.445	3.6E-02
202260_s_at	*STXBP1*	syntaxin binding protein 1	-0.445	3.6E-02
224784_at	*MLLT6*	myeloid/lymphoid or mixed-lineage leukemia (trithorax homolog, Drosophila); translocated to, 6	-0.445	3.6E-02
218603_at	*HECA*	headcase homolog (Drosophila)	-0.445	3.6E-02
202187_s_at	*PPP2R5A*	protein phosphatase 2, regulatory subunit B', alpha	-0.445	3.6E-02
212830_at	*MEGF9*	multiple EGF-like-domains 9	-0.445	3.6E-02
202544_at	*GMFB*	glia maturation factor, beta	-0.445	3.6E-02
213407_at	*PHLPP2*	PH domain and leucine rich repeat protein phosphatase 2	-0.446	3.5E-02
228329_at	*DAB1*	disabled homolog 1 (Drosophila)	-0.446	3.5E-02
214585_s_at	*VPS52*	vacuolar protein sorting 52 homolog (S. cerevisiae)	-0.446	3.5E-02
216100_s_at	*TOR1AIP1*	torsin A interacting protein 1	-0.446	3.5E-02
203790_s_at	*HRSP12*	heat-responsive protein 12	-0.446	3.5E-02
227000_at	*C7orf41*	chromosome 7 open reading frame 41	-0.446	3.5E-02
201799_s_at	*OSBP*	oxysterol binding protein	-0.446	3.5E-02
203364_s_at	*ATG13*	autophagy related 13	-0.446	3.5E-02
213236_at	*SASH1*	SAM and SH3 domain containing 1	-0.446	3.5E-02
217969_at	*C11orf2*	chromosome 11 open reading frame 2	-0.446	3.5E-02
200596_s_at	*EIF3A*	eukaryotic translation initiation factor 3, subunit A	-0.446	3.5E-02
226398_s_at	*FRA10AC1*	fragile site, folic acid type, rare, fra(10)(q23.3) or fra(10)(q24.2) candidate 1	-0.446	3.5E-02
202770_s_at	*CCNG2*	cyclin G2	-0.446	3.5E-02

Probeset ID	Gene symbol	Gene description	ρ	Adjusted *P*-value
225629_s_at	*ZBTB4*	zinc finger and BTB domain containing 4	-0.446	3.5E-02
202359_s_at	*SNX19*	sorting nexin 19	-0.447	3.5E-02
218874_s_at	*ATAT1*	alpha tubulin acetyltransferase 1	-0.447	3.5E-02
206506_s_at	*SUPT3H*	suppressor of Ty 3 homolog (S. cerevisiae)	-0.447	3.5E-02
205651_x_at	*RAPGEF4*	Rap guanine nucleotide exchange factor (GEF) 4	-0.447	3.5E-02
209018_s_at	*PINK1*	PTEN induced putative kinase 1	-0.447	3.5E-02
204151_x_at	*AKR1C1*	aldo-keto reductase family 1, member C1 (dihydrodiol dehydrogenase 1; 20-alpha (3-alpha)-hydroxysteroid dehydrogenase)	-0.447	3.5E-02
226339_at	*TRUB1*	TruB pseudouridine (psi) synthase homolog 1 (E. coli)	-0.447	3.5E-02
1556696_s_at	*FLJ42709*	uncharacterized LOC441094	-0.447	3.5E-02
244011_at	*PPM1K*	protein phosphatase, Mg2+/Mn2+ dependent, 1K	-0.447	3.5E-02
207152_at	*NTRK2*	neurotrophic tyrosine kinase, receptor, type 2	-0.447	3.5E-02
222405_at	*PTPLAD1*	protein tyrosine phosphatase-like A domain containing 1	-0.448	3.5E-02
203628_at	*IGF1R*	insulin-like growth factor 1 receptor	-0.448	3.4E-02
228512_at	*PTCD3*	pentatricopeptide repeat domain 3	-0.448	3.4E-02
244194_at	*ADAM22*	ADAM metallopeptidase domain 22	-0.448	3.4E-02
218862_at	*ASB13*	ankyrin repeat and SOCS box containing 13	-0.448	3.4E-02
213278_at	*MTMR9*	myotubularin related protein 9	-0.448	3.4E-02
217644_s_at	*SOS2*	son of sevenless homolog 2 (Drosophila)	-0.448	3.4E-02
226572_at	*SOCS7*	suppressor of cytokine signaling 7	-0.448	3.4E-02
218185_s_at	*ARMC1*	armadillo repeat containing 1	-0.448	3.4E-02
208656_s_at	*CCNI*	cyclin I	-0.449	3.4E-02
218394_at	*ROGDI*	rogdi homolog (Drosophila)	-0.449	3.4E-02
213377_x_at	*RPS12*	ribosomal protein S12	-0.449	3.4E-02
227497_at	*SOX6*	SRY (sex determining region Y)-box 6	-0.449	3.4E-02
227624_at	*TET2*	tet methylcytosine dioxygenase 2	-0.449	3.4E-02
242418_at	*C2orf27A*	chromosome 2 open reading frame 27A	-0.449	3.4E-02
204672_s_at	*ANKRD6*	ankyrin repeat domain 6	-0.449	3.4E-02
225049_at	*BLOC1S2*	biogenesis of lysosomal organelles complex-1, subunit 2	-0.449	3.4E-02
222794_x_at	*MTPAP*	mitochondrial poly(A) polymerase	-0.449	3.4E-02
213626_at	*CBR4*	carbonyl reductase 4	-0.449	3.4E-02
229970_at	*KBTBD7*	kelch repeat and BTB (POZ) domain containing 7	-0.450	3.4E-02
244687_at	*DBT*	dihydrolipoamide branched chain transacylase E2	-0.450	3.3E-02
212339_at	*EPB41L1*	erythrocyte membrane protein band 4.1-like 1	-0.450	3.3E-02
243176_at	*ARL5A*	ADP-ribosylation factor-like 5A	-0.450	3.3E-02
211761_s_at	*CACYBP*	calcyclin binding protein	-0.450	3.3E-02
224564_s_at	*RTN3*	reticulon 3	-0.450	3.3E-02
226487_at	*FAM222A*	family with sequence similarity 222, member A	-0.450	3.3E-02
219067_s_at	*NSMCE4A*	non-SMC element 4 homolog A (S. cerevisiae)	-0.450	3.3E-02
205818_at	*DBC1*	deleted in bladder cancer 1	-0.450	3.3E-02
226169_at	*SBF2*	SET binding factor 2	-0.451	3.3E-02
206158_s_at	*CNBP*	CCHC-type zinc finger, nucleic acid binding protein	-0.451	3.3E-02
221788_at	*PGM3*	phosphoglucomutase 3	-0.451	3.3E-02
227370_at	*FAM171B*	family with sequence similarity 171, member B	-0.451	3.3E-02
203428_s_at	*ASF1A*	ASF1 anti-silencing function 1 homolog A (S. cerevisiae)	-0.451	3.3E-02
226139_at	*CCDC149*	coiled-coil domain containing 149	-0.452	3.2E-02
225623_at	*KIAA1737*	KIAA1737	-0.452	3.2E-02
221547_at	*PRPF18*	PRP18 pre-mRNA processing factor 18 homolog (S.	-0.452	3.2E-02

Probeset ID	Gene symbol	Gene description	ρ	Adjusted *P*-value
		cerevisiae)		
200662_s_at	*TOMM20*	translocase of outer mitochondrial membrane 20 homolog (yeast)	-0.452	3.2E-02
212153_at	*POGZ*	pogo transposable element with ZNF domain	-0.452	3.2E-02
218281_at	*MRPL48*	mitochondrial ribosomal protein L48	-0.452	3.2E-02
225477_s_at	*NR2C2*	nuclear receptor subfamily 2, group C, member 2	-0.452	3.2E-02
204512_at	*HIVEP1*	human immunodeficiency virus type I enhancer binding protein 1	-0.452	3.2E-02
209988_s_at	*ASCL1*	achaete-scute complex homolog 1 (Drosophila)	-0.452	3.2E-02
202363_at	*SPOCK1*	sparc/osteonectin, cwcv and kazal-like domains proteoglycan (testican) 1	-0.452	3.2E-02
212970_at	*APBB2*	amyloid beta (A4) precursor protein-binding, family B, member 2	-0.453	3.2E-02
224605_at	*C4orf3*	chromosome 4 open reading frame 3	-0.453	3.2E-02
227440_at	*ANKS1B*	ankyrin repeat and sterile alpha motif domain containing 1B	-0.453	3.2E-02
204823_at	*NAV3*	neuron navigator 3	-0.453	3.2E-02
204671_s_at	*ANKRD6*	ankyrin repeat domain 6	-0.453	3.1E-02
223799_at	*MSANTD4*	Myb/SANT-like DNA-binding domain containing 4 with coiled-coils	-0.453	3.1E-02
201959_s_at	*MYCBP2*	MYC binding protein 2, E3 ubiquitin protein ligase	-0.454	3.1E-02
214091_s_at	*GPX3*	glutathione peroxidase 3 (plasma)	-0.454	3.1E-02
229743_at	*ZNF438*	zinc finger protein 438	-0.454	3.1E-02
217777_s_at	*PTPLAD1*	protein tyrosine phosphatase-like A domain containing 1	-0.454	3.1E-02
221792_at	*RAB6B*	RAB6B, member RAS oncogene family	-0.454	3.1E-02
228457_at	*PPM1L*	protein phosphatase, Mg2+/Mn2+ dependent, 1L	-0.454	3.1E-02
238550_at	*RUFY2*	RUN and FYVE domain containing 2	-0.454	3.1E-02
204120_s_at	*ADK*	adenosine kinase	-0.454	3.1E-02
220282_at	*RIC3*	resistance to inhibitors of cholinesterase 3 homolog (C. elegans)	-0.454	3.1E-02
226425_at	*CLIP4*	CAP-GLY domain containing linker protein family, member 4	-0.454	3.1E-02
201381_x_at	*CACYBP*	calcyclin binding protein	-0.454	3.1E-02
206241_at	*KPNA5*	karyopherin alpha 5 (importin alpha 6)	-0.454	3.1E-02
207108_s_at	*NIPBL*	Nipped-B homolog (Drosophila)	-0.454	3.1E-02
210896_s_at	*ASPH*	aspartate beta-hydroxylase	-0.454	3.1E-02
220028_at	*ACVR2B*	activin A receptor, type IIB	-0.454	3.1E-02
208635_x_at	*NACA*	nascent polypeptide-associated complex alpha subunit	-0.454	3.1E-02
218523_at	*LHPP*	phospholysine phosphohistidine inorganic pyrophosphate phosphatase	-0.454	3.1E-02
227531_at	*CLOCK*	clock homolog (mouse)	-0.454	3.1E-02
209148_at	*RXRB*	retinoid X receptor, beta	-0.455	3.1E-02
1552754_a_at	*CADM2*	cell adhesion molecule 2	-0.455	3.1E-02
209160_at	*AKR1C3*	aldo-keto reductase family 1, member C3 (3-alpha hydroxysteroid dehydrogenase, type II)	-0.455	3.1E-02
241682_at	*KLHL23*	kelch-like 23 (Drosophila)	-0.455	3.1E-02
201051_at	*ANP32A*	acidic (leucine-rich) nuclear phosphoprotein 32 family, member A	-0.455	3.1E-02
219137_s_at	*MFF*	mitochondrial fission factor	-0.455	3.1E-02
209735_at	*ABCG2*	ATP-binding cassette, sub-family G (WHITE), member 2	-0.455	3.1E-02
200612_s_at	*AP2B1*	adaptor-related protein complex 2, beta 1 subunit	-0.455	3.1E-02

Probeset ID	Gene symbol	Gene description	ρ	Adjusted *P*-value
201220_x_at	*CTBP2*	C-terminal binding protein 2	-0.455	3.1E-02
226641_at	*ANKRD44*	ankyrin repeat domain 44	-0.455	3.1E-02
204238_s_at	*C6orf108*	chromosome 6 open reading frame 108	-0.456	3.1E-02
238923_at	*SPOP*	speckle-type POZ protein	-0.456	3.1E-02
224883_at	*PLDN*	pallidin homolog (mouse)	-0.456	3.0E-02
235472_at	*FUT10*	fucosyltransferase 10 (alpha (1,3) fucosyltransferase)	-0.456	3.0E-02
223350_x_at	*LIN7C*	lin-7 homolog C (C. elegans)	-0.456	3.0E-02
226399_at	*DNAJB14*	DnaJ (Hsp40) homolog, subfamily B, member 14	-0.456	3.0E-02
225339_at	*SPAG9*	sperm associated antigen 9	-0.456	3.0E-02
225218_at	*ZFYVE27*	zinc finger, FYVE domain containing 27	-0.457	3.0E-02
227119_at	*CNOT6L*	CCR4-NOT transcription complex, subunit 6-like	-0.457	3.0E-02
201915_at	*SEC63*	SEC63 homolog (S. cerevisiae)	-0.457	3.0E-02
201960_s_at	*MYCBP2*	MYC binding protein 2, E3 ubiquitin protein ligase	-0.457	3.0E-02
231175_at	*BEND6*	BEN domain containing 6	-0.457	3.0E-02
212774_at	*ZNF238*	zinc finger protein 238	-0.457	3.0E-02
227178_at	*CELF2*	CUGBP, Elav-like family member 2	-0.457	3.0E-02
212357_at	*FAM168A*	family with sequence similarity 168, member A	-0.457	3.0E-02
223263_s_at	*FGFR1OP2*	FGFR1 oncogene partner 2	-0.457	3.0E-02
238521_at	*FGF12*	fibroblast growth factor 12	-0.457	3.0E-02
1555268_a_at	*GRID1*	glutamate receptor, ionotropic, delta 1	-0.457	3.0E-02
218631_at	*AVPI1*	arginine vasopressin-induced 1	-0.457	3.0E-02
203223_at	*RABEP1*	rabaptin, RAB GTPase binding effector protein 1	-0.457	3.0E-02
218158_s_at	*APPL1*	adaptor protein, phosphotyrosine interaction, PH domain and leucine zipper containing 1	-0.457	3.0E-02
225198_at	*VAPA*	VAMP (vesicle-associated membrane protein)-associated protein A, 33kDa	-0.458	3.0E-02
203241_at	*UVRAG*	UV radiation resistance associated gene	-0.458	3.0E-02
202326_at	*EHMT2*	euchromatic histone-lysine N-methyltransferase 2	-0.458	3.0E-02
210121_at	*B3GALT2*	UDP-Gal:betaGlcNAc beta 1,3-galactosyltransferase, polypeptide 2	-0.458	3.0E-02
213439_x_at	*RUNDC3A*	RUN domain containing 3A	-0.458	3.0E-02
235415_at	*RPRD2*	regulation of nuclear pre-mRNA domain containing 2	-0.458	3.0E-02
227559_at	*NDUFAF4*	NADH dehydrogenase (ubiquinone) complex I, assembly factor 4	-0.458	3.0E-02
202923_s_at	*GCLC*	glutamate-cysteine ligase, catalytic subunit	-0.458	2.9E-02
244227_at	*SYT6*	synaptotagmin VI	-0.458	2.9E-02
201996_s_at	*SPEN*	spen homolog, transcriptional regulator (Drosophila)	-0.459	2.9E-02
203930_s_at	*MAPT*	microtubule-associated protein tau	-0.459	2.9E-02
206045_s_at	*NOL4*	nucleolar protein 4	-0.459	2.9E-02
221589_s_at	*ALDH6A1*	aldehyde dehydrogenase 6 family, member A1	-0.459	2.9E-02
239223_s_at	*FBXL20*	F-box and leucine-rich repeat protein 20	-0.459	2.9E-02
204546_at	*KIAA0513*	KIAA0513	-0.459	2.9E-02
205849_s_at	*UQCRB*	ubiquinol-cytochrome c reductase binding protein	-0.459	2.9E-02
226439_s_at	*NBEA*	neurobeachin	-0.459	2.9E-02
230141_at	*ARID4A*	AT rich interactive domain 4A (RBP1-like)	-0.459	2.9E-02
214434_at	*HSPA12A*	heat shock 70kDa protein 12A	-0.459	2.9E-02
217196_s_at	*CAMSAP2*	calmodulin regulated spectrin-associated protein family, member 2	-0.459	2.9E-02
201535_at	*UBL3*	ubiquitin-like 3	-0.459	2.9E-02

Probeset ID	Gene symbol	Gene description	ρ	Adjusted *P*-value
209274_s_at	*ISCA1*	iron-sulfur cluster assembly 1 homolog (S. cerevisiae)	-0.459	2.9E-02
209106_at	*NCOA1*	nuclear receptor coactivator 1	-0.460	2.9E-02
222880_at	*AKT3*	v-akt murine thymoma viral oncogene homolog 3 (protein kinase B, gamma)	-0.460	2.9E-02
225830_at	*PDZD8*	PDZ domain containing 8	-0.460	2.9E-02
201036_s_at	*HADH*	hydroxyacyl-CoA dehydrogenase	-0.460	2.9E-02
224458_at	*TMEM246*	transmembrane protein 246	-0.460	2.9E-02
209272_at	*NAB1*	NGFI-A binding protein 1 (EGR1 binding protein 1)	-0.460	2.9E-02
1552658_a_at	*NAV3*	neuron navigator 3	-0.460	2.9E-02
221820_s_at	*KAT8*	K(lysine) acetyltransferase 8	-0.460	2.9E-02
238476_at	*CREBRF*	CREB3 regulatory factor	-0.460	2.9E-02
200721_s_at	*ACTR1A*	ARP1 actin-related protein 1 homolog A, centractin alpha (yeast)	-0.460	2.9E-02
228793_at	*JMJD1C*	jumonji domain containing 1C	-0.460	2.9E-02
201962_s_at	*RNF41*	ring finger protein 41	-0.460	2.9E-02
210695_s_at	*WWOX*	WW domain containing oxidoreductase	-0.460	2.9E-02
219036_at	*CEP70*	centrosomal protein 70kDa	-0.460	2.9E-02
222538_s_at	*APPL1*	adaptor protein, phosphotyrosine interaction, PH domain and leucine zipper containing 1	-0.460	2.9E-02
244133_at	*USP49*	ubiquitin specific peptidase 49	-0.461	2.9E-02
221495_s_at	*TCF25*	transcription factor 25 (basic helix-loop-helix)	-0.461	2.9E-02
205005_s_at	*NMT2*	N-myristoyltransferase 2	-0.461	2.8E-02
212450_at	*SECISBP2L*	SECIS binding protein 2-like	-0.461	2.8E-02
218224_at	*PNMA1*	paraneoplastic Ma antigen 1	-0.461	2.8E-02
220595_at	*PDZRN4*	PDZ domain containing ring finger 4	-0.461	2.8E-02
222824_at	*NUDT5*	nudix (nucleoside diphosphate linked moiety X)-type motif 5	-0.461	2.8E-02
205171_at	*PTPN4*	protein tyrosine phosphatase, non-receptor type 4 (megakaryocyte)	-0.461	2.8E-02
213689_x_at	*FAM69A*	family with sequence similarity 69, member A	-0.461	2.8E-02
201989_s_at	*CREBL2*	cAMP responsive element binding protein-like 2	-0.461	2.8E-02
208166_at	*MMP16*	matrix metallopeptidase 16 (membrane-inserted)	-0.461	2.8E-02
224772_at	*NAV1*	neuron navigator 1	-0.461	2.8E-02
225092_at	*RABEP1*	rabaptin, RAB GTPase binding effector protein 1	-0.461	2.8E-02
1555544_a_at	*CADM2*	cell adhesion molecule 2	-0.461	2.8E-02
206385_s_at	*ANK3*	ankyrin 3, node of Ranvier (ankyrin G)	-0.461	2.8E-02
219728_at	*MYOT*	myotilin	-0.462	2.8E-02
230027_s_at	*MRPL43*	mitochondrial ribosomal protein L43	-0.462	2.8E-02
230002_at	*GPSM2*	G-protein signaling modulator 2	-0.462	2.8E-02
203482_at	*FAM178A*	family with sequence similarity 178, member A	-0.462	2.8E-02
222476_at	*CNOT6*	CCR4-NOT transcription complex, subunit 6	-0.462	2.8E-02
228771_at	*ADRBK2*	adrenergic, beta, receptor kinase 2	-0.462	2.8E-02
201976_s_at	*MYO10*	myosin X	-0.462	2.8E-02
225976_at	*BTF3L4*	basic transcription factor 3-like 4	-0.462	2.8E-02
226822_at	*STOX2*	storkhead box 2	-0.462	2.8E-02
212358_at	*CLIP3*	CAP-GLY domain containing linker protein 3	-0.463	2.7E-02
201772_at	*AZIN1*	antizyme inhibitor 1	-0.463	2.7E-02
242098_at	*KIAA1244*	KIAA1244	-0.463	2.7E-02
200795_at	*SPARCL1*	SPARC-like 1 (hevin)	-0.463	2.7E-02

Probeset ID	Gene symbol	Gene description	ρ	Adjusted *P*-value
206818_s_at	*CNNM2*	cyclin M2	-0.463	2.7E-02
228414_at	*KCNMA1*	potassium large conductance calcium-activated channel, subfamily M, alpha member 1	-0.463	2.7E-02
213331_s_at	*NEK1*	NIMA (never in mitosis gene a)-related kinase 1	-0.464	2.7E-02
229848_at	*ZNF10*	zinc finger protein 10	-0.464	2.7E-02
202981_x_at	*SIAH1*	siah E3 ubiquitin protein ligase 1	-0.464	2.7E-02
239726_at	*ANK3*	ankyrin 3, node of Ranvier (ankyrin G)	-0.464	2.7E-02
221483_s_at	*ARPP19*	cAMP-regulated phosphoprotein, 19kDa	-0.464	2.7E-02
230320_at	*TBRG1*	transforming growth factor beta regulator 1	-0.464	2.7E-02
202289_s_at	*TACC2*	transforming, acidic coiled-coil containing protein 2	-0.464	2.7E-02
205751_at	*SH3GL2*	SH3-domain GRB2-like 2	-0.464	2.7E-02
227996_at	*FARP1*	FERM, RhoGEF (ARHGEF) and pleckstrin domain protein 1 (chondrocyte-derived)	-0.464	2.7E-02
224561_s_at	*MORF4L1*	mortality factor 4 like 1	-0.464	2.7E-02
230178_s_at	*ELP2*	elongation protein 2 homolog (S. cerevisiae)	-0.464	2.7E-02
218297_at	*FAM188A*	family with sequence similarity 188, member A	-0.465	2.7E-02
226056_at	*ARHGAP31*	Rho GTPase activating protein 31	-0.465	2.7E-02
209442_x_at	*ANK3*	ankyrin 3, node of Ranvier (ankyrin G)	-0.465	2.7E-02
218031_s_at	*FOXN3*	forkhead box N3	-0.465	2.7E-02
229145_at	*ANAPC16*	anaphase promoting complex subunit 16	-0.465	2.7E-02
204287_at	*SYNGR1*	synaptogyrin 1	-0.465	2.7E-02
201383_s_at	*NBR1*	neighbor of BRCA1 gene 1	-0.465	2.6E-02
58308_at	*TRIM62*	tripartite motif containing 62	-0.465	2.6E-02
212901_s_at	*CSTF2T*	cleavage stimulation factor, 3' pre-RNA, subunit 2, 64kDa, tau variant	-0.465	2.6E-02
216594_x_at	*AKR1C1*	aldo-keto reductase family 1, member C1 (dihydrodiol dehydrogenase 1; 20-alpha (3-alpha)-hydroxysteroid dehydrogenase)	-0.466	2.6E-02
225420_at	*GPAM*	glycerol-3-phosphate acyltransferase, mitochondrial	-0.466	2.6E-02
1554544_a_at	*MBP*	myelin basic protein	-0.466	2.6E-02
208883_at	*UBR5*	ubiquitin protein ligase E3 component n-recognin 5	-0.466	2.6E-02
218583_s_at	*DCUN1D1*	DCN1, defective in cullin neddylation 1, domain containing 1 (S. cerevisiae)	-0.466	2.6E-02
227113_at	*ADHFE1*	alcohol dehydrogenase, iron containing, 1	-0.466	2.6E-02
233498_at	*ERBB4*	v-erb-a erythroblastic leukemia viral oncogene homolog 4 (avian)	-0.466	2.6E-02
226181_at	*TUBE1*	tubulin, epsilon 1	-0.466	2.6E-02
201366_at	*ANXA7*	annexin A7	-0.466	2.6E-02
201776_s_at	*KIAA0494*	KIAA0494	-0.466	2.6E-02
222696_at	*AXIN2*	axin 2	-0.467	2.6E-02
1553974_at	*C22orf39*	chromosome 22 open reading frame 39	-0.467	2.6E-02
209117_at	*WBP2*	WW domain binding protein 2	-0.467	2.6E-02
205732_s_at	*NCOA2*	nuclear receptor coactivator 2	-0.467	2.6E-02
213197_at	*ASTN1*	astrotactin 1	-0.467	2.6E-02
218519_at	*SLC35A5*	solute carrier family 35, member A5	-0.467	2.6E-02
215436_at	*HSDL2*	Hydroxysteroid dehydrogenase like 2	-0.467	2.6E-02
218177_at	*CHMP1B*	charged multivesicular body protein 1B	-0.467	2.6E-02
218026_at	*CCDC56*	coiled-coil domain containing 56	-0.467	2.6E-02
221795_at	*NTRK2*	neurotrophic tyrosine kinase, receptor, type 2	-0.467	2.6E-02
222148_s_at	*RHOT1*	ras homolog family member T1	-0.468	2.6E-02

Probeset ID	Gene symbol	Gene description	ρ	Adjusted *P*-value
216511_s_at	*TCF7L2*	transcription factor 7-like 2 (T-cell specific, HMG-box)	-0.468	2.5E-02
227679_at	*HDAC11*	histone deacetylase 11	-0.468	2.5E-02
38340_at	*HIP1R*	huntingtin interacting protein 1 related	-0.468	2.5E-02
234988_at	*VCPIP1*	valosin containing protein (p97)/p47 complex interacting protein 1	-0.468	2.5E-02
228174_at	*SCAI*	suppressor of cancer cell invasion	-0.468	2.5E-02
227441_s_at	*ANKS1B*	ankyrin repeat and sterile alpha motif domain containing 1B	-0.468	2.5E-02
217954_s_at	*PHF3*	PHD finger protein 3	-0.468	2.5E-02
222576_s_at	*EIF2C1*	eukaryotic translation initiation factor 2C, 1	-0.469	2.5E-02
229053_at	*SYT17*	synaptotagmin XVII	-0.469	2.5E-02
231869_at	*KIAA1586*	KIAA1586	-0.469	2.5E-02
213913_s_at	*TBC1D30*	TBC1 domain family, member 30	-0.469	2.5E-02
212758_s_at	*ZEB1*	zinc finger E-box binding homeobox 1	-0.469	2.5E-02
226913_s_at	*SOX8*	SRY (sex determining region Y)-box 8	-0.469	2.5E-02
227373_at	*ATXN1L*	ataxin 1-like	-0.469	2.5E-02
203260_at	*HDDC2*	HD domain containing 2	-0.469	2.5E-02
227516_at	*SF3A1*	splicing factor 3a, subunit 1, 120kDa	-0.469	2.5E-02
1555246_a_at	*SCN1A*	sodium channel, voltage-gated, type I, alpha subunit	-0.470	2.5E-02
200813_s_at	*PAFAH1B1*	platelet-activating factor acetylhydrolase 1b, regulatory subunit 1 (45kDa)	-0.470	2.5E-02
205139_s_at	*UST*	uronyl-2-sulfotransferase	-0.470	2.5E-02
204720_s_at	*DNAJC6*	DnaJ (Hsp40) homolog, subfamily C, member 6	-0.470	2.5E-02
226711_at	*FOXN2*	forkhead box N2	-0.470	2.5E-02
224861_at	*GNAQ*	guanine nucleotide binding protein (G protein), q polypeptide	-0.471	2.4E-02
203595_s_at	*IFIT5*	interferon-induced protein with tetratricopeptide repeats 5	-0.471	2.4E-02
212755_at	*MON2*	MON2 homolog (S. cerevisiae)	-0.471	2.4E-02
223098_s_at	*LONP2*	lon peptidase 2, peroxisomal	-0.471	2.4E-02
213186_at	*DZIP3*	DAZ interacting protein 3, zinc finger	-0.471	2.4E-02
217197_x_at	*N4BP2L1*	NEDD4 binding protein 2-like 1	-0.471	2.4E-02
225498_at	*CHMP4B*	charged multivesicular body protein 4B	-0.471	2.4E-02
235005_at	*DIS3L*	DIS3 mitotic control homolog (S. cerevisiae)-like	-0.471	2.4E-02
227908_at	*TBC1D24*	TBC1 domain family, member 24	-0.471	2.4E-02
214383_x_at	*KLHDC3*	kelch domain containing 3	-0.471	2.4E-02
242065_x_at	*IDI1*	isopentenyl-diphosphate delta isomerase 1	-0.471	2.4E-02
202250_s_at	*DCAF8*	DDB1 and CUL4 associated factor 8	-0.471	2.4E-02
215311_at	*NTRK3*	neurotrophic tyrosine kinase, receptor, type 3	-0.471	2.4E-02
226583_at	*C12orf76*	chromosome 12 open reading frame 76	-0.471	2.4E-02
227124_at	*C6orf228*	chromosome 6 open reading frame 228	-0.471	2.4E-02
228827_at	*RUNX1T1*	runt-related transcription factor 1; translocated to, 1 (cyclin D-related)	-0.472	2.4E-02
232366_at	*KIAA0232*	KIAA0232	-0.472	2.4E-02
224995_at	*SPIRE1*	spire homolog 1 (Drosophila)	-0.472	2.4E-02
202960_s_at	*MUT*	methylmalonyl CoA mutase	-0.472	2.4E-02
217868_s_at	*METTL9*	methyltransferase like 9	-0.472	2.4E-02
1553030_a_at	*SUOX*	sulfite oxidase	-0.472	2.4E-02
226049_at	*ERC1*	ELKS/RAB6-interacting/CAST family member 1	-0.472	2.4E-02
213361_at	*TDRD7*	tudor domain containing 7	-0.472	2.4E-02

Probeset ID	Gene symbol	Gene description	ρ	Adjusted *P*-value
201027_s_at	*EIF5B*	eukaryotic translation initiation factor 5B	-0.472	2.4E-02
201871_s_at	*UBXN1*	UBX domain protein 1	-0.472	2.4E-02
229213_at	*DIRC2*	disrupted in renal carcinoma 2	-0.473	2.4E-02
201674_s_at	*AKAP1*	A kinase (PRKA) anchor protein 1	-0.473	2.4E-02
203597_s_at	*WBP4*	WW domain binding protein 4 (formin binding protein 21)	-0.473	2.4E-02
203607_at	*INPP5F*	inositol polyphosphate-5-phosphatase F	-0.473	2.4E-02
212500_at	*ADO*	2-aminoethanethiol (cysteamine) dioxygenase	-0.473	2.4E-02
225483_at	*VPS26B*	vacuolar protein sorting 26 homolog B (S. pombe)	-0.473	2.4E-02
227839_at	*MBD5*	methyl-CpG binding domain protein 5	-0.473	2.4E-02
204513_s_at	*ELMO1*	engulfment and cell motility 1	-0.473	2.4E-02
225482_at	*KIF1A*	kinesin family member 1A	-0.473	2.4E-02
201738_at	*EIF1B*	eukaryotic translation initiation factor 1B	-0.473	2.3E-02
236332_at	*DHRS7*	dehydrogenase/reductase (SDR family) member 7	-0.473	2.3E-02
1555391_a_at	*ALS2CR8*	amyotrophic lateral sclerosis 2 (juvenile) chromosome region, candidate 8	-0.473	2.3E-02
213386_at	*TMEM246*	transmembrane protein 246	-0.473	2.3E-02
228177_at	*CREBBP*	CREB binding protein	-0.474	2.3E-02
230789_at	*ZNF280B*	zinc finger protein 280B	-0.474	2.3E-02
200718_s_at	*SKP1*	S-phase kinase-associated protein 1	-0.474	2.3E-02
218129_s_at	*NFYB*	nuclear transcription factor Y, beta	-0.474	2.3E-02
209258_s_at	*SMC3*	structural maintenance of chromosomes 3	-0.474	2.3E-02
228469_at	*PPID*	Peptidylprolyl isomerase D	-0.474	2.3E-02
202341_s_at	*TRIM2*	tripartite motif containing 2	-0.474	2.3E-02
210156_s_at	*PCMT1*	protein-L-isoaspartate (D-aspartate) O-methyltransferase	-0.475	2.3E-02
203552_at	*MAP4K5*	mitogen-activated protein kinase kinase kinase kinase 5	-0.475	2.3E-02
209617_s_at	*CTNND2*	catenin (cadherin-associated protein), delta 2 (neural plakophilin-related arm-repeat protein)	-0.475	2.3E-02
227539_at	*GNA13*	guanine nucleotide binding protein (G protein), alpha 13	-0.475	2.3E-02
238076_at	*GATAD2B*	GATA zinc finger domain containing 2B	-0.475	2.3E-02
216129_at	*ATP9A*	ATPase, class II, type 9A	-0.475	2.3E-02
221534_at	*C11orf68*	chromosome 11 open reading frame 68	-0.475	2.3E-02
221482_s_at	*ARPP19*	cAMP-regulated phosphoprotein, 19kDa	-0.475	2.3E-02
225810_at	*MTMR10*	myotubularin related protein 10	-0.475	2.3E-02
230479_at	*EIF3F*	eukaryotic translation initiation factor 3, subunit F	-0.475	2.3E-02
205664_at	*KIN*	KIN, antigenic determinant of recA protein homolog (mouse)	-0.476	2.3E-02
225435_at	*SSR1*	signal sequence receptor, alpha	-0.476	2.3E-02
1559517_a_at	*SPIRE1*	spire homolog 1 (Drosophila)	-0.476	2.2E-02
209221_s_at	*OSBPL2*	oxysterol binding protein-like 2	-0.476	2.2E-02
214889_at	*FAM149A*	family with sequence similarity 149, member A	-0.476	2.2E-02
221735_at	*WDR48*	WD repeat domain 48	-0.476	2.2E-02
212631_at	*STX7*	syntaxin 7	-0.476	2.2E-02
204119_s_at	*ADK*	adenosine kinase	-0.476	2.2E-02
203627_at	*IGF1R*	insulin-like growth factor 1 receptor	-0.476	2.2E-02
212502_at	*ADO*	2-aminoethanethiol (cysteamine) dioxygenase	-0.476	2.2E-02
224614_at	*DYNC1LI2*	dynein, cytoplasmic 1, light intermediate chain 2	-0.476	2.2E-02
203531_at	*CUL5*	cullin 5	-0.476	2.2E-02
203651_at	*ZFYVE16*	zinc finger, FYVE domain containing 16	-0.476	2.2E-02

Probeset ID	Gene symbol	Gene description	ρ	Adjusted *P*-value
212170_at	*RBM12*	RNA binding motif protein 12	-0.477	2.2E-02
224835_at	*GPCPD1*	glycerophosphocholine phosphodiesterase GDE1 homolog (S. cerevisiae)	-0.477	2.2E-02
227022_at	*GNPDA2*	glucosamine-6-phosphate deaminase 2	-0.477	2.2E-02
220761_s_at	*TAOK3*	TAO kinase 3	-0.477	2.2E-02
223701_s_at	*USP47*	ubiquitin specific peptidase 47	-0.477	2.2E-02
229774_at	*CXXC4*	CXXC finger protein 4	-0.477	2.2E-02
36612_at	*FAM168A*	family with sequence similarity 168, member A	-0.477	2.2E-02
210078_s_at	*KCNAB1*	potassium voltage-gated channel, shaker-related subfamily, beta member 1	-0.477	2.2E-02
226310_at	*RICTOR*	RPTOR independent companion of MTOR, complex 2	-0.477	2.2E-02
38398_at	*MADD*	MAP-kinase activating death domain	-0.477	2.2E-02
222541_at	*RSF1*	remodeling and spacing factor 1	-0.478	2.2E-02
229298_at	*KBTBD7*	kelch repeat and BTB (POZ) domain containing 7	-0.478	2.2E-02
200883_at	*UQCRC2*	ubiquinol-cytochrome c reductase core protein II	-0.478	2.2E-02
233870_at	*NAV1*	neuron navigator 1	-0.478	2.2E-02
226713_at	*CCDC50*	coiled-coil domain containing 50	-0.478	2.2E-02
219567_s_at	*DEM1*	defects in morphology 1 homolog (S. cerevisiae)	-0.478	2.2E-02
221480_at	*HNRNPD*	heterogeneous nuclear ribonucleoprotein D (AU-rich element RNA binding protein 1, 37kDa)	-0.478	2.2E-02
224764_at	*ARHGAP21*	Rho GTPase activating protein 21	-0.478	2.2E-02
222931_s_at	*THNSL1*	threonine synthase-like 1 (S. cerevisiae)	-0.478	2.2E-02
200735_x_at	*NACA*	nascent polypeptide-associated complex alpha subunit	-0.478	2.2E-02
229705_at	*PIK3C3*	phosphoinositide-3-kinase, class 3	-0.478	2.2E-02
1552789_at	*SEC62*	SEC62 homolog (S. cerevisiae)	-0.479	2.2E-02
214663_at	*DSTYK*	dual serine/threonine and tyrosine protein kinase	-0.479	2.2E-02
209430_at	*BTAF1*	BTAF1 RNA polymerase II, B-TFIID transcription factor-associated, 170kDa (Mot1 homolog, S. cerevisiae)	-0.479	2.2E-02
202922_at	*GCLC*	glutamate-cysteine ligase, catalytic subunit	-0.479	2.2E-02
219819_s_at	*MRPS28*	mitochondrial ribosomal protein S28	-0.479	2.2E-02
204635_at	*RPS6KA5*	ribosomal protein S6 kinase, 90kDa, polypeptide 5	-0.479	2.2E-02
204042_at	*WASF3*	WAS protein family, member 3	-0.479	2.2E-02
212184_s_at	*TAB2*	TGF-beta activated kinase 1/MAP3K7 binding protein 2	-0.479	2.2E-02
212735_at	*KIAA0226*	KIAA0226	-0.479	2.2E-02
230230_at	*PTPN4*	protein tyrosine phosphatase, non-receptor type 4 (megakaryocyte)	-0.479	2.2E-02
201384_s_at	*NBR1*	neighbor of BRCA1 gene 1	-0.479	2.2E-02
216885_s_at	*DCAF8*	DDB1 and CUL4 associated factor 8	-0.479	2.2E-02
218184_at	*TULP4*	tubby like protein 4	-0.479	2.2E-02
213228_at	*PDE8B*	phosphodiesterase 8B	-0.479	2.2E-02
227026_at	*MPHOSPH8*	M-phase phosphoprotein 8	-0.479	2.2E-02
229851_s_at	*C11orf54*	chromosome 11 open reading frame 54	-0.479	2.2E-02
202261_at	*VPS72*	vacuolar protein sorting 72 homolog (S. cerevisiae)	-0.480	2.2E-02
210108_at	*CACNA1D*	calcium channel, voltage-dependent, L type, alpha 1D subunit	-0.480	2.2E-02
214659_x_at	*YLPM1*	YLP motif containing 1	-0.480	2.2E-02
201927_s_at	*PKP4*	plakophilin 4	-0.480	2.1E-02
200866_s_at	*PSAP*	prosaposin	-0.480	2.1E-02
222016_s_at	*ZNF323*	zinc finger protein 323	-0.480	2.1E-02
229211_at	*DUSP28*	dual specificity phosphatase 28	-0.480	2.1E-02

Probeset ID	Gene symbol	Gene description	ρ	Adjusted *P*-value
1552790_a_at	*SEC62*	SEC62 homolog (S. cerevisiae)	-0.480	2.1E-02
203079_s_at	*CUL2*	cullin 2	-0.480	2.1E-02
214890_s_at	*FAM149A*	family with sequence similarity 149, member A	-0.480	2.1E-02
218393_s_at	*SMU1*	smu-1 suppressor of mec-8 and unc-52 homolog (C. elegans)	-0.480	2.1E-02
229940_at	*SETD3*	SET domain containing 3	-0.481	2.1E-02
210284_s_at	*TAB2*	TGF-beta activated kinase 1/MAP3K7 binding protein 2	-0.481	2.1E-02
212257_s_at	*SMARCA2*	SWI/SNF related, matrix associated, actin dependent regulator of chromatin, subfamily a, member 2	-0.481	2.1E-02
223248_at	*HSDL1*	hydroxysteroid dehydrogenase like 1	-0.481	2.1E-02
242032_at	*KIDINS220*	kinase D-interacting substrate, 220kDa	-0.481	2.1E-02
227521_at	*FBXO33*	F-box protein 33	-0.481	2.1E-02
201080_at	*PIP4K2B*	phosphatidylinositol-5-phosphate 4-kinase, type II, beta	-0.481	2.1E-02
236026_at	*GPATCH2*	G patch domain containing 2	-0.481	2.1E-02
227191_at	*ITFG1*	integrin alpha FG-GAP repeat containing 1	-0.481	2.1E-02
209750_at	*NR1D2*	nuclear receptor subfamily 1, group D, member 2	-0.481	2.1E-02
210588_x_at	*HNRNPH3*	heterogeneous nuclear ribonucleoprotein H3 (2H9)	-0.481	2.1E-02
229713_at	*PIP4K2A*	Phosphatidylinositol-5-phosphate 4-kinase, type II, alpha	-0.482	2.1E-02
229653_at	*VPS53*	vacuolar protein sorting 53 homolog (S. cerevisiae)	-0.482	2.1E-02
220841_s_at	*AHI1*	Abelson helper integration site 1	-0.482	2.1E-02
211431_s_at	*TYRO3*	TYRO3 protein tyrosine kinase	-0.482	2.1E-02
201439_at	*GBF1*	golgi brefeldin A resistant guanine nucleotide exchange factor 1	-0.482	2.1E-02
204772_s_at	*TTF1*	transcription termination factor, RNA polymerase I	-0.482	2.1E-02
212914_at	*CBX7*	chromobox homolog 7	-0.482	2.1E-02
238879_at	*DCUN1D1*	DCN1, defective in cullin neddylation 1, domain containing 1 (S. cerevisiae)	-0.482	2.1E-02
1554472_a_at	*PHF20L1*	PHD finger protein 20-like 1	-0.483	2.1E-02
1555993_at	*CACNA1D*	calcium channel, voltage-dependent, L type, alpha 1D subunit	-0.483	2.1E-02
238458_at	*EFHA2*	EF-hand domain family, member A2	-0.483	2.1E-02
212688_at	*PIK3CB*	phosphoinositide-3-kinase, catalytic, beta polypeptide	-0.483	2.1E-02
222803_at	*PRTFDC1*	phosphoribosyl transferase domain containing 1	-0.483	2.1E-02
223515_s_at	*COQ3*	coenzyme Q3 homolog, methyltransferase (S. cerevisiae)	-0.483	2.1E-02
219543_at	*PBLD*	phenazine biosynthesis-like protein domain containing	-0.483	2.1E-02
221757_at	*PIK3IP1*	phosphoinositide-3-kinase interacting protein 1	-0.483	2.1E-02
226343_at	*DPP8*	dipeptidyl-peptidase 8	-0.483	2.0E-02
212982_at	*ZDHHC17*	zinc finger, DHHC-type containing 17	-0.483	2.0E-02
225900_at	*EXOC6B*	exocyst complex component 6B	-0.483	2.0E-02
200947_s_at	*GLUD1*	glutamate dehydrogenase 1	-0.484	2.0E-02
201685_s_at	*TOX4*	TOX high mobility group box family member 4	-0.484	2.0E-02
204367_at	*SP2*	Sp2 transcription factor	-0.484	2.0E-02
207724_s_at	*SPAST*	spastin	-0.484	2.0E-02
201782_s_at	*AIP*	aryl hydrocarbon receptor interacting protein	-0.484	2.0E-02
235634_at	*PURG*	purine-rich element binding protein G	-0.484	2.0E-02
202318_s_at	*SENP6*	SUMO1/sentrin specific peptidase 6	-0.484	2.0E-02
218550_s_at	*LRRC20*	leucine rich repeat containing 20	-0.484	2.0E-02
213837_at	*L3MBTL1*	l(3)mbt-like 1 (Drosophila)	-0.484	2.0E-02
203382_s_at	*APOE*	apolipoprotein E	-0.485	2.0E-02

Probeset ID	Gene symbol	Gene description	ρ	Adjusted *P*-value
236721_at	*ALKBH1*	alkB, alkylation repair homolog 1 (E. coli)	-0.485	2.0E-02
200684_s_at	*UBE2L3*	ubiquitin-conjugating enzyme E2L 3	-0.485	2.0E-02
207127_s_at	*HNRNPH3*	heterogeneous nuclear ribonucleoprotein H3 (2H9)	-0.485	2.0E-02
202020_s_at	*LANCL1*	LanC lantibiotic synthetase component C-like 1 (bacterial)	-0.485	2.0E-02
202073_at	*OPTN*	optineurin	-0.485	2.0E-02
228456_s_at	*CDS2*	CDP-diacylglycerol synthase (phosphatidate cytidylyltransferase) 2	-0.485	2.0E-02
241469_at	*HFM1*	HFM1, ATP-dependent DNA helicase homolog (S. cerevisiae)	-0.485	2.0E-02
46270_at	*UBAP1*	ubiquitin associated protein 1	-0.485	2.0E-02
214723_x_at	*ANKRD36*	ankyrin repeat domain 36	-0.486	2.0E-02
218242_s_at	*SUV420H1*	suppressor of variegation 4-20 homolog 1 (Drosophila)	-0.486	2.0E-02
230560_at	*STXBP6*	syntaxin binding protein 6 (amisyn)	-0.486	2.0E-02
204411_at	*KIF21B*	kinesin family member 21B	-0.486	1.9E-02
229460_at	*FAM126B*	family with sequence similarity 126, member B	-0.486	1.9E-02
230329_s_at	*NUDT6*	nudix (nucleoside diphosphate linked moiety X)-type motif 6	-0.486	1.9E-02
233241_at	*PLK1S1*	polo-like kinase 1 substrate 1	-0.486	1.9E-02
218857_s_at	*ASRGL1*	asparaginase like 1	-0.486	1.9E-02
201877_s_at	*PPP2R5C*	protein phosphatase 2, regulatory subunit B', gamma	-0.487	1.9E-02
216096_s_at	*NRXN1*	neurexin 1	-0.487	1.9E-02
211034_s_at	*C12orf51*	chromosome 12 open reading frame 51	-0.487	1.9E-02
215151_at	*DOCK10*	dedicator of cytokinesis 10	-0.487	1.9E-02
218166_s_at	*RSF1*	remodeling and spacing factor 1	-0.487	1.9E-02
202361_at	*SEC24C*	SEC24 family, member C (S. cerevisiae)	-0.487	1.9E-02
221207_s_at	*NBEA*	neurobeachin	-0.487	1.9E-02
201830_s_at	*NET1*	neuroepithelial cell transforming 1	-0.487	1.9E-02
224990_at	*C4orf34*	chromosome 4 open reading frame 34	-0.487	1.9E-02
235885_at	*P2RY12*	purinergic receptor P2Y, G-protein coupled, 12	-0.487	1.9E-02
222764_at	*ASRGL1*	asparaginase like 1	-0.488	1.9E-02
228523_at	*NANOS1*	nanos homolog 1 (Drosophila)	-0.488	1.9E-02
235115_at	*PDE8B*	phosphodiesterase 8B	-0.488	1.9E-02
202033_s_at	*RB1CC1*	RB1-inducible coiled-coil 1	-0.488	1.9E-02
204137_at	*GPR137B*	G protein-coupled receptor 137B	-0.488	1.9E-02
222533_at	*CRBN*	cereblon	-0.488	1.9E-02
237408_at	*DCUN1D1*	DCN1, defective in cullin neddylation 1, domain containing 1 (S. cerevisiae)	-0.488	1.9E-02
223741_s_at	*TTYH2*	tweety homolog 2 (Drosophila)	-0.488	1.9E-02
1556695_a_at	*FLJ42709*	uncharacterized LOC441094	-0.488	1.9E-02
201800_s_at	*OSBP*	oxysterol binding protein	-0.488	1.9E-02
205895_s_at	*NOLC1*	nucleolar and coiled-body phosphoprotein 1	-0.488	1.9E-02
209234_at	*KIF1B*	kinesin family member 1B	-0.489	1.9E-02
202342_s_at	*TRIM2*	tripartite motif containing 2	-0.489	1.9E-02
226154_at	*DNM1L*	dynamin 1-like	-0.489	1.9E-02
1555905_a_at	*C3orf23*	chromosome 3 open reading frame 23	-0.489	1.9E-02
211382_s_at	*TACC2*	transforming, acidic coiled-coil containing protein 2	-0.489	1.9E-02
214724_at	*DIXDC1*	DIX domain containing 1	-0.489	1.9E-02
205353_s_at	*PEBP1*	phosphatidylethanolamine binding protein 1	-0.489	1.9E-02

Probeset ID	Gene symbol	Gene description	ρ	Adjusted *P*-value
236538_at	*GRIA2*	glutamate receptor, ionotropic, AMPA 2	-0.489	1.9E-02
213410_at	*C10orf137*	chromosome 10 open reading frame 137	-0.489	1.9E-02
219190_s_at	*EIF2C4*	eukaryotic translation initiation factor 2C, 4	-0.489	1.9E-02
205240_at	*GPSM2*	G-protein signaling modulator 2	-0.490	1.8E-02
224901_at	*SCD5*	stearoyl-CoA desaturase 5	-0.490	1.8E-02
236105_at	*ZBTB10*	zinc finger and BTB domain containing 10	-0.490	1.8E-02
214691_x_at	*FAM63B*	family with sequence similarity 63, member B	-0.490	1.8E-02
217848_s_at	*PPA1*	pyrophosphatase (inorganic) 1	-0.490	1.8E-02
228745_at	*SGTB*	small glutamine-rich tetratricopeptide repeat (TPR)-containing, beta	-0.490	1.8E-02
202590_s_at	*PDK2*	pyruvate dehydrogenase kinase, isozyme 2	-0.491	1.8E-02
226631_at	*METTL10*	methyltransferase like 10	-0.491	1.8E-02
213954_at	*FAM169A*	family with sequence similarity 169, member A	-0.491	1.8E-02
225781_at	*MAPK9*	mitogen-activated protein kinase 9	-0.491	1.8E-02
238649_at	*PITPNC1*	phosphatidylinositol transfer protein, cytoplasmic 1	-0.491	1.8E-02
209143_s_at	*CLNS1A*	chloride channel, nucleotide-sensitive, 1A	-0.491	1.8E-02
228471_at	*ANKRD44*	ankyrin repeat domain 44	-0.491	1.8E-02
203724_s_at	*RUFY3*	RUN and FYVE domain containing 3	-0.491	1.8E-02
236208_at	*MOCS2*	molybdenum cofactor synthesis 2	-0.491	1.8E-02
235579_at	*SCAF11*	SR-related CTD-associated factor 11	-0.491	1.8E-02
213351_s_at	*TMCC1*	transmembrane and coiled-coil domain family 1	-0.491	1.8E-02
203711_s_at	*HIBCH*	3-hydroxyisobutyryl-CoA hydrolase	-0.491	1.8E-02
200719_at	*SKP1*	S-phase kinase-associated protein 1	-0.492	1.8E-02
225330_at	*IGF1R*	insulin-like growth factor 1 receptor	-0.492	1.8E-02
241698_at	*RFTN2*	raftlin family member 2	-0.492	1.8E-02
217906_at	*KLHDC2*	kelch domain containing 2	-0.492	1.8E-02
224730_at	*DCAF7*	DDB1 and CUL4 associated factor 7	-0.492	1.8E-02
219641_at	*DET1*	de-etiolated homolog 1 (Arabidopsis)	-0.492	1.8E-02
225352_at	*SEC62*	SEC62 homolog (S. cerevisiae)	-0.492	1.8E-02
240172_at	*ERGIC2*	ERGIC and golgi 2	-0.492	1.8E-02
1554510_s_at	*GHITM*	growth hormone inducible transmembrane protein	-0.493	1.8E-02
214046_at	*FUT9*	fucosyltransferase 9 (alpha (1,3) fucosyltransferase)	-0.493	1.8E-02
225363_at	*PTEN*	phosphatase and tensin homolog	-0.493	1.8E-02
213758_at	*COX4I1*	cytochrome c oxidase subunit IV isoform 1	-0.493	1.8E-02
212632_at	*STX7*	syntaxin 7	-0.493	1.8E-02
200926_at	*RPS23*	ribosomal protein S23	-0.493	1.8E-02
203849_s_at	*KIF1A*	kinesin family member 1A	-0.493	1.8E-02
223118_s_at	*USP47*	ubiquitin specific peptidase 47	-0.493	1.8E-02
228051_at	*KIAA1244*	KIAA1244	-0.493	1.8E-02
211559_s_at	*CCNG2*	cyclin G2	-0.493	1.8E-02
226022_at	*SASH1*	SAM and SH3 domain containing 1	-0.493	1.8E-02
229244_at	*LSAMP*	limbic system-associated membrane protein	-0.493	1.8E-02
204753_s_at	*HLF*	hepatic leukemia factor	-0.493	1.8E-02
219834_at	*ALS2CR8*	amyotrophic lateral sclerosis 2 (juvenile) chromosome region, candidate 8	-0.493	1.8E-02
243606_at	*NXPE3*	neurexophilin and PC-esterase domain family, member 3	-0.494	1.7E-02
203031_s_at	*UROS*	uroporphyrinogen III synthase	-0.494	1.7E-02
244334_at	*TRAM1L1*	translocation associated membrane protein 1-like 1	-0.494	1.7E-02
231944_at	*ERO1LB*	ERO1-like beta (S. cerevisiae)	-0.494	1.7E-02

Probeset ID	Gene symbol	Gene description	ρ	Adjusted *P*-value
201997_s_at	*SPEN*	spen homolog, transcriptional regulator (Drosophila)	-0.495	1.7E-02
203464_s_at	*EPN2*	epsin 2	-0.495	1.7E-02
216035_x_at	*TCF7L2*	transcription factor 7-like 2 (T-cell specific, HMG-box)	-0.495	1.7E-02
228310_at	*ENAH*	enabled homolog (Drosophila)	-0.495	1.7E-02
228044_at	*SERP2*	stress-associated endoplasmic reticulum protein family member 2	-0.495	1.7E-02
239711_at	*ADAL*	adenosine deaminase-like	-0.495	1.7E-02
205152_at	*SLC6A1*	solute carrier family 6 (neurotransmitter transporter, GABA), member 1	-0.496	1.7E-02
234192_s_at	*GKAP1*	G kinase anchoring protein 1	-0.496	1.7E-02
1569108_a_at	*ZNF589*	zinc finger protein 589	-0.496	1.7E-02
238523_at	*KLHL36*	kelch-like 36 (Drosophila)	-0.496	1.7E-02
224692_at	*PPP1R15B*	protein phosphatase 1, regulatory subunit 15B	-0.496	1.7E-02
206233_at	*B4GALT6*	UDP-Gal:betaGlcNAc beta 1,4- galactosyltransferase, polypeptide 6	-0.496	1.7E-02
228108_at	*PPM1L*	protein phosphatase, Mg2+/Mn2+ dependent, 1L	-0.496	1.7E-02
226259_at	*EXOC6*	exocyst complex component 6	-0.496	1.7E-02
201971_s_at	*ATP6V1A*	ATPase, H+ transporting, lysosomal 70kDa, V1 subunit A	-0.497	1.6E-02
223960_s_at	*C16orf5*	chromosome 16 open reading frame 5	-0.497	1.6E-02
228547_at	*NRXN1*	neurexin 1	-0.497	1.6E-02
201218_at	*CTBP2*	C-terminal binding protein 2	-0.498	1.6E-02
239741_at	*PYGO1*	pygopus homolog 1 (Drosophila)	-0.498	1.6E-02
235378_at	*FAM161B*	family with sequence similarity 161, member B	-0.498	1.6E-02
224332_s_at	*MRPL43*	mitochondrial ribosomal protein L43	-0.498	1.6E-02
203376_at	*CDC40*	cell division cycle 40 homolog (S. cerevisiae)	-0.498	1.6E-02
213312_at	*C6orf162*	chromosome 6 open reading frame 162	-0.498	1.6E-02
225020_at	*DAB2IP*	DAB2 interacting protein	-0.498	1.6E-02
1558293_at	*KIAA1107*	KIAA1107	-0.498	1.6E-02
201372_s_at	*CUL3*	cullin 3	-0.498	1.6E-02
203860_at	*PCCA*	propionyl CoA carboxylase, alpha polypeptide	-0.498	1.6E-02
205052_at	*AUH*	AU RNA binding protein/enoyl-CoA hydratase	-0.498	1.6E-02
227584_at	*NAV1*	neuron navigator 1	-0.498	1.6E-02
231873_at	*BMPR2*	bone morphogenetic protein receptor, type II (serine/threonine kinase)	-0.498	1.6E-02
235787_at	*CDC37L1*	cell division cycle 37 homolog (S. cerevisiae)-like 1	-0.499	1.6E-02
1554789_a_at	*PDE8B*	phosphodiesterase 8B	-0.499	1.6E-02
206544_x_at	*SMARCA2*	SWI/SNF related, matrix associated, actin dependent regulator of chromatin, subfamily a, member 2	-0.499	1.6E-02
206806_at	*DGKI*	diacylglycerol kinase, iota	-0.499	1.6E-02
226155_at	*FAM160B1*	family with sequence similarity 160, member B1	-0.500	1.6E-02
225923_at	*VAPB*	VAMP (vesicle-associated membrane protein)-associated protein B and C	-0.500	1.6E-02
216044_x_at	*FAM69A*	family with sequence similarity 69, member A	-0.500	1.5E-02
223366_at	*ZNF704*	zinc finger protein 704	-0.500	1.5E-02
211006_s_at	*KCNB1*	potassium voltage-gated channel, Shab-related subfamily, member 1	-0.501	1.5E-02
208848_at	*ADH5*	alcohol dehydrogenase 5 (class III), chi polypeptide	-0.501	1.5E-02
227607_at	*STAMBPL1*	STAM binding protein-like 1	-0.501	1.5E-02
204072_s_at	*FRY*	furry homolog (Drosophila)	-0.501	1.5E-02

Probeset ID	Gene symbol	Gene description	ρ	Adjusted *P*-value
221326_s_at	*TUBD1*	tubulin, delta 1	-0.501	1.5E-02
205562_at	*RPP38*	ribonuclease P/MRP 38kDa subunit	-0.502	1.5E-02
221012_s_at	*TRIM8*	tripartite motif containing 8	-0.502	1.5E-02
203067_at	*PDHX*	pyruvate dehydrogenase complex, component X	-0.502	1.5E-02
223604_at	*GARNL3*	GTPase activating Rap/RanGAP domain-like 3	-0.502	1.5E-02
203232_s_at	*ATXN1*	ataxin 1	-0.502	1.5E-02
212991_at	*FBXO9*	F-box protein 9	-0.502	1.5E-02
226951_at	*C2orf49*	chromosome 2 open reading frame 49	-0.502	1.5E-02
229461_x_at	*NEGR1*	neuronal growth regulator 1	-0.502	1.5E-02
229506_at	*PPM1L*	protein phosphatase, Mg2+/Mn2+ dependent, 1L	-0.502	1.5E-02
206874_s_at	*SLK*	STE20-like kinase	-0.503	1.5E-02
226091_s_at	*MRFAP1*	Morf4 family associated protein 1	-0.503	1.5E-02
1556062_at	*RPP30*	ribonuclease P/MRP 30kDa subunit	-0.503	1.5E-02
211569_s_at	*HADH*	hydroxyacyl-CoA dehydrogenase	-0.503	1.5E-02
229687_s_at	*PRDM11*	PR domain containing 11	-0.503	1.5E-02
205878_at	*POU6F1*	POU class 6 homeobox 1	-0.503	1.5E-02
205006_s_at	*NMT2*	N-myristoyltransferase 2	-0.503	1.5E-02
209459_s_at	*ABAT*	4-aminobutyrate aminotransferase	-0.503	1.5E-02
209470_s_at	*GPM6A*	glycoprotein M6A	-0.503	1.5E-02
229355_at	*UBE2D3*	ubiquitin-conjugating enzyme E2D 3	-0.504	1.5E-02
228886_at	*LRRC27*	leucine rich repeat containing 27	-0.504	1.5E-02
205528_s_at	*RUNX1T1*	runt-related transcription factor 1; translocated to, 1 (cyclin D-related)	-0.504	1.5E-02
209236_at	*SLC23A2*	solute carrier family 23 (nucleobase transporters), member 2	-0.504	1.5E-02
203037_s_at	*MTSS1*	metastasis suppressor 1	-0.504	1.5E-02
208770_s_at	*EIF4EBP2*	eukaryotic translation initiation factor 4E binding protein 2	-0.504	1.5E-02
214553_s_at	*ARPP19*	cAMP-regulated phosphoprotein, 19kDa	-0.504	1.4E-02
201370_s_at	*CUL3*	cullin 3	-0.505	1.4E-02
209390_at	*TSC1*	tuberous sclerosis 1	-0.505	1.4E-02
217771_at	*GOLM1*	golgi membrane protein 1	-0.505	1.4E-02
225958_at	*PHC1*	polyhomeotic homolog 1 (Drosophila)	-0.505	1.4E-02
228120_at	*EIF2C1*	eukaryotic translation initiation factor 2C, 1	-0.505	1.4E-02
209700_x_at	*PDE4DIP*	phosphodiesterase 4D interacting protein	-0.505	1.4E-02
213701_at	*C12orf29*	chromosome 12 open reading frame 29	-0.505	1.4E-02
225989_at	*HERC4*	HECT and RLD domain containing E3 ubiquitin protein ligase 4	-0.506	1.4E-02
208741_at	*SAP18*	Sin3A-associated protein, 18kDa	-0.506	1.4E-02
218597_s_at	*CISD1*	CDGSH iron sulfur domain 1	-0.506	1.4E-02
204755_x_at	*HLF*	hepatic leukemia factor	-0.506	1.4E-02
209407_s_at	*DEAF1*	deformed epidermal autoregulatory factor 1 (Drosophila)	-0.506	1.4E-02
218352_at	*RCBTB1*	regulator of chromosome condensation (RCC1) and BTB (POZ) domain containing protein 1	-0.506	1.4E-02
228569_at	*PAPOLA*	poly(A) polymerase alpha	-0.506	1.4E-02
238479_at	*DCUN1D5*	DCN1, defective in cullin neddylation 1, domain containing 5 (S. cerevisiae)	-0.506	1.4E-02
40612_at	*DOPEY1*	dopey family member 1	-0.506	1.4E-02
228018_at	*NKAIN4*	Na+/K+ transporting ATPase interacting 4	-0.507	1.4E-02
236703_at	*NT5C2*	5'-nucleotidase, cytosolic II	-0.508	1.4E-02

Probeset ID	Gene symbol	Gene description	ρ	Adjusted *P*-value
212461_at	*AZIN1*	antizyme inhibitor 1	-0.508	1.4E-02
229969_at	*SEC63*	SEC63 homolog (S. cerevisiae)	-0.508	1.4E-02
235344_at	*PPM1A*	protein phosphatase, Mg2+/Mn2+ dependent, 1A	-0.508	1.4E-02
236855_at	*C10orf85*	chromosome 10 open reading frame 85	-0.508	1.4E-02
221796_at	*NTRK2*	neurotrophic tyrosine kinase, receptor, type 2	-0.508	1.3E-02
205231_s_at	*EPM2A*	epilepsy, progressive myoclonus type 2A, Lafora disease (laforin)	-0.509	1.3E-02
203244_at	*PEX5*	peroxisomal biogenesis factor 5	-0.509	1.3E-02
207974_s_at	*SKP1*	S-phase kinase-associated protein 1	-0.509	1.3E-02
221858_at	*TBC1D12*	TBC1 domain family, member 12	-0.509	1.3E-02
204421_s_at	*FGF2*	fibroblast growth factor 2 (basic)	-0.509	1.3E-02
222544_s_at	*WHSC1L1*	Wolf-Hirschhorn syndrome candidate 1-like 1	-0.509	1.3E-02
227433_at	*KIAA2018*	KIAA2018	-0.509	1.3E-02
209249_s_at	*GHITM*	growth hormone inducible transmembrane protein	-0.510	1.3E-02
213035_at	*ANKRD28*	ankyrin repeat domain 28	-0.510	1.3E-02
219770_at	*GTDC1*	glycosyltransferase-like domain containing 1	-0.510	1.3E-02
201991_s_at	*KIF5B*	kinesin family member 5B	-0.510	1.3E-02
209839_at	*DNM3*	dynamin 3	-0.510	1.3E-02
212361_s_at	*ATP2A2*	ATPase, Ca++ transporting, cardiac muscle, slow twitch 2	-0.510	1.3E-02
213314_at	*C6orf162*	chromosome 6 open reading frame 162	-0.510	1.3E-02
214736_s_at	*ADD1*	adducin 1 (alpha)	-0.510	1.3E-02
213375_s_at	*N4BP2L1*	NEDD4 binding protein 2-like 1	-0.510	1.3E-02
202813_at	*TARBP1*	TAR (HIV-1) RNA binding protein 1	-0.511	1.3E-02
213939_s_at	*RUFY3*	RUN and FYVE domain containing 3	-0.511	1.3E-02
205358_at	*GRIA2*	glutamate receptor, ionotropic, AMPA 2	-0.511	1.3E-02
209084_s_at	*RAB28*	RAB28, member RAS oncogene family	-0.511	1.3E-02
244680_at	*GLRB*	glycine receptor, beta	-0.511	1.3E-02
207757_at	*ZFP2*	zinc finger protein 2 homolog (mouse)	-0.511	1.3E-02
220488_s_at	*BCAS3*	breast carcinoma amplified sequence 3	-0.512	1.3E-02
205330_at	*MN1*	meningioma (disrupted in balanced translocation) 1	-0.512	1.3E-02
230298_at	*MBLAC2*	metallo-beta-lactamase domain containing 2	-0.512	1.3E-02
236935_at	*PTPN4*	protein tyrosine phosphatase, non-receptor type 4 (megakaryocyte)	-0.512	1.3E-02
208671_at	*SERINC1*	serine incorporator 1	-0.512	1.3E-02
1552426_a_at	*TM2D3*	TM2 domain containing 3	-0.512	1.3E-02
226018_at	*C7orf41*	chromosome 7 open reading frame 41	-0.512	1.3E-02
242224_at	*GPATCH2*	G patch domain containing 2	-0.512	1.3E-02
225009_at	*CMTM4*	CKLF-like MARVEL transmembrane domain containing 4	-0.512	1.3E-02
201781_s_at	*AIP*	aryl hydrocarbon receptor interacting protein	-0.512	1.3E-02
207103_at	*KCND2*	potassium voltage-gated channel, Shal-related subfamily, member 2	-0.513	1.3E-02
210995_s_at	*TRIM23*	tripartite motif containing 23	-0.513	1.3E-02
200787_s_at	*PEA15*	phosphoprotein enriched in astrocytes 15	-0.513	1.3E-02
227809_at	*ZC3H6*	zinc finger CCCH-type containing 6	-0.513	1.3E-02
203381_s_at	*APOE*	apolipoprotein E	-0.513	1.2E-02
1558934_a_at	*GTF2H5*	general transcription factor IIH, polypeptide 5	-0.513	1.2E-02
227930_at	*EIF2C4*	eukaryotic translation initiation factor 2C, 4	-0.513	1.2E-02

Probeset ID	Gene symbol	Gene description	ρ	Adjusted *P*-value
202386_s_at	*KIAA0430*	KIAA0430	-0.514	1.2E-02
212310_at	*MIA3*	melanoma inhibitory activity family, member 3	-0.514	1.2E-02
244623_at	*KCNQ5*	potassium voltage-gated channel, KQT-like subfamily, member 5	-0.514	1.2E-02
203006_at	*INPP5A*	inositol polyphosphate-5-phosphatase, 40kDa	-0.514	1.2E-02
229664_at	*MAPK8*	mitogen-activated protein kinase 8	-0.514	1.2E-02
238803_at	*HECTD2*	HECT domain containing E3 ubiquitin protein ligase 2	-0.515	1.2E-02
231840_x_at	*LYRM7*	Lyrm7 homolog (mouse)	-0.515	1.2E-02
226140_s_at	*OTUD1*	OTU domain containing 1	-0.515	1.2E-02
214157_at	*GNAS*	GNAS complex locus	-0.515	1.2E-02
200747_s_at	*NUMA1*	nuclear mitotic apparatus protein 1	-0.515	1.2E-02
213623_at	*KIF3A*	kinesin family member 3A	-0.516	1.2E-02
213309_at	*PLCL2*	phospholipase C-like 2	-0.516	1.2E-02
210878_s_at	*KDM3B*	lysine (K)-specific demethylase 3B	-0.516	1.2E-02
205529_s_at	*RUNX1T1*	runt-related transcription factor 1; translocated to, 1 (cyclin D-related)	-0.516	1.2E-02
212677_s_at	*CEP68*	centrosomal protein 68kDa	-0.516	1.2E-02
201706_s_at	*PEX19*	peroxisomal biogenesis factor 19	-0.516	1.2E-02
235333_at	*B4GALT6*	UDP-Gal:betaGlcNAc beta 1,4- galactosyltransferase, polypeptide 6	-0.516	1.2E-02
224820_at	*COX20*	COX20 Cox2 chaperone homolog (S. cerevisiae)	-0.517	1.2E-02
212678_at	*NF1*	neurofibromin 1	-0.517	1.2E-02
212111_at	*STX12*	syntaxin 12	-0.517	1.2E-02
229655_at	*FAM19A5*	family with sequence similarity 19 (chemokine (C-C motif)-like), member A5	-0.517	1.2E-02
204062_s_at	*ULK2*	unc-51-like kinase 2 (C. elegans)	-0.517	1.2E-02
227536_at	*ZC3H13*	zinc finger CCCH-type containing 13	-0.517	1.2E-02
229958_at	*CLN8*	ceroid-lipofuscinosis, neuronal 8 (epilepsy, progressive with mental retardation)	-0.517	1.2E-02
209112_at	*CDKN1B*	cyclin-dependent kinase inhibitor 1B (p27, Kip1)	-0.517	1.2E-02
212943_at	*KIAA0528*	KIAA0528	-0.517	1.2E-02
224215_s_at	*DLL1*	delta-like 1 (Drosophila)	-0.517	1.2E-02
222111_at	*FAM63B*	family with sequence similarity 63, member B	-0.518	1.1E-02
208953_at	*LARP4B*	La ribonucleoprotein domain family, member 4B	-0.518	1.1E-02
212990_at	*SYNJ1*	synaptojanin 1	-0.518	1.1E-02
229459_at	*FAM19A5*	family with sequence similarity 19 (chemokine (C-C motif)-like), member A5	-0.518	1.1E-02
230029_x_at	*UBR3*	ubiquitin protein ligase E3 component n-recognin 3 (putative)	-0.518	1.1E-02
1554806_a_at	*FBXO8*	F-box protein 8	-0.519	1.1E-02
211376_s_at	*NSMCE4A*	non-SMC element 4 homolog A (S. cerevisiae)	-0.519	1.1E-02
213352_at	*TMCC1*	transmembrane and coiled-coil domain family 1	-0.519	1.1E-02
223536_at	*PSD2*	pleckstrin and Sec7 domain containing 2	-0.519	1.1E-02
212757_s_at	*CAMK2G*	calcium/calmodulin-dependent protein kinase II gamma	-0.519	1.1E-02
214680_at	*NTRK2*	neurotrophic tyrosine kinase, receptor, type 2	-0.520	1.1E-02
235494_at	*LSAMP*	limbic system-associated membrane protein	-0.520	1.1E-02
202777_at	*SHOC2*	soc-2 suppressor of clear homolog (C. elegans)	-0.520	1.1E-02
230826_at	*MMD2*	monocyte to macrophage differentiation-associated 2	-0.520	1.1E-02
227094_at	*DHTKD1*	dehydrogenase E1 and transketolase domain containing 1	-0.520	1.1E-02
235048_at	*FAM169A*	family with sequence similarity 169, member A	-0.520	1.1E-02

Probeset ID	Gene symbol	Gene description	ρ	Adjusted *P*-value
200940_s_at	*RERE*	arginine-glutamic acid dipeptide (RE) repeats	-0.520	1.1E-02
214735_at	*IPCEF1*	interaction protein for cytohesin exchange factors 1	-0.520	1.1E-02
228446_at	*KIAA2026*	KIAA2026	-0.520	1.1E-02
223281_s_at	*COX15*	COX15 homolog, cytochrome c oxidase assembly protein (yeast)	-0.521	1.1E-02
238955_at	*RFTN2*	raftlin family member 2	-0.521	1.1E-02
211937_at	*EIF4B*	eukaryotic translation initiation factor 4B	-0.521	1.1E-02
228680_at	*KIF3A*	kinesin family member 3A	-0.521	1.1E-02
212850_s_at	*LRP4*	low density lipoprotein receptor-related protein 4	-0.522	1.1E-02
226520_at	*LCOR*	ligand dependent nuclear receptor corepressor	-0.522	1.1E-02
225497_at	*ATE1*	arginyltransferase 1	-0.522	1.1E-02
228916_at	*CWF19L2*	CWF19-like 2, cell cycle control (S. pombe)	-0.522	1.1E-02
1561394_s_at	*KIAA1755*	KIAA1755	-0.523	1.1E-02
224366_s_at	*REPS1*	RALBP1 associated Eps domain containing 1	-0.523	1.1E-02
224150_s_at	*CEP70*	centrosomal protein 70kDa	-0.523	1.1E-02
216195_at	*ANK2*	ankyrin 2, neuronal	-0.523	1.0E-02
227896_at	*BCCIP*	BRCA2 and CDKN1A interacting protein	-0.523	1.0E-02
202259_s_at	*N4BP2L2*	NEDD4 binding protein 2-like 2	-0.523	1.0E-02
206183_s_at	*HERC3*	HECT and RLD domain containing E3 ubiquitin protein ligase 3	-0.523	1.0E-02
224946_s_at	*CCDC115*	coiled-coil domain containing 115	-0.524	1.0E-02
227978_s_at	*ZADH2*	zinc binding alcohol dehydrogenase domain containing 2	-0.524	1.0E-02
1565681_s_at	*DIP2C*	DIP2 disco-interacting protein 2 homolog C (Drosophila)	-0.524	1.0E-02
213133_s_at	*GCSH*	glycine cleavage system protein H (aminomethyl carrier)	-0.524	1.0E-02
207232_s_at	*DZIP3*	DAZ interacting protein 3, zinc finger	-0.524	1.0E-02
227112_at	*TMCC1*	transmembrane and coiled-coil domain family 1	-0.525	1.0E-02
211662_s_at	*VDAC2*	voltage-dependent anion channel 2	-0.525	1.0E-02
223059_s_at	*FAM107B*	family with sequence similarity 107, member B	-0.525	1.0E-02
244008_at	*PARP8*	poly (ADP-ribose) polymerase family, member 8	-0.525	1.0E-02
225066_at	*PPP2R2D*	protein phosphatase 2, regulatory subunit B, delta	-0.525	1.0E-02
226623_at	*PHYHIPL*	phytanoyl-CoA 2-hydroxylase interacting protein-like	-0.525	9.9E-03
232341_x_at	*HABP4*	hyaluronan binding protein 4	-0.525	9.9E-03
231856_at	*KIAA1244*	KIAA1244	-0.526	9.9E-03
61297_at	*CASKIN2*	CASK interacting protein 2	-0.526	9.9E-03
212409_s_at	*TOR1AIP1*	torsin A interacting protein 1	-0.526	9.9E-03
226151_x_at	*CRYZL1*	crystallin, zeta (quinone reductase)-like 1	-0.526	9.9E-03
208943_s_at	*SEC62*	SEC62 homolog (S. cerevisiae)	-0.526	9.9E-03
222983_s_at	*PAIP2*	poly(A) binding protein interacting protein 2	-0.526	9.9E-03
203146_s_at	*GABBR1*	gamma-aminobutyric acid (GABA) B receptor, 1	-0.526	9.9E-03
221699_s_at	*DDX50*	DEAD (Asp-Glu-Ala-Asp) box polypeptide 50	-0.526	9.9E-03
203420_at	*FAM8A1*	family with sequence similarity 8, member A1	-0.526	9.9E-03
210554_s_at	*CTBP2*	C-terminal binding protein 2	-0.526	9.9E-03
228170_at	*OLIG1*	oligodendrocyte transcription factor 1	-0.526	9.9E-03
223046_at	*EGLN1*	egl nine homolog 1 (C. elegans)	-0.527	9.8E-03
213392_at	*IQCK*	IQ motif containing K	-0.527	9.8E-03
203526_s_at	*APC*	adenomatous polyposis coli	-0.527	9.7E-03
229648_at	*ARHGAP32*	Rho GTPase activating protein 32	-0.527	9.7E-03
209915_s_at	*NRXN1*	neurexin 1	-0.527	9.6E-03
227257_s_at	*CACUL1*	CDK2-associated, cullin domain 1	-0.527	9.6E-03

Probeset ID	Gene symbol	Gene description	ρ	Adjusted *P*-value
1557950_at	*NEMF*	nuclear export mediator factor	-0.527	9.6E-03
218142_s_at	*CRBN*	cereblon	-0.528	9.6E-03
63305_at	*PKNOX2*	PBX/knotted 1 homeobox 2	-0.528	9.6E-03
212765_at	*CAMSAP2*	calmodulin regulated spectrin-associated protein family, member 2	-0.528	9.5E-03
223412_at	*KBTBD7*	kelch repeat and BTB (POZ) domain containing 7	-0.528	9.5E-03
228380_at	*SENP2*	SUMO1/sentrin/SMT3 specific peptidase 2	-0.528	9.5E-03
221498_at	*SNX27*	sorting nexin family member 27	-0.528	9.5E-03
202630_at	*APPBP2*	amyloid beta precursor protein (cytoplasmic tail) binding protein 2	-0.528	9.5E-03
209702_at	*FTO*	fat mass and obesity associated	-0.528	9.5E-03
204156_at	*SIK3*	SIK family kinase 3	-0.528	9.5E-03
227132_at	*ZNF706*	zinc finger protein 706	-0.528	9.5E-03
228841_at	*LYRM7*	Lyrm7 homolog (mouse)	-0.528	9.5E-03
1557370_s_at	*MYCBP2*	MYC binding protein 2, E3 ubiquitin protein ligase	-0.529	9.3E-03
204021_s_at	*PURA*	purine-rich element binding protein A	-0.529	9.3E-03
202319_at	*SENP6*	SUMO1/sentrin specific peptidase 6	-0.529	9.3E-03
227251_at	*DCAF5*	DDB1 and CUL4 associated factor 5	-0.529	9.3E-03
201829_at	*NET1*	neuroepithelial cell transforming 1	-0.529	9.3E-03
213362_at	*PTPRD*	protein tyrosine phosphatase, receptor type, D	-0.530	9.3E-03
214790_at	*SENP6*	SUMO1/sentrin specific peptidase 6	-0.530	9.3E-03
213922_at	*TTBK2*	tau tubulin kinase 2	-0.530	9.2E-03
1554627_a_at	*ASCC1*	activating signal cointegrator 1 complex subunit 1	-0.530	9.2E-03
233919_s_at	*HABP4*	hyaluronan binding protein 4	-0.530	9.2E-03
235309_at	*RPS15A*	ribosomal protein S15a	-0.530	9.2E-03
200946_x_at	*GLUD1*	glutamate dehydrogenase 1	-0.530	9.2E-03
38892_at	*KIAA0240*	KIAA0240	-0.530	9.1E-03
203249_at	*EZH1*	enhancer of zeste homolog 1 (Drosophila)	-0.531	9.1E-03
203839_s_at	*TNK2*	tyrosine kinase, non-receptor, 2	-0.531	9.0E-03
221430_s_at	*RNF146*	ring finger protein 146	-0.531	9.0E-03
212530_at	*NEK7*	NIMA (never in mitosis gene a)-related kinase 7	-0.531	9.0E-03
1553765_a_at	*KLHL32*	kelch-like 32 (Drosophila)	-0.531	8.9E-03
227416_s_at	*ZCRB1*	zinc finger CCHC-type and RNA binding motif 1	-0.532	8.9E-03
222737_s_at	*BRD7*	bromodomain containing 7	-0.532	8.9E-03
223119_s_at	*USP47*	ubiquitin specific peptidase 47	-0.532	8.9E-03
225658_at	*SPOPL*	speckle-type POZ protein-like	-0.532	8.9E-03
1555141_a_at	*SLC9B1*	solute carrier family 9, subfamily B (NHA1, cation proton antiporter 1), member 1	-0.532	8.8E-03
232136_s_at	*CTTNBP2*	cortactin binding protein 2	-0.532	8.8E-03
215785_s_at	*CYFIP2*	cytoplasmic FMR1 interacting protein 2	-0.532	8.8E-03
241739_at	*OGFOD1*	2-oxoglutarate and iron-dependent oxygenase domain containing 1	-0.533	8.7E-03
209066_x_at	*UQCRB*	ubiquinol-cytochrome c reductase binding protein	-0.533	8.7E-03
211600_at	*PTPRO*	protein tyrosine phosphatase, receptor type, O	-0.533	8.7E-03
214318_s_at	*FRY*	furry homolog (Drosophila)	-0.533	8.7E-03
209257_s_at	*SMC3*	structural maintenance of chromosomes 3	-0.533	8.7E-03
204633_s_at	*RPS6KA5*	ribosomal protein S6 kinase, 90kDa, polypeptide 5	-0.533	8.7E-03
222516_at	*AP3M1*	adaptor-related protein complex 3, mu 1 subunit	-0.533	8.7E-03
225429_at	*PPP6C*	protein phosphatase 6, catalytic subunit	-0.534	8.6E-03

Probeset ID	Gene symbol	Gene description	ρ	Adjusted *P*-value
212455_at	*YTHDC1*	YTH domain containing 1	-0.534	8.6E-03
212609_s_at	*AKT3*	v-akt murine thymoma viral oncogene homolog 3 (protein kinase B, gamma)	-0.534	8.6E-03
224998_at	*CMTM4*	CKLF-like MARVEL transmembrane domain containing 4	-0.534	8.6E-03
226748_at	*LYSMD2*	LysM, putative peptidoglycan-binding, domain containing 2	-0.534	8.6E-03
214787_at	*DENND4A*	DENN/MADD domain containing 4A	-0.534	8.6E-03
228062_at	*NAP1L5*	nucleosome assembly protein 1-like 5	-0.534	8.5E-03
244293_at	*UQCRB*	ubiquinol-cytochrome c reductase binding protein	-0.535	8.5E-03
238490_at	*KIAA2026*	KIAA2026	-0.535	8.4E-03
225915_at	*CAB39L*	calcium binding protein 39-like	-0.535	8.3E-03
228561_at	*CDC37L1*	cell division cycle 37 homolog (S. cerevisiae)-like 1	-0.535	8.3E-03
209582_s_at	*CD200*	CD200 molecule	-0.535	8.3E-03
212880_at	*WDR7*	WD repeat domain 7	-0.535	8.3E-03
242569_at	*STAM2*	signal transducing adaptor molecule (SH3 domain and ITAM motif) 2	-0.535	8.3E-03
219279_at	*DOCK10*	dedicator of cytokinesis 10	-0.536	8.2E-03
207091_at	*P2RX7*	purinergic receptor P2X, ligand-gated ion channel, 7	-0.536	8.1E-03
212306_at	*CLASP2*	cytoplasmic linker associated protein 2	-0.537	8.1E-03
218970_s_at	*CUTC*	cutC copper transporter homolog (E. coli)	-0.537	8.1E-03
223438_s_at	*PPARA*	peroxisome proliferator-activated receptor alpha	-0.537	8.1E-03
204762_s_at	*GNAO1*	guanine nucleotide binding protein (G protein), alpha activating activity polypeptide O	-0.537	8.1E-03
227599_at	*MB21D2*	Mab-21 domain containing 2	-0.537	8.1E-03
228376_at	*GGTA1P*	glycoprotein, alpha-galactosyltransferase 1 pseudogene	-0.537	8.1E-03
235024_at	*PHF17*	PHD finger protein 17	-0.537	8.1E-03
235264_at	*HCFC2*	host cell factor C2	-0.537	8.1E-03
228063_s_at	*NAP1L5*	nucleosome assembly protein 1-like 5	-0.537	8.0E-03
202079_s_at	*TRAK1*	trafficking protein, kinesin binding 1	-0.537	8.0E-03
230056_at	*BPTF*	bromodomain PHD finger transcription factor	-0.537	8.0E-03
230942_at	*CMTM5*	CKLF-like MARVEL transmembrane domain containing 5	-0.538	8.0E-03
231358_at	*MRO*	maestro	-0.538	8.0E-03
211203_s_at	*CNTN1*	contactin 1	-0.538	8.0E-03
202742_s_at	*PRKACB*	protein kinase, cAMP-dependent, catalytic, beta	-0.538	8.0E-03
204284_at	*PPP1R3C*	protein phosphatase 1, regulatory subunit 3C	-0.538	8.0E-03
210471_s_at	*KCNAB1*	potassium voltage-gated channel, shaker-related subfamily, beta member 1	-0.538	8.0E-03
225334_at	*C10orf32*	chromosome 10 open reading frame 32	-0.538	8.0E-03
226500_at	*ZBTB47*	zinc finger and BTB domain containing 47	-0.538	8.0E-03
244783_at	*YAF2*	YY1 associated factor 2	-0.539	7.9E-03
1569107_s_at	*ZNF642*	zinc finger protein 642	-0.539	7.9E-03
222517_at	*AP3M1*	adaptor-related protein complex 3, mu 1 subunit	-0.539	7.9E-03
207797_s_at	*LRP2BP*	LRP2 binding protein	-0.539	7.8E-03
225048_at	*PHF10*	PHD finger protein 10	-0.539	7.8E-03
211383_s_at	*WDR37*	WD repeat domain 37	-0.539	7.8E-03
225884_s_at	*GZF1*	GDNF-inducible zinc finger protein 1	-0.539	7.8E-03
202011_at	*TJP1*	tight junction protein 1 (zona occludens 1)	-0.540	7.7E-03
212928_at	*TSPYL4*	TSPY-like 4	-0.540	7.7E-03

Probeset ID	Gene symbol	Gene description	ρ	Adjusted *P*-value
225886_at	*DDX5*	DEAD (Asp-Glu-Ala-Asp) box helicase 5	-0.540	7.7E-03
209300_s_at	*NECAP1*	NECAP endocytosis associated 1	-0.540	7.7E-03
210110_x_at	*HNRNPH3*	heterogeneous nuclear ribonucleoprotein H3 (2H9)	-0.540	7.7E-03
225086_at	*FAM98B*	family with sequence similarity 98, member B	-0.540	7.7E-03
1552727_s_at	*ADAMTS17*	ADAM metallopeptidase with thrombospondin type 1 motif, 17	-0.540	7.7E-03
203188_at	*B3GNT1*	UDP-GlcNAc:betaGal beta-1,3-N-acetylglucosaminyltransferase 1	-0.540	7.7E-03
230821_at	*ZNF148*	zinc finger protein 148	-0.540	7.7E-03
1563475_s_at	*METTL20*	methyltransferase like 20	-0.541	7.7E-03
221082_s_at	*NDRG3*	NDRG family member 3	-0.541	7.6E-03
205420_at	*PEX7*	peroxisomal biogenesis factor 7	-0.541	7.6E-03
226855_at	*PDP2*	pyruvate dehyrogenase phosphatase catalytic subunit 2	-0.541	7.6E-03
235405_at	*GSTA4*	glutathione S-transferase alpha 4	-0.541	7.6E-03
201335_s_at	*ARHGEF12*	Rho guanine nucleotide exchange factor (GEF) 12	-0.541	7.6E-03
215992_s_at	*RAPGEF2*	Rap guanine nucleotide exchange factor (GEF) 2	-0.542	7.5E-03
218723_s_at	*RGCC*	regulator of cell cycle	-0.542	7.5E-03
226234_at	*GDF11*	growth differentiation factor 11	-0.542	7.5E-03
208611_s_at	*SPTAN1*	spectrin, alpha, non-erythrocytic 1	-0.542	7.4E-03
208732_at	*RAB2A*	RAB2A, member RAS oncogene family	-0.542	7.4E-03
238794_at	*SFR1*	SWI5-dependent recombination repair 1	-0.542	7.4E-03
226751_at	*CNRIP1*	cannabinoid receptor interacting protein 1	-0.543	7.4E-03
219800_s_at	*THNSL1*	threonine synthase-like 1 (S. cerevisiae)	-0.543	7.3E-03
212079_s_at	*MLL*	myeloid/lymphoid or mixed-lineage leukemia (trithorax homolog, Drosophila)	-0.543	7.3E-03
213266_at	*TUBGCP4*	Tubulin, gamma complex associated protein 4	-0.543	7.3E-03
235111_at	*LSAMP*	limbic system-associated membrane protein	-0.543	7.3E-03
221922_at	*GPSM2*	G-protein signaling modulator 2	-0.543	7.3E-03
212760_at	*UBR2*	ubiquitin protein ligase E3 component n-recognin 2	-0.544	7.2E-03
228581_at	*KCNJ10*	potassium inwardly-rectifying channel, subfamily J, member 10	-0.544	7.2E-03
201174_s_at	*TERF2IP*	telomeric repeat binding factor 2, interacting protein	-0.544	7.2E-03
220391_at	*ZBTB3*	zinc finger and BTB domain containing 3	-0.544	7.2E-03
212693_at	*MDN1*	MDN1, midasin homolog (yeast)	-0.544	7.2E-03
1555469_a_at	*CLASP2*	cytoplasmic linker associated protein 2	-0.544	7.2E-03
219049_at	*CSGALNACT1*	chondroitin sulfate N-acetylgalactosaminyltransferase 1	-0.544	7.1E-03
212425_at	*SCAMP1*	secretory carrier membrane protein 1	-0.544	7.1E-03
240806_at	*RPL15*	Ribosomal protein L15	-0.544	7.1E-03
228218_at	*LSAMP*	limbic system-associated membrane protein	-0.545	7.1E-03
228228_at	*DACT3*	dapper, antagonist of beta-catenin, homolog 3 (Xenopus laevis)	-0.545	7.1E-03
212793_at	*DAAM2*	dishevelled associated activator of morphogenesis 2	-0.545	7.1E-03
1569349_at	*C11orf30*	chromosome 11 open reading frame 30	-0.545	7.1E-03
235635_at	*ARHGAP5*	Rho GTPase activating protein 5	-0.545	7.1E-03
208666_s_at	*ST13*	suppression of tumorigenicity 13 (colon carcinoma) (Hsp70 interacting protein)	-0.545	7.0E-03
211475_s_at	*BAG1*	BCL2-associated athanogene	-0.545	7.0E-03
242255_at	*WDR37*	WD repeat domain 37	-0.545	7.0E-03
228906_at	*TET1*	tet methylcytosine dioxygenase 1	-0.546	7.0E-03
229676_at	*MTPAP*	Mitochondrial poly(A) polymerase	-0.546	7.0E-03

Probeset ID	Gene symbol	Gene description	ρ	Adjusted *P*-value
229414_at	*PITPNC1*	phosphatidylinositol transfer protein, cytoplasmic 1	-0.546	7.0E-03
210625_s_at	*AKAP1*	A kinase (PRKA) anchor protein 1	-0.546	7.0E-03
212731_at	*ANKRD46*	ankyrin repeat domain 46	-0.546	7.0E-03
226779_at	*LMBRD2*	LMBR1 domain containing 2	-0.546	7.0E-03
204476_s_at	*PC*	pyruvate carboxylase	-0.546	7.0E-03
212719_at	*PHLPP1*	PH domain and leucine rich repeat protein phosphatase 1	-0.546	7.0E-03
239024_at	*ZNF148*	zinc finger protein 148	-0.546	7.0E-03
214251_s_at	*NUMA1*	nuclear mitotic apparatus protein 1	-0.547	6.9E-03
225729_at	*C6orf89*	chromosome 6 open reading frame 89	-0.547	6.9E-03
209002_s_at	*CALCOCO1*	calcium binding and coiled-coil domain 1	-0.547	6.9E-03
219547_at	*COX15*	COX15 homolog, cytochrome c oxidase assembly protein (yeast)	-0.547	6.9E-03
202650_s_at	*KIAA0195*	KIAA0195	-0.547	6.9E-03
211951_at	*NOLC1*	nucleolar and coiled-body phosphoprotein 1	-0.548	6.8E-03
223507_at	*CLPX*	ClpX caseinolytic peptidase X homolog (E. coli)	-0.548	6.8E-03
201711_x_at	*RANBP2*	RAN binding protein 2	-0.548	6.8E-03
1554767_s_at	*CRYZL1*	crystallin, zeta (quinone reductase)-like 1	-0.548	6.8E-03
209111_at	*RNF5*	ring finger protein 5, E3 ubiquitin protein ligase	-0.548	6.7E-03
209916_at	*DHTKD1*	dehydrogenase E1 and transketolase domain containing 1	-0.548	6.7E-03
207093_s_at	*OMG*	oligodendrocyte myelin glycoprotein	-0.548	6.7E-03
226106_at	*RNF141*	ring finger protein 141	-0.549	6.7E-03
204155_s_at	*SIK3*	SIK family kinase 3	-0.549	6.7E-03
204020_at	*PURA*	purine-rich element binding protein A	-0.549	6.7E-03
227605_at	*AIMP1*	aminoacyl tRNA synthetase complex-interacting multifunctional protein 1	-0.549	6.6E-03
222941_at	*USP46*	ubiquitin specific peptidase 46	-0.549	6.6E-03
205104_at	*SNPH*	syntaphilin	-0.550	6.6E-03
201599_at	*OAT*	ornithine aminotransferase	-0.550	6.6E-03
212598_at	*WDFY3*	WD repeat and FYVE domain containing 3	-0.550	6.6E-03
201718_s_at	*EPB41L2*	erythrocyte membrane protein band 4.1-like 2	-0.550	6.5E-03
218097_s_at	*CUEDC2*	CUE domain containing 2	-0.550	6.5E-03
238818_at	*KIAA1429*	KIAA1429	-0.550	6.5E-03
212655_at	*ZCCHC14*	zinc finger, CCHC domain containing 14	-0.550	6.5E-03
223058_at	*FAM107B*	family with sequence similarity 107, member B	-0.550	6.5E-03
229497_at	*ANKDD1A*	ankyrin repeat and death domain containing 1A	-0.550	6.5E-03
204100_at	*THRA*	thyroid hormone receptor, alpha	-0.551	6.5E-03
218528_s_at	*RNF38*	ring finger protein 38	-0.551	6.5E-03
1556061_at	*RPP30*	ribonuclease P/MRP 30kDa subunit	-0.551	6.5E-03
221508_at	*TAOK3*	TAO kinase 3	-0.551	6.5E-03
212779_at	*KIAA1109*	KIAA1109	-0.551	6.4E-03
226443_at	*FAM122A*	family with sequence similarity 122A	-0.551	6.4E-03
225550_at	*CNST*	consortin, connexin sorting protein	-0.551	6.4E-03
218761_at	*RNF111*	ring finger protein 111	-0.552	6.4E-03
218935_at	*EHD3*	EH-domain containing 3	-0.552	6.4E-03
224622_at	*TBC1D14*	TBC1 domain family, member 14	-0.552	6.4E-03
238569_at	*GABBR1*	gamma-aminobutyric acid (GABA) B receptor, 1	-0.552	6.4E-03
209914_s_at	*NRXN1*	neurexin 1	-0.552	6.3E-03
213218_at	*ZNF187*	zinc finger protein 187	-0.552	6.3E-03
208868_s_at	*GABARAPL1*	GABA(A) receptor-associated protein like 1	-0.552	6.3E-03

Probeset ID	Gene symbol	Gene description	ρ	Adjusted *P*-value
225840_at	*TEF*	thyrotrophic embryonic factor	-0.552	6.3E-03
219643_at	*LRP1B*	low density lipoprotein receptor-related protein 1B	-0.553	6.3E-03
227977_at	*ZADH2*	zinc binding alcohol dehydrogenase domain containing 2	-0.553	6.3E-03
209019_s_at	*PINK1*	PTEN induced putative kinase 1	-0.553	6.3E-03
212427_at	*KIAA0368*	KIAA0368	-0.553	6.3E-03
202457_s_at	*PPP3CA*	protein phosphatase 3, catalytic subunit, alpha isozyme	-0.553	6.3E-03
226104_at	*RNF170*	ring finger protein 170	-0.553	6.3E-03
228017_s_at	*NKAIN4*	Na+/K+ transporting ATPase interacting 4	-0.554	6.2E-03
224781_s_at	*RBM17*	RNA binding motif protein 17	-0.554	6.2E-03
225064_at	*RABEP1*	rabaptin, RAB GTPase binding effector protein 1	-0.554	6.2E-03
203883_s_at	*RAB11FIP2*	RAB11 family interacting protein 2 (class I)	-0.554	6.2E-03
208740_at	*SAP18*	Sin3A-associated protein, 18kDa	-0.554	6.1E-03
201320_at	*SMARCC2*	SWI/SNF related, matrix associated, actin dependent regulator of chromatin, subfamily c, member 2	-0.554	6.1E-03
217896_s_at	*FAM192A*	family with sequence similarity 192, member A	-0.554	6.1E-03
225446_at	*BRWD1*	bromodomain and WD repeat domain containing 1	-0.554	6.1E-03
215945_s_at	*TRIM2*	tripartite motif containing 2	-0.554	6.1E-03
220345_at	*LRRTM4*	leucine rich repeat transmembrane neuronal 4	-0.554	6.1E-03
237094_at	*FAM19A5*	family with sequence similarity 19 (chemokine (C-C motif)-like), member A5	-0.554	6.1E-03
228006_at	*PTEN*	phosphatase and tensin homolog	-0.555	6.1E-03
209864_at	*FRAT2*	frequently rearranged in advanced T-cell lymphomas 2	-0.555	6.1E-03
226345_at	*ARL5B*	ADP-ribosylation factor-like 5B	-0.555	6.1E-03
209259_s_at	*SMC3*	structural maintenance of chromosomes 3	-0.555	6.1E-03
235227_at	*STXBP5*	syntaxin binding protein 5 (tomosyn)	-0.555	6.1E-03
1560814_a_at	*C15orf57*	chromosome 15 open reading frame 57	-0.555	6.0E-03
212309_at	*CLASP2*	cytoplasmic linker associated protein 2	-0.556	6.0E-03
1564002_a_at	*AKD1*	adenylate kinase domain containing 1	-0.556	6.0E-03
202967_at	*GSTA4*	glutathione S-transferase alpha 4	-0.556	6.0E-03
200898_s_at	*MGEA5*	meningioma expressed antigen 5 (hyaluronidase)	-0.556	6.0E-03
227401_at	*IL17D*	interleukin 17D	-0.556	6.0E-03
230129_at	*PSTK*	phosphoseryl-tRNA kinase	-0.556	6.0E-03
215235_at	*SPTAN1*	spectrin, alpha, non-erythrocytic 1	-0.556	5.9E-03
206689_x_at	*KAT5*	K(lysine) acetyltransferase 5	-0.557	5.9E-03
243843_at	*N4BP2L1*	NEDD4 binding protein 2-like 1	-0.557	5.9E-03
203584_at	*EMC2*	ER membrane protein complex subunit 2	-0.557	5.9E-03
212124_at	*ZMIZ1*	zinc finger, MIZ-type containing 1	-0.557	5.8E-03
224652_at	*CCNY*	cyclin Y	-0.558	5.8E-03
226280_at	*BNIP2*	BCL2/adenovirus E1B 19kDa interacting protein 2	-0.558	5.8E-03
221550_at	*COX15*	COX15 homolog, cytochrome c oxidase assembly protein (yeast)	-0.558	5.8E-03
201807_at	*VPS26A*	vacuolar protein sorting 26 homolog A (S. pombe)	-0.558	5.8E-03
218976_at	*DNAJC12*	DnaJ (Hsp40) homolog, subfamily C, member 12	-0.558	5.7E-03
203631_s_at	*GPRC5B*	G protein-coupled receptor, family C, group 5, member B	-0.558	5.7E-03
209982_s_at	*NRXN2*	neurexin 2	-0.558	5.7E-03
209460_at	*ABAT*	4-aminobutyrate aminotransferase	-0.558	5.7E-03
221588_x_at	*ALDH6A1*	aldehyde dehydrogenase 6 family, member A1	-0.559	5.7E-03
205259_at	*NR3C2*	nuclear receptor subfamily 3, group C, member 2	-0.559	5.6E-03
218228_s_at	*TNKS2*	tankyrase, TRF1-interacting ankyrin-related ADP-ribose	-0.559	5.6E-03

Probeset ID	Gene symbol	Gene description	ρ	Adjusted P-value
		polymerase 2		
232226_at	*LRRC4C*	leucine rich repeat containing 4C	-0.559	5.6E-03
205279_s_at	*GLRB*	glycine receptor, beta	-0.559	5.6E-03
209311_at	*BCL2L2*	BCL2-like 2	-0.559	5.6E-03
224662_at	*KIF5B*	kinesin family member 5B	-0.559	5.6E-03
226648_at	*HIF1AN*	hypoxia inducible factor 1, alpha subunit inhibitor	-0.559	5.6E-03
226366_at	*SHPRH*	SNF2 histone linker PHD RING helicase, E3 ubiquitin protein ligase	-0.559	5.6E-03
238472_at	*FBXO9*	F-box protein 9	-0.559	5.6E-03
228695_at	*C8orf46*	chromosome 8 open reading frame 46	-0.560	5.6E-03
234977_at	*ZADH2*	zinc binding alcohol dehydrogenase domain containing 2	-0.560	5.6E-03
235345_at	*RUFY2*	RUN and FYVE domain containing 2	-0.560	5.6E-03
203853_s_at	*GAB2*	GRB2-associated binding protein 2	-0.560	5.5E-03
204067_at	*SUOX*	sulfite oxidase	-0.560	5.5E-03
229312_s_at	*GKAP1*	G kinase anchoring protein 1	-0.561	5.5E-03
200788_s_at	*PEA15*	phosphoprotein enriched in astrocytes 15	-0.561	5.5E-03
201988_s_at	*CREBL2*	cAMP responsive element binding protein-like 2	-0.561	5.5E-03
225405_at	*HNRNPUL2*	heterogeneous nuclear ribonucleoprotein U-like 2	-0.561	5.5E-03
226712_at	*SSR1*	signal sequence receptor, alpha	-0.562	5.4E-03
220753_s_at	*CRYL1*	crystallin, lambda 1	-0.562	5.4E-03
225426_at	*PPP6C*	protein phosphatase 6, catalytic subunit	-0.562	5.4E-03
239300_at	*PIK3C3*	phosphoinositide-3-kinase, class 3	-0.562	5.4E-03
213118_at	*UHRF1BP1L*	UHRF1 binding protein 1-like	-0.562	5.3E-03
203884_s_at	*RAB11FIP2*	RAB11 family interacting protein 2 (class I)	-0.563	5.3E-03
202794_at	*INPP1*	inositol polyphosphate-1-phosphatase	-0.563	5.2E-03
208939_at	*SEPHS1*	selenophosphate synthetase 1	-0.563	5.2E-03
209860_s_at	*ANXA7*	annexin A7	-0.563	5.2E-03
226326_at	*PCGF5*	polycomb group ring finger 5	-0.563	5.2E-03
230668_at	*NKAIN4*	Na+/K+ transporting ATPase interacting 4	-0.564	5.2E-03
228289_at	*BRD7*	bromodomain containing 7	-0.564	5.2E-03
35846_at	*THRA*	thyroid hormone receptor, alpha	-0.564	5.2E-03
225504_at	*HMBOX1*	homeobox containing 1	-0.564	5.1E-03
203657_s_at	*CTSF*	cathepsin F	-0.564	5.1E-03
226258_at	*AMN1*	antagonist of mitotic exit network 1 homolog (S. cerevisiae)	-0.564	5.1E-03
222820_at	*TNRC6C*	trinucleotide repeat containing 6C	-0.564	5.1E-03
221824_s_at	*MARCH8*	membrane-associated ring finger (C3HC4) 8, E3 ubiquitin protein ligase	-0.565	5.1E-03
219701_at	*TMOD2*	tropomodulin 2 (neuronal)	-0.565	5.1E-03
243219_x_at	*MRPL50*	mitochondrial ribosomal protein L50	-0.565	5.1E-03
209379_s_at	*FAM190B*	family with sequence similarity 190, member B	-0.565	5.1E-03
228608_at	*NALCN*	sodium leak channel, non-selective	-0.565	5.1E-03
221487_s_at	*ENSA*	endosulfine alpha	-0.565	5.1E-03
223268_at	*C11orf54*	chromosome 11 open reading frame 54	-0.565	5.0E-03
206448_at	*ZNF365*	zinc finger protein 365	-0.566	5.0E-03
212321_at	*SGPL1*	sphingosine-1-phosphate lyase 1	-0.566	5.0E-03
205156_s_at	*ASIC1*	acid-sensing (proton-gated) ion channel 1	-0.566	5.0E-03
218675_at	*SLC22A17*	solute carrier family 22, member 17	-0.566	5.0E-03
205412_at	*ACAT1*	acetyl-CoA acetyltransferase 1	-0.566	5.0E-03

Probeset ID	Gene symbol	Gene description	ρ	Adjusted *P*-value
1554145_a_at	*PPP1R21*	protein phosphatase 1, regulatory subunit 21	-0.567	4.9E-03
212837_at	*FAM175B*	family with sequence similarity 175, member B	-0.567	4.9E-03
217452_s_at	*B3GALT2*	UDP-Gal:betaGlcNAc beta 1,3-galactosyltransferase, polypeptide 2	-0.567	4.9E-03
218128_at	*NFYB*	nuclear transcription factor Y, beta	-0.567	4.9E-03
228551_at	*DENND5B*	DENN/MADD domain containing 5B	-0.567	4.9E-03
236154_at	*QKI*	QKI, KH domain containing, RNA binding	-0.567	4.9E-03
241364_at	*TMEM57*	transmembrane protein 57	-0.568	4.8E-03
212504_at	*DIP2C*	DIP2 disco-interacting protein 2 homolog C (Drosophila)	-0.568	4.8E-03
229809_at	*POU6F1*	POU class 6 homeobox 1	-0.568	4.8E-03
224435_at	*FAM213A*	family with sequence similarity 213, member A	-0.568	4.8E-03
222984_at	*PAIP2*	poly(A) binding protein interacting protein 2	-0.568	4.8E-03
228494_at	*PPP1R9A*	protein phosphatase 1, regulatory subunit 9A	-0.569	4.8E-03
209028_s_at	*ABI1*	abl-interactor 1	-0.569	4.8E-03
219451_at	*MSRB2*	methionine sulfoxide reductase B2	-0.569	4.8E-03
227456_s_at	*C6orf136*	chromosome 6 open reading frame 136	-0.569	4.8E-03
224933_s_at	*JMJD1C*	jumonji domain containing 1C	-0.569	4.8E-03
228403_at	*ENHO*	energy homeostasis associated	-0.569	4.7E-03
204199_at	*RALGPS1*	Ral GEF with PH domain and SH3 binding motif 1	-0.570	4.6E-03
205136_s_at	*NUFIP1*	nuclear fragile X mental retardation protein interacting protein 1	-0.570	4.6E-03
204028_s_at	*RABGAP1*	RAB GTPase activating protein 1	-0.570	4.6E-03
228709_at	*TPR*	translocated promoter region, nuclear basket protein	-0.570	4.6E-03
231864_at	*ZNF33A*	zinc finger protein 33A	-0.571	4.6E-03
203259_s_at	*HDDC2*	HD domain containing 2	-0.571	4.6E-03
229430_at	*C8orf46*	chromosome 8 open reading frame 46	-0.571	4.6E-03
233191_at	*RUFY2*	RUN and FYVE domain containing 2	-0.571	4.6E-03
209447_at	*SYNE1*	spectrin repeat containing, nuclear envelope 1	-0.571	4.5E-03
209455_at	*FBXW11*	F-box and WD repeat domain containing 11	-0.571	4.5E-03
218947_s_at	*MTPAP*	mitochondrial poly(A) polymerase	-0.571	4.5E-03
213564_x_at	*LDHB*	lactate dehydrogenase B	-0.572	4.5E-03
222566_at	*SUV420H1*	suppressor of variegation 4-20 homolog 1 (Drosophila)	-0.572	4.5E-03
225325_at	*MFSD6*	major facilitator superfamily domain containing 6	-0.572	4.5E-03
214043_at	*PTPRD*	protein tyrosine phosphatase, receptor type, D	-0.572	4.5E-03
242196_at	*ARHGAP32*	Rho GTPase activating protein 32	-0.572	4.5E-03
37802_r_at	*FAM63B*	family with sequence similarity 63, member B	-0.572	4.5E-03
200061_s_at	*RPS24*	ribosomal protein S24	-0.573	4.4E-03
222171_s_at	*PKNOX2*	PBX/knotted 1 homeobox 2	-0.573	4.4E-03
234982_at	*UBR3*	ubiquitin protein ligase E3 component n-recognin 3 (putative)	-0.573	4.4E-03
221763_at	*JMJD1C*	jumonji domain containing 1C	-0.573	4.4E-03
207606_s_at	*ARHGAP12*	Rho GTPase activating protein 12	-0.573	4.4E-03
227934_at	*KPNA5*	karyopherin alpha 5 (importin alpha 6)	-0.574	4.4E-03
218773_s_at	*MSRB2*	methionine sulfoxide reductase B2	-0.574	4.3E-03
232161_x_at	*PTPN4*	protein tyrosine phosphatase, non-receptor type 4 (megakaryocyte)	-0.574	4.3E-03
212503_s_at	*DIP2C*	DIP2 disco-interacting protein 2 homolog C (Drosophila)	-0.574	4.3E-03
213269_at	*ZNF248*	zinc finger protein 248	-0.574	4.3E-03
238681_at	*GDPD1*	glycerophosphodiester phosphodiesterase domain	-0.574	4.3E-03

Probeset ID	Gene symbol	Gene description	ρ	Adjusted *P*-value
		containing 1		
201677_at	*C3orf37*	Chromosome 3 open reading frame 37	-0.575	4.3E-03
219336_s_at	*ASCC1*	activating signal cointegrator 1 complex subunit 1	-0.575	4.3E-03
226001_at	*KLHL5*	kelch-like 5 (Drosophila)	-0.575	4.3E-03
219196_at	*SCG3*	secretogranin III	-0.575	4.3E-03
223179_at	*YPEL3*	yippee-like 3 (Drosophila)	-0.575	4.2E-03
229245_at	*PLEKHA6*	pleckstrin homology domain containing, family A member 6	-0.576	4.2E-03
212690_at	*DDHD2*	DDHD domain containing 2	-0.576	4.2E-03
244294_at	*GTF2H5*	general transcription factor IIH, polypeptide 5	-0.576	4.2E-03
201007_at	*HADHB*	hydroxyacyl-CoA dehydrogenase/3-ketoacyl-CoA thiolase/enoyl-CoA hydratase (trifunctional protein), beta subunit	-0.576	4.2E-03
225115_at	*HIPK2*	homeodomain interacting protein kinase 2	-0.576	4.2E-03
221771_s_at	*MPHOSPH8*	M-phase phosphoprotein 8	-0.576	4.2E-03
220115_s_at	*CDH10*	cadherin 10, type 2 (T2-cadherin)	-0.577	4.1E-03
221679_s_at	*ABHD6*	abhydrolase domain containing 6	-0.577	4.1E-03
209192_x_at	*KAT5*	K(lysine) acetyltransferase 5	-0.577	4.1E-03
226030_at	*ACADSB*	acyl-CoA dehydrogenase, short/branched chain	-0.577	4.1E-03
212326_at	*VPS13D*	vacuolar protein sorting 13 homolog D (S. cerevisiae)	-0.577	4.1E-03
219961_s_at	*PLK1S1*	polo-like kinase 1 substrate 1	-0.577	4.1E-03
218174_s_at	*C10orf57*	chromosome 10 open reading frame 57	-0.577	4.1E-03
235556_at	*CREBRF*	CREB3 regulatory factor	-0.577	4.1E-03
225432_s_at	*CSRP2BP*	CSRP2 binding protein	-0.578	4.1E-03
203457_at	*STX7*	syntaxin 7	-0.578	4.1E-03
223727_at	*KCNIP2*	Kv channel interacting protein 2	-0.578	4.1E-03
228480_at	*VAPA*	VAMP (vesicle-associated membrane protein)-associated protein A, 33kDa	-0.578	4.1E-03
203562_at	*FEZ1*	fasciculation and elongation protein zeta 1 (zygin I)	-0.578	4.0E-03
235867_at	*GSTM3*	glutathione S-transferase mu 3 (brain)	-0.578	4.0E-03
202741_at	*PRKACB*	protein kinase, cAMP-dependent, catalytic, beta	-0.579	4.0E-03
209583_s_at	*CD200*	CD200 molecule	-0.579	4.0E-03
211941_s_at	*PEBP1*	phosphatidylethanolamine binding protein 1	-0.580	3.9E-03
218791_s_at	*C15orf29*	chromosome 15 open reading frame 29	-0.580	3.9E-03
220985_s_at	*RNF170*	ring finger protein 170	-0.582	3.7E-03
212636_at	*QKI*	QKI, KH domain containing, RNA binding	-0.582	3.7E-03
208940_at	*SEPHS1*	selenophosphate synthetase 1	-0.582	3.7E-03
228711_at	*ZNF37A*	zinc finger protein 37A	-0.582	3.7E-03
223192_at	*SLC25A28*	solute carrier family 25 (mitochondrial iron transporter), member 28	-0.583	3.6E-03
210875_s_at	*ZEB1*	zinc finger E-box binding homeobox 1	-0.583	3.6E-03
223894_s_at	*AKTIP*	AKT interacting protein	-0.584	3.6E-03
227455_at	*C6orf136*	chromosome 6 open reading frame 136	-0.584	3.5E-03
213744_at	*ATRNL1*	attractin-like 1	-0.584	3.5E-03
203599_s_at	*WBP4*	WW domain binding protein 4 (formin binding protein 21)	-0.584	3.5E-03
209046_s_at	*GABARAPL2*	GABA(A) receptor-associated protein-like 2	-0.585	3.4E-03
212419_at	*ZCCHC24*	zinc finger, CCHC domain containing 24	-0.585	3.4E-03
235079_at	*ZNF704*	zinc finger protein 704	-0.585	3.4E-03
202080_s_at	*TRAK1*	trafficking protein, kinesin binding 1	-0.585	3.4E-03

Probeset ID	Gene symbol	Gene description	*ρ*	Adjusted *P*-value
208869_s_at	*GABARAPL1*	GABA(A) receptor-associated protein like 1	-0.585	3.4E-03
232059_at	*DSCAML1*	Down syndrome cell adhesion molecule like 1	-0.586	3.4E-03
209248_at	*GHITM*	growth hormone inducible transmembrane protein	-0.586	3.4E-03
210791_s_at	*ARHGAP32*	Rho GTPase activating protein 32	-0.587	3.3E-03
226805_at	*FITM2*	fat storage-inducing transmembrane protein 2	-0.587	3.3E-03
201321_s_at	*SMARCC2*	SWI/SNF related, matrix associated, actin dependent regulator of chromatin, subfamily c, member 2	-0.588	3.3E-03
235278_at	*MACROD2*	MACRO domain containing 2	-0.588	3.3E-03
212747_at	*ANKS1A*	ankyrin repeat and sterile alpha motif domain containing 1A	-0.588	3.2E-03
224649_x_at	*CCNY*	cyclin Y	-0.588	3.2E-03
219549_s_at	*RTN3*	reticulon 3	-0.588	3.2E-03
1553971_a_at	*GATS*	GATS, stromal antigen 3 opposite strand	-0.588	3.2E-03
209983_s_at	*NRXN2*	neurexin 2	-0.588	3.2E-03
211423_s_at	*SC5DL*	sterol-C5-desaturase (ERG3 delta-5-desaturase homolog, S. cerevisiae)-like	-0.588	3.2E-03
212905_at	*CSTF2T*	cleavage stimulation factor, 3' pre-RNA, subunit 2, 64kDa, tau variant	-0.589	3.2E-03
37953_s_at	*ASIC1*	acid-sensing (proton-gated) ion channel 1	-0.589	3.1E-03
227451_s_at	*CCDC90A*	coiled-coil domain containing 90A	-0.589	3.1E-03
203611_at	*TERF2*	telomeric repeat binding factor 2	-0.590	3.1E-03
218127_at	*NFYB*	nuclear transcription factor Y, beta	-0.590	3.1E-03
224581_s_at	*NUCKS1*	nuclear casein kinase and cyclin-dependent kinase substrate 1	-0.590	3.1E-03
214258_x_at	*KAT5*	K(lysine) acetyltransferase 5	-0.590	3.1E-03
221527_s_at	*PARD3*	par-3 partitioning defective 3 homolog (C. elegans)	-0.591	3.1E-03
238739_at	*IPMK*	inositol polyphosphate multikinase	-0.591	3.1E-03
1554609_at	*LOC100287896*	uncharacterized LOC100287896	-0.591	3.1E-03
223886_s_at	*RNF146*	ring finger protein 146	-0.591	3.1E-03
219767_s_at	*CRYZL1*	crystallin, zeta (quinone reductase)-like 1	-0.591	3.1E-03
240214_at	*RWDD1*	RWD domain containing 1	-0.591	3.0E-03
203427_at	*ASF1A*	ASF1 anti-silencing function 1 homolog A (S. cerevisiae)	-0.591	3.0E-03
201778_s_at	*KIAA0494*	KIAA0494	-0.592	3.0E-03
212289_at	*ANKRD12*	ankyrin repeat domain 12	-0.592	3.0E-03
210740_s_at	*ITPK1*	inositol-tetrakisphosphate 1-kinase	-0.592	3.0E-03
209618_at	*CTNND2*	catenin (cadherin-associated protein), delta 2 (neural plakophilin-related arm-repeat protein)	-0.592	3.0E-03
218789_s_at	*C11orf71*	chromosome 11 open reading frame 71	-0.592	3.0E-03
233873_x_at	*MTPAP*	mitochondrial poly(A) polymerase	-0.592	3.0E-03
228185_at	*ZNF25*	zinc finger protein 25	-0.592	3.0E-03
218878_s_at	*SIRT1*	sirtuin 1	-0.592	3.0E-03
228697_at	*HINT3*	histidine triad nucleotide binding protein 3	-0.593	3.0E-03
228354_at	*MORN4*	MORN repeat containing 4	-0.593	3.0E-03
236178_at	*C6orf162*	chromosome 6 open reading frame 162	-0.593	3.0E-03
203870_at	*USP46*	ubiquitin specific peptidase 46	-0.593	3.0E-03
204732_s_at	*TRIM23*	tripartite motif containing 23	-0.594	3.0E-03
227407_at	*TAPT1*	transmembrane anterior posterior transformation 1	-0.594	3.0E-03
222562_s_at	*TNKS2*	tankyrase, TRF1-interacting ankyrin-related ADP-ribose polymerase 2	-0.594	2.9E-03
203632_s_at	*GPRC5B*	G protein-coupled receptor, family C, group 5, member B	-0.594	2.9E-03

Probeset ID	Gene symbol	Gene description	ρ	Adjusted *P*-value
211515_s_at	*DSTYK*	dual serine/threonine and tyrosine protein kinase	-0.594	2.9E-03
204813_at	*MAPK10*	mitogen-activated protein kinase 10	-0.595	2.9E-03
240433_x_at	*CADM2*	cell adhesion molecule 2	-0.595	2.9E-03
207971_s_at	*CEP68*	centrosomal protein 68kDa	-0.595	2.9E-03
220999_s_at	*CYFIP2*	cytoplasmic FMR1 interacting protein 2	-0.595	2.9E-03
208030_s_at	*ADD1*	adducin 1 (alpha)	-0.596	2.9E-03
212989_at	*SGMS1*	sphingomyelin synthase 1	-0.596	2.9E-03
225509_at	*SAP30L*	SAP30-like	-0.596	2.9E-03
213838_at	*NOL7*	nucleolar protein 7, 27kDa	-0.596	2.8E-03
234947_s_at	*FAM204A*	family with sequence similarity 204, member A	-0.596	2.8E-03
226537_at	*HINT3*	histidine triad nucleotide binding protein 3	-0.596	2.8E-03
204063_s_at	*ULK2*	unc-51-like kinase 2 (C. elegans)	-0.596	2.8E-03
235061_at	*PPM1K*	protein phosphatase, Mg2+/Mn2+ dependent, 1K	-0.596	2.8E-03
216218_s_at	*PLCL2*	phospholipase C-like 2	-0.597	2.8E-03
222431_at	*SPIN1*	spindlin 1	-0.597	2.8E-03
203449_s_at	*TERF1*	telomeric repeat binding factor (NIMA-interacting) 1	-0.597	2.8E-03
223315_at	*NTN4*	netrin 4	-0.597	2.8E-03
228259_s_at	*EPB41L4A-AS1*	EPB41L4A antisense RNA 1 (non-protein coding)	-0.597	2.8E-03
208055_s_at	*HERC4*	HECT and RLD domain containing E3 ubiquitin protein ligase 4	-0.598	2.8E-03
209197_at	*SYT11*	synaptotagmin XI	-0.598	2.8E-03
221221_s_at	*KLHL3*	kelch-like 3 (Drosophila)	-0.598	2.8E-03
213745_at	*ATRNL1*	attractin-like 1	-0.598	2.8E-03
219046_s_at	*PKNOX2*	PBX/knotted 1 homeobox 2	-0.598	2.8E-03
221590_s_at	*ALDH6A1*	aldehyde dehydrogenase 6 family, member A1	-0.598	2.8E-03
201030_x_at	*LDHB*	lactate dehydrogenase B	-0.598	2.8E-03
237802_at	*XKR4*	XK, Kell blood group complex subunit-related family, member 4	-0.599	2.8E-03
201753_s_at	*ADD3*	adducin 3 (gamma)	-0.599	2.7E-03
203156_at	*AKAP11*	A kinase (PRKA) anchor protein 11	-0.599	2.7E-03
201957_at	*PPP1R12B*	protein phosphatase 1, regulatory subunit 12B	-0.599	2.7E-03
209818_s_at	*HABP4*	hyaluronan binding protein 4	-0.599	2.7E-03
219945_at	*DDX25*	DEAD (Asp-Glu-Ala-Asp) box helicase 25	-0.599	2.7E-03
226607_at	*C20orf194*	chromosome 20 open reading frame 194	-0.599	2.7E-03
213034_at	*SIK3*	SIK family kinase 3	-0.600	2.7E-03
223406_x_at	*FAM192A*	family with sequence similarity 192, member A	-0.600	2.7E-03
209469_at	*GPM6A*	glycoprotein M6A	-0.600	2.7E-03
223132_s_at	*TRIM8*	tripartite motif containing 8	-0.600	2.7E-03
223672_at	*SGIP1*	SH3-domain GRB2-like (endophilin) interacting protein 1	-0.601	2.7E-03
212190_at	*SERPINE2*	serpin peptidase inhibitor, clade E (nexin, plasminogen activator inhibitor type 1), member 2	-0.601	2.6E-03
214053_at	*ERBB4*	v-erb-a erythroblastic leukemia viral oncogene homolog 4 (avian)	-0.601	2.6E-03
226203_at	*MYO9A*	myosin IXA	-0.602	2.6E-03
226741_at	*SLC12A6*	solute carrier family 12 (potassium/chloride transporters), member 6	-0.602	2.6E-03
203869_at	*USP46*	ubiquitin specific peptidase 46	-0.602	2.6E-03
218264_at	*BCCIP*	BRCA2 and CDKN1A interacting protein	-0.603	2.6E-03
229317_at	*KPNA5*	karyopherin alpha 5 (importin alpha 6)	-0.603	2.5E-03

Probeset ID	Gene symbol	Gene description	ρ	Adjusted *P*-value
212764_at	*ZEB1*	zinc finger E-box binding homeobox 1	-0.603	2.5E-03
223283_s_at	*TSHZ1*	teashirt zinc finger homeobox 1	-0.603	2.5E-03
223336_s_at	*RAB18*	RAB18, member RAS oncogene family	-0.603	2.5E-03
225511_at	*GPRC5B*	G protein-coupled receptor, family C, group 5, member B	-0.604	2.5E-03
204447_at	*PROSAPIP1*	ProSAPiP1 protein	-0.604	2.5E-03
202137_s_at	*ZMYND11*	zinc finger, MYND-type containing 11	-0.604	2.5E-03
218032_at	*SNN*	stannin	-0.604	2.5E-03
225825_at	*C20orf194*	chromosome 20 open reading frame 194	-0.604	2.5E-03
235118_at	*CADM2*	cell adhesion molecule 2	-0.604	2.5E-03
203950_s_at	*CLCN6*	chloride channel, voltage-sensitive 6	-0.604	2.5E-03
209155_s_at	*NT5C2*	5'-nucleotidase, cytosolic II	-0.605	2.5E-03
221213_s_at	*ZNF280D*	zinc finger protein 280D	-0.605	2.5E-03
235561_at	*TXNL1*	thioredoxin-like 1	-0.605	2.5E-03
212606_at	*WDFY3*	WD repeat and FYVE domain containing 3	-0.605	2.5E-03
218909_at	*RPS6KC1*	ribosomal protein S6 kinase, 52kDa, polypeptide 1	-0.605	2.5E-03
205773_at	*CPEB3*	cytoplasmic polyadenylation element binding protein 3	-0.607	2.4E-03
223340_at	*ATL1*	atlastin GTPase 1	-0.607	2.4E-03
225354_s_at	*SH3BGRL2*	SH3 domain binding glutamic acid-rich protein like 2	-0.608	2.3E-03
208990_s_at	*HNRNPH3*	heterogeneous nuclear ribonucleoprotein H3 (2H9)	-0.608	2.3E-03
226684_at	*ATG2B*	autophagy related 2B	-0.608	2.3E-03
235866_at	*C9orf85*	chromosome 9 open reading frame 85	-0.608	2.3E-03
228999_at	*CHD2*	chromodomain helicase DNA binding protein 2	-0.608	2.3E-03
221523_s_at	*RRAGD*	Ras-related GTP binding D	-0.609	2.3E-03
224850_at	*ATAD1*	ATPase family, AAA domain containing 1	-0.609	2.3E-03
225368_at	*HIPK2*	homeodomain interacting protein kinase 2	-0.609	2.3E-03
227169_at	*DNAJC18*	DnaJ (Hsp40) homolog, subfamily C, member 18	-0.609	2.3E-03
203261_at	*DCTN6*	dynactin 6	-0.610	2.3E-03
224977_at	*C6orf89*	chromosome 6 open reading frame 89	-0.610	2.3E-03
1552733_at	*KLHDC1*	kelch domain containing 1	-0.610	2.3E-03
224787_s_at	*RAB18*	RAB18, member RAS oncogene family	-0.611	2.2E-03
224648_at	*GPBP1*	GC-rich promoter binding protein 1	-0.611	2.2E-03
227572_at	*USP30*	ubiquitin specific peptidase 30	-0.611	2.2E-03
225913_at	*PEAK1*	NKF3 kinase family member	-0.612	2.2E-03
225845_at	*ZBTB44*	zinc finger and BTB domain containing 44	-0.612	2.2E-03
221552_at	*ABHD6*	abhydrolase domain containing 6	-0.612	2.2E-03
209504_s_at	*PLEKHB1*	pleckstrin homology domain containing, family B (evectins) member 1	-0.613	2.2E-03
201344_at	*UBE2D2*	ubiquitin-conjugating enzyme E2D 2	-0.613	2.2E-03
224377_s_at	*RAB18*	RAB18, member RAS oncogene family	-0.614	2.1E-03
218306_s_at	*HERC1*	HECT and RLD domain containing E3 ubiquitin protein ligase family member 1	-0.614	2.1E-03
205202_at	*PCMT1*	protein-L-isoaspartate (D-aspartate) O-methyltransferase	-0.614	2.1E-03
202022_at	*ALDOC*	aldolase C, fructose-bisphosphate	-0.614	2.1E-03
202405_at	*TIAL1*	TIA1 cytotoxic granule-associated RNA binding protein-like 1	-0.614	2.1E-03
216903_s_at	*MICU1*	mitochondrial calcium uptake 1	-0.614	2.1E-03
1558759_s_at	*CLASP2*	cytoplasmic linker associated protein 2	-0.614	2.1E-03
209027_s_at	*ABI1*	abl-interactor 1	-0.615	2.1E-03
235780_at	*PRKACB*	protein kinase, cAMP-dependent, catalytic, beta	-0.615	2.1E-03

Probeset ID	Gene symbol	Gene description	ρ	Adjusted *P*-value
213222_at	*PLCB1*	phospholipase C, beta 1 (phosphoinositide-specific)	-0.616	2.1E-03
227154_at	*IGSF21*	immunoglobin superfamily, member 21	-0.616	2.0E-03
203845_at	*KAT2B*	K(lysine) acetyltransferase 2B	-0.617	2.0E-03
227351_at	*C16orf52*	chromosome 16 open reading frame 52	-0.617	2.0E-03
225768_at	*NR1D2*	nuclear receptor subfamily 1, group D, member 2	-0.618	2.0E-03
208881_x_at	*IDI1*	isopentenyl-diphosphate delta isomerase 1	-0.618	2.0E-03
213208_at	*KIAA0240*	KIAA0240	-0.618	2.0E-03
226148_at	*ZBTB44*	zinc finger and BTB domain containing 44	-0.618	2.0E-03
213489_at	*MAPRE2*	microtubule-associated protein, RP/EB family, member 2	-0.619	2.0E-03
224780_at	*RBM17*	RNA binding motif protein 17	-0.620	1.9E-03
209271_at	*BPTF*	bromodomain PHD finger transcription factor	-0.620	1.9E-03
227049_at	*ZADH2*	zinc binding alcohol dehydrogenase domain containing 2	-0.620	1.9E-03
227949_at	*PHACTR3*	phosphatase and actin regulator 3	-0.620	1.9E-03
221702_s_at	*TM2D3*	TM2 domain containing 3	-0.621	1.9E-03
225116_at	*HIPK2*	homeodomain interacting protein kinase 2	-0.621	1.9E-03
205669_at	*NCAM2*	neural cell adhesion molecule 2	-0.621	1.9E-03
209538_at	*ZNF32*	zinc finger protein 32	-0.621	1.9E-03
227209_at	*CNTN1*	contactin 1	-0.621	1.9E-03
204290_s_at	*ALDH6A1*	aldehyde dehydrogenase 6 family, member A1	-0.621	1.9E-03
213364_s_at	*SNX1*	sorting nexin 1	-0.623	1.8E-03
226964_at	*TTBK2*	tau tubulin kinase 2	-0.623	1.8E-03
205638_at	*BAI3*	brain-specific angiogenesis inhibitor 3	-0.624	1.8E-03
208857_s_at	*PCMT1*	protein-L-isoaspartate (D-aspartate) O-methyltransferase	-0.624	1.8E-03
235798_at	*TMEM170B*	transmembrane protein 170B	-0.624	1.8E-03
1553321_a_at	*SULT1C4*	sulfotransferase family, cytosolic, 1C, member 4	-0.624	1.8E-03
205839_s_at	*BZRAP1*	benzodiazapine receptor (peripheral) associated protein 1	-0.625	1.8E-03
200970_s_at	*SERP1*	stress-associated endoplasmic reticulum protein 1	-0.625	1.8E-03
1554547_at	*FAM13C*	family with sequence similarity 13, member C	-0.626	1.7E-03
217286_s_at	*NDRG3*	NDRG family member 3	-0.626	1.7E-03
227728_at	*PPM1A*	protein phosphatase, Mg2+/Mn2+ dependent, 1A	-0.626	1.7E-03
241365_at	*SATB1*	SATB homeobox 1	-0.626	1.7E-03
212867_at	*NCOA2*	nuclear receptor coactivator 2	-0.626	1.7E-03
209065_at	*UQCRB*	ubiquinol-cytochrome c reductase binding protein	-0.627	1.7E-03
212080_at	*MLL*	myeloid/lymphoid or mixed-lineage leukemia (trithorax homolog, Drosophila)	-0.627	1.7E-03
203831_at	*R3HDM2*	R3H domain containing 2	-0.627	1.7E-03
1555250_a_at	*CPEB3*	cytoplasmic polyadenylation element binding protein 3	-0.628	1.7E-03
1554132_a_at	*FAM190B*	family with sequence similarity 190, member B	-0.628	1.7E-03
226358_at	*APH1B*	anterior pharynx defective 1 homolog B (C. elegans)	-0.629	1.6E-03
212675_s_at	*CEP68*	centrosomal protein 68kDa	-0.630	1.6E-03
226680_at	*IKZF5*	IKAROS family zinc finger 5 (Pegasus)	-0.630	1.6E-03
228390_at	*RAB30*	RAB30, member RAS oncogene family	-0.630	1.6E-03
226977_at	*IGIP*	IgA-inducing protein homolog (Bos taurus)	-0.630	1.6E-03
201034_at	*ADD3*	adducin 3 (gamma)	-0.630	1.6E-03
204615_x_at	*IDI1*	isopentenyl-diphosphate delta isomerase 1	-0.630	1.6E-03
224248_x_at	*FAM192A*	family with sequence similarity 192, member A	-0.630	1.6E-03
213433_at	*ARL3*	ADP-ribosylation factor-like 3	-0.631	1.6E-03
213555_at	*RWDD2A*	RWD domain containing 2A	-0.631	1.6E-03
224645_at	*EIF4EBP2*	eukaryotic translation initiation factor 4E binding protein	-0.631	1.6E-03

Probeset ID	Gene symbol	Gene description	ρ	Adjusted *P*-value
		2		
205280_at	*GLRB*	glycine receptor, beta	-0.632	1.6E-03
213093_at	*PRKCA*	protein kinase C, alpha	-0.632	1.6E-03
225313_at	*FAM217B*	family with sequence similarity 217, member B	-0.632	1.6E-03
213411_at	*ADAM22*	ADAM metallopeptidase domain 22	-0.632	1.6E-03
210976_s_at	*PFKM*	phosphofructokinase, muscle	-0.632	1.6E-03
201425_at	*ALDH2*	aldehyde dehydrogenase 2 family (mitochondrial)	-0.632	1.6E-03
1554161_at	*SLC25A27*	solute carrier family 25, member 27	-0.632	1.6E-03
206453_s_at	*NDRG2*	NDRG family member 2	-0.632	1.6E-03
235072_s_at	*KIF13A*	kinesin family member 13A	-0.633	1.6E-03
211994_at	*WNK1*	WNK lysine deficient protein kinase 1	-0.634	1.6E-03
212423_at	*ZCCHC24*	zinc finger, CCHC domain containing 24	-0.634	1.6E-03
202432_at	*PPP3CB*	protein phosphatase 3, catalytic subunit, beta isozyme	-0.634	1.6E-03
225097_at	*HIPK2*	homeodomain interacting protein kinase 2	-0.634	1.6E-03
229463_at	*NTRK2*	neurotrophic tyrosine kinase, receptor, type 2	-0.634	1.6E-03
225751_at	*RBM17*	RNA binding motif protein 17	-0.635	1.5E-03
228967_at	*EIF1*	eukaryotic translation initiation factor 1	-0.635	1.5E-03
200899_s_at	*MGEA5*	meningioma expressed antigen 5 (hyaluronidase)	-0.636	1.5E-03
214279_s_at	*NDRG2*	NDRG family member 2	-0.637	1.4E-03
203619_s_at	*FAIM2*	Fas apoptotic inhibitory molecule 2	-0.637	1.4E-03
210414_at	*FLRT1*	fibronectin leucine rich transmembrane protein 1	-0.638	1.4E-03
226774_at	*FAM120B*	family with sequence similarity 120B	-0.638	1.4E-03
205359_at	*AKAP6*	A kinase (PRKA) anchor protein 6	-0.638	1.4E-03
212508_at	*MOAP1*	modulator of apoptosis 1	-0.639	1.4E-03
221623_at	*BCAN*	brevican	-0.640	1.4E-03
227611_at	*TARSL2*	threonyl-tRNA synthetase-like 2	-0.640	1.4E-03
238454_at	*ZNF540*	zinc finger protein 540	-0.640	1.4E-03
228857_at	*GNL1*	guanine nucleotide binding protein-like 1	-0.640	1.4E-03
212453_at	*KIAA1279*	KIAA1279	-0.642	1.3E-03
221556_at	*CDC14B*	CDC14 cell division cycle 14 homolog B (S. cerevisiae)	-0.642	1.3E-03
225351_at	*FAM45A*	family with sequence similarity 45, member A	-0.642	1.3E-03
227202_at	*CNTN1*	contactin 1	-0.643	1.3E-03
236095_at	*NTRK2*	neurotrophic tyrosine kinase, receptor, type 2	-0.643	1.3E-03
231031_at	*KGFLP2*	keratinocyte growth factor-like protein 2	-0.643	1.3E-03
215522_at	*SORCS3*	sortilin-related VPS10 domain containing receptor 3	-0.643	1.3E-03
229072_at	*RAB30*	RAB30, member RAS oncogene family	-0.644	1.3E-03
213271_s_at	*DOPEY1*	dopey family member 1	-0.645	1.3E-03
238497_at	*TMEM136*	transmembrane protein 136	-0.645	1.3E-03
231112_at	*SNRPE*	small nuclear ribonucleoprotein polypeptide E	-0.645	1.3E-03
241874_at	*IGIP*	IgA-inducing protein homolog (Bos taurus)	-0.646	1.2E-03
212323_s_at	*VPS13D*	vacuolar protein sorting 13 homolog D (S. cerevisiae)	-0.647	1.2E-03
223282_at	*TSHZ1*	teashirt zinc finger homeobox 1	-0.647	1.2E-03
221476_s_at	*RPL15*	ribosomal protein L15	-0.647	1.2E-03
224647_at	*CCNY*	cyclin Y	-0.647	1.2E-03
224368_s_at	*NDRG3*	NDRG family member 3	-0.648	1.2E-03
240228_at	*CSMD3*	CUB and Sushi multiple domains 3	-0.648	1.2E-03
203620_s_at	*FCHSD2*	FCH and double SH3 domains 2	-0.648	1.2E-03
212062_at	*ATP9A*	ATPase, class II, type 9A	-0.649	1.2E-03

Probeset ID	Gene symbol	Gene description	ρ	Adjusted *P*-value
223721_s_at	*DNAJC12*	DnaJ (Hsp40) homolog, subfamily C, member 12	-0.650	1.2E-03
218373_at	*AKTIP*	AKT interacting protein	-0.651	1.2E-03
215058_at	*DENND5B*	DENN/MADD domain containing 5B	-0.651	1.2E-03
203408_s_at	*SATB1*	SATB homeobox 1	-0.653	1.1E-03
215848_at	*SCAPER*	S-phase cyclin A-associated protein in the ER	-0.653	1.1E-03
214762_at	*ATP6V1G2*	ATPase, H+ transporting, lysosomal 13kDa, V1 subunit G2	-0.653	1.1E-03
239068_at	*GNL1*	guanine nucleotide binding protein-like 1	-0.654	1.1E-03
226627_at	*SEPT8*	septin 8	-0.654	1.1E-03
213549_at	*PDZD8*	PDZ domain containing 8	-0.656	1.1E-03
221486_at	*ENSA*	endosulfine alpha	-0.656	1.1E-03
224651_at	*CCNY*	cyclin Y	-0.656	1.1E-03
202364_at	*MXI1*	MAX interactor 1	-0.657	1.1E-03
212462_at	*KAT6B*	K(lysine) acetyltransferase 6B	-0.658	1.0E-03
221045_s_at	*PER3*	period homolog 3 (Drosophila)	-0.658	1.0E-03
211874_s_at	*KAT6B*	K(lysine) acetyltransferase 6B	-0.658	1.0E-03
45288_at	*ABHD6*	abhydrolase domain containing 6	-0.659	1.0E-03
225131_at	*ZRANB1*	zinc finger, RAN-binding domain containing 1	-0.659	1.0E-03
225950_at	*SAMD8*	sterile alpha motif domain containing 8	-0.660	1.0E-03
202959_at	*MUT*	methylmalonyl CoA mutase	-0.661	9.7E-04
224755_at	*TM9SF3*	transmembrane 9 superfamily member 3	-0.662	9.4E-04
218218_at	*APPL2*	adaptor protein, phosphotyrosine interaction, PH domain and leucine zipper containing 2	-0.662	9.4E-04
212308_at	*CLASP2*	cytoplasmic linker associated protein 2	-0.663	9.3E-04
214821_at	*SLC25A4*	solute carrier family 25 (mitochondrial carrier; adenine nucleotide translocator), member 4	-0.664	8.9E-04
239425_at	*DCUN1D5*	DCN1, defective in cullin neddylation 1, domain containing 5 (S. cerevisiae)	-0.665	8.9E-04
228640_at	*PCDH7*	protocadherin 7	-0.665	8.8E-04
212199_at	*MRFAP1L1*	Morf4 family associated protein 1-like 1	-0.667	8.7E-04
226527_at	*RPRD2*	regulation of nuclear pre-mRNA domain containing 2	-0.667	8.7E-04
224471_s_at	*BTRC*	beta-transducin repeat containing E3 ubiquitin protein ligase	-0.668	8.7E-04
200711_s_at	*SKP1*	S-phase kinase-associated protein 1	-0.668	8.7E-04
235360_at	*PLEKHM3*	pleckstrin homology domain containing, family M, member 3	-0.668	8.7E-04
226981_at	*MLL*	myeloid/lymphoid or mixed-lineage leukemia (trithorax homolog, Drosophila)	-0.669	8.7E-04
227341_at	*BEND7*	BEN domain containing 7	-0.670	8.7E-04
202136_at	*ZMYND11*	zinc finger, MYND-type containing 11	-0.670	8.7E-04
225138_at	*ZRANB1*	zinc finger, RAN-binding domain containing 1	-0.670	8.7E-04
223296_at	*SLC25A33*	solute carrier family 25 (pyrimidine nucleotide carrier), member 33	-0.671	8.7E-04
202641_at	*ARL3*	ADP-ribosylation factor-like 3	-0.672	8.7E-04
224653_at	*EIF4EBP2*	eukaryotic translation initiation factor 4E binding protein 2	-0.673	8.7E-04
201752_s_at	*ADD3*	adducin 3 (gamma)	-0.674	8.6E-04
230624_at	*SLC25A27*	solute carrier family 25, member 27	-0.676	8.2E-04
213463_s_at	*FAM149B1*	family with sequence similarity 149, member B1	-0.676	8.2E-04
225663_at	*ACBD5*	acyl-CoA binding domain containing 5	-0.679	7.9E-04

Probeset ID	Gene symbol	Gene description	ρ	Adjusted *P*-value
214496_x_at	*KAT6B*	K(lysine) acetyltransferase 6B	-0.680	7.8E-04
225930_at	*NKIRAS1*	NFKB inhibitor interacting Ras-like 1	-0.681	7.6E-04
205882_x_at	*ADD3*	adducin 3 (gamma)	-0.685	6.4E-04
235224_s_at	*CAND1*	cullin-associated and neddylation-dissociated 1	-0.685	6.4E-04
212078_s_at	*MLL*	myeloid/lymphoid or mixed-lineage leukemia (trithorax homolog, Drosophila)	-0.685	6.4E-04
227334_at	*USP54*	ubiquitin specific peptidase 54	-0.686	6.4E-04
203608_at	*ALDH5A1*	aldehyde dehydrogenase 5 family, member A1	-0.688	6.4E-04
235164_at	*ZNF25*	zinc finger protein 25	-0.689	6.3E-04
227585_at	*ATAD1*	ATPase family, AAA domain containing 1	-0.691	6.1E-04
212076_at	*MLL*	myeloid/lymphoid or mixed-lineage leukemia (trithorax homolog, Drosophila)	-0.691	6.1E-04
1568877_a_at	*ACBD5*	acyl-CoA binding domain containing 5	-0.692	6.1E-04
232905_at	*GTF2H5*	general transcription factor IIH, polypeptide 5	-0.693	6.1E-04
212452_x_at	*KAT6B*	K(lysine) acetyltransferase 6B	-0.694	6.1E-04
218957_s_at	*PAAF1*	proteasomal ATPase-associated factor 1	-0.698	5.3E-04
213268_at	*CAMTA1*	calmodulin binding transcription activator 1	-0.700	5.1E-04
201276_at	*RAB5B*	RAB5B, member RAS oncogene family	-0.700	5.1E-04
226352_at	*JMY*	junction mediating and regulatory protein, p53 cofactor	-0.700	5.1E-04
216399_s_at	*SCAPER*	S-phase cyclin A-associated protein in the ER	-0.701	5.1E-04
207842_s_at	*CASC3*	cancer susceptibility candidate 3	-0.704	5.1E-04
203431_s_at	*ARHGAP32*	Rho GTPase activating protein 32	-0.705	5.1E-04
209741_x_at	*SCAPER*	S-phase cyclin A-associated protein in the ER	-0.705	5.1E-04
1554784_at	*CNTN1*	contactin 1	-0.706	5.1E-04
1557073_s_at	*TTBK2*	tau tubulin kinase 2	-0.709	5.1E-04
227321_at	*GATS*	GATS, stromal antigen 3 opposite strand	-0.714	4.7E-04
219743_at	*HEY2*	hairy/enhancer-of-split related with YRPW motif 2	-0.727	3.8E-04
238115_at	*DNAJC18*	DnaJ (Hsp40) homolog, subfamily C, member 18	-0.729	3.8E-04
226186_at	*TMOD2*	tropomodulin 2 (neuronal)	-0.730	3.8E-04
203097_s_at	*RAPGEF2*	Rap guanine nucleotide exchange factor (GEF) 2	-0.743	3.8E-04

Note - Spearman's rank correlation test was used to measure the relationship between gene expression level and glioma grade. Some genes are encoded by multiple probe sets.

Supplementary Table S2. Ion channel genes that are differentially expressed with glioma grade in the UHC cohort

Probeset ID	Gene symbol	Gene description	ρ	Adjusted *P*-value
208659_at	*CLIC1*	chloride intracellular channel 1	0.473	2.3E-02
201560_at	*CLIC4*	chloride intracellular channel 4	0.431	4.3E-02
205903_s_at	*KCNN3*	potassium intermediate/small conductance calcium-activated channel, subfamily N, member 3	-0.423	4.8E-02
231103_at	*KCNN3*	potassium intermediate/small conductance calcium-activated channel, subfamily N, member 3	-0.425	4.7E-02
1555268_a_at	*GRID1*	glutamate receptor, ionotropic, delta 1	-0.457	3.0E-02
228414_at	*KCNMA1*	potassium large conductance calcium-activated channel, subfamily M, alpha member 1	-0.463	2.7E-02
1555246_a_at	*SCN1A*	sodium channel, voltage-gated, type I, alpha subunit	-0.470	2.5E-02
210078_s_at	*KCNAB1*	potassium voltage-gated channel, shaker-related subfamily, beta member 1	-0.477	2.2E-02
210108_at	*CACNA1D*	calcium channel, voltage-dependent, L type, alpha 1D subunit	-0.480	2.2E-02
1555993_at	*CACNA1D*	calcium channel, voltage-dependent, L type, alpha 1D subunit	-0.483	2.1E-02
236538_at	*GRIA2*	glutamate receptor, ionotropic, AMPA 2	-0.489	1.9E-02
211006_s_at	*KCNB1*	potassium voltage-gated channel, Shab-related subfamily, member 1	-0.501	1.5E-02
205358_at	*GRIA2*	glutamate receptor, ionotropic, AMPA 2	-0.511	1.3E-02
244680_at	*GLRB*	glycine receptor, beta	-0.511	1.3E-02
207103_at	*KCND2*	potassium voltage-gated channel, Shal-related subfamily, member 2	-0.513	1.3E-02
244623_at	*KCNQ5*	potassium voltage-gated channel, KQT-like subfamily, member 5	-0.514	1.2E-02
211662_s_at	*VDAC2*	voltage-dependent anion channel 2	-0.525	1.0E-02
207091_at	*P2RX7*	purinergic receptor P2X, ligand-gated ion channel, 7	-0.536	8.1E-03
210471_s_at	*KCNAB1*	potassium voltage-gated channel, shaker-related subfamily, beta member 1	-0.538	8.0E-03
228581_at	*KCNJ10*	potassium inwardly-rectifying channel, subfamily J, member 10	-0.544	7.2E-03
205279_s_at	*GLRB*	glycine receptor, beta	-0.559	5.6E-03
228608_at	*NALCN*	sodium leak channel, non-selective	-0.565	5.1E-03
203950_s_at	*CLCN6*	chloride channel, voltage-sensitive 6	-0.604	2.5E-03
205280_at	*GLRB*	glycine receptor, beta	-0.632	1.6E-03

Note - Spearman's rank correlation test was used to measure the relationship between gene expression level and glioma grade. Some genes are encoded by multiple probe sets.

Supplementary Table S3. Significant correlation between expression of the 18 ion channel genes and glioma grade in the HFH cohort

Probeset ID	Gene symbol	Gene description	ρ	Adjusted *P*-value
208659_at	*CLIC1*	chloride intracellular channel 1	0.672	0.0E-00
201560_at	*CLIC4*	chloride intracellular channel 4	0.555	1.6E-14
221881_s_at	*CLIC4*	chloride intracellular channel 4	0.546	5.1E-14
201559_s_at	*CLIC4*	chloride intracellular channel 4	0.536	1.6E-13
1555246_a_at	*SCN1A*	sodium channel, voltage-gated, type I, alpha subunit	-0.165	4.1E-02
205903_s_at	*KCNN3*	potassium intermediate/small conductance calcium-activated channel, subfamily N, member 3	-0.166	4.0E-02
210383_at	*SCN1A*	sodium channel, voltage-gated, type I, alpha subunit	-0.201	1.2E-02
231103_at	*KCNN3*	potassium intermediate/small conductance calcium-activated channel, subfamily N, member 3	-0.294	1.6E-04
207091_at	*P2RX7*	purinergic receptor P2X, ligand-gated ion channel, 7	-0.302	1.0E-04
207103_at	*KCND2*	potassium voltage-gated channel, Shal-related subfamily, member 2	-0.322	3.2E-05
211662_s_at	*VDAC2*	voltage-dependent anion channel 2	-0.443	3.2E-09
221584_s_at	*KCNMA1*	potassium large conductance calcium-activated channel, subfamily M, alpha member 1	-0.447	2.2E-09
205279_s_at	*GLRB*	glycine receptor, beta	-0.496	1.6E-11
228581_at	*KCNJ10*	potassium inwardly-rectifying channel, subfamily J, member 10	-0.521	9.8E-13
205280_at	*GLRB*	glycine receptor, beta	-0.525	6.4E-13
210108_at	*CACNA1D*	calcium channel, voltage-dependent, L type, alpha 1D subunit	-0.543	7.0E-14
244623_at	*KCNQ5*	potassium voltage-gated channel, KQT-like subfamily, member 5	-0.556	1.3E-14
228414_at	*KCNMA1*	potassium large conductance calcium-activated channel, subfamily M, alpha member 1	-0.571	1.7E-15
244680_at	*GLRB*	glycine receptor, beta	-0.586	2.2E-16
203950_s_at	*CLCN6*	chloride channel, voltage-sensitive 6	-0.586	2.2E-16
211006_s_at	*KCNB1*	potassium voltage-gated channel, Shab-related subfamily, member 1	-0.591	9.2E-17
210471_s_at	*KCNAB1*	potassium voltage-gated channel, shaker-related subfamily, beta member 1	-0.601	2.0E-17
205358_at	*GRIA2*	glutamate receptor, ionotropic, AMPA 2	-0.605	1.0E-17
210078_s_at	*KCNAB1*	potassium voltage-gated channel, shaker-related subfamily, beta member 1	-0.608	6.5E-18
236538_at	*GRIA2*	glutamate receptor, ionotropic, AMPA 2	-0.630	1.5E-19
1555268_a_at	*GRID1*	glutamate receptor, ionotropic, delta 1	-0.656	1.3E-21
1555993_at	*CACNA1D*	calcium channel, voltage-dependent, L type, alpha 1D subunit	-0.665	2.2E-22
228608_at	*NALCN*	sodium leak channel, non-selective	-0.687	2.0E-24

Note - Spearman's rank correlation test was used to measure the relationship between gene expression level and glioma grade. Some genes are encoded by multiple probe sets.
